# Genome-wide association studies dissect the genetic networks underlying agronomical traits in soybean

**DOI:** 10.1186/s13059-017-1289-9

**Published:** 2017-08-24

**Authors:** Chao Fang, Yanming Ma, Shiwen Wu, Zhi Liu, Zheng Wang, Rui Yang, Guanghui Hu, Zhengkui Zhou, Hong Yu, Min Zhang, Yi Pan, Guoan Zhou, Haixiang Ren, Weiguang Du, Hongrui Yan, Yanping Wang, Dezhi Han, Yanting Shen, Shulin Liu, Tengfei Liu, Jixiang Zhang, Hao Qin, Jia Yuan, Xiaohui Yuan, Fanjiang Kong, Baohui Liu, Jiayang Li, Zhiwu Zhang, Guodong Wang, Baoge Zhu, Zhixi Tian

**Affiliations:** 1 State Key Laboratory of Plant Cell and Chromosome Engineering, Institute of Genetics and Developmental Biology, Chinese Academy of Sciences, Beijing, 100101 China; 2 State Key Laboratory of Plant Genomics, Institute of Genetics and Developmental Biology, Chinese Academy of Sciences, Beijing, 100101 China; 3 Institute of maize research, Heilongjiang Academy of Agricultural Sciences, Harbin, 150086 China; 4 Institute of Animal Science, Chinese Academy of Agricultural Sciences, Beijing, 100193 China; 5 Mudanjiang Branch of Heilongjiang Academy of Agricultural Sciences, Mudanjiang, 157041 China; 6 Institute of Soybean Research, Heilongjiang Academy of Agricultural Sciences, Harbin, 150086 China; 7 Heihe Branch of Heilongjiang Academy of Agricultural Sciences, Heihe, 164300 China; 8 School of Computer Science and Technology, Wuhan University of Technology, Wuhan, 430070 China; 9 Key Laboratory of Soybean Molecular Design Breeding, Northeast Institute of Geography and Agroecology, Chinese Academy of Sciences, Harbin, 130102 China; 10 Department of Crop and Soil Sciences, Washington State University, Pullman, WA 99164 USA; 11 University of Chinese Academy of Sciences, Beijing, 100039 China

**Keywords:** Soybean, Agronomic traits, GWAS, Network

## Abstract

**Background:**

Soybean (*Glycine max* [L.] Merr.) is one of the most important oil and protein crops. Ever-increasing soybean consumption necessitates the improvement of varieties for more efficient production. However, both correlations among different traits and genetic interactions among genes that affect a single trait pose a challenge to soybean breeding.

**Results:**

To understand the genetic networks underlying phenotypic correlations, we collected 809 soybean accessions worldwide and phenotyped them for two years at three locations for 84 agronomic traits. Genome-wide association studies identified 245 significant genetic loci, among which 95 genetically interacted with other loci. We determined that 14 oil synthesis-related genes are responsible for fatty acid accumulation in soybean and function in line with an additive model. Network analyses demonstrated that 51 traits could be linked through the linkage disequilibrium of 115 associated loci and these links reflect phenotypic correlations. We revealed that 23 loci, including the known *Dt1*, *E2*, *E1*, *Ln*, *Dt2*, *Fan*, and *Fap* loci, as well as 16 undefined associated loci, have pleiotropic effects on different traits.

**Conclusions:**

This study provides insights into the genetic correlation among complex traits and will facilitate future soybean functional studies and breeding through molecular design.

**Electronic supplementary material:**

The online version of this article (doi:10.1186/s13059-017-1289-9) contains supplementary material, which is available to authorized users.

## Background

Soybean (*Glycine max* [L.] Merr.) is a major crop of agronomic importance as a predominant source of protein and oil [1]. To meet the needs of the rapidly increasing human population, soybean breeders are challenged with finding a high-efficiency breeding strategy for developing soybean varieties with higher yield and improved quality [2]. Molecular breeding has been proposed to be a powerful and effective approach for crop breeding, but requires a better understanding of the genetic architecture and networks underlying agronomical traits [3, 4]. Therefore, a priority task for accelerating the development of soybean varieties is a global dissection of the genetic basis of agronomical traits.

Quantitative trait loci (QTL) and positional cloning identified a set of loci that are responsible for flowering and maturity, biotic and abiotic stresses, and growth habits (see review from Xia et al. [5]). However, our understanding of the genetic regulation of agronomic traits remains limited because most of them are naturally adapted into complex traits [6]. Genome-wide association study (GWAS) is a powerful approach for dissecting complex traits [7] and has been successfully applied for the study of many plants, such as *Arabidopsis* [8], rice [9–11], maize [12, 13], and foxtail millet [14]. In soybean, genotyping by either the Illumina Bead Chip or specific locus amplified fragment sequencing, the evaluation of several specific agronomic traits, including seed protein and oil concentration [15, 16], sudden death syndrome resistance [17], cyst nematode resistance [18, 19], and flowering time [20] were conducted through GWAS. These studies provided valuable resources for future molecular breeding of soybean.

Nevertheless, the dissection of a specific trait is insufficient for molecular breeding because many complex traits exhibit correlation and tend to be tightly integrated, resulting in heritable covariation [21, 22], which add the complexity for breeding [23]. For instance, it is difficult to simultaneously increase grain yield and protein content for most crops because these two traits exhibit negative correlation and tend to change together [24–26]. The objectives of soybean breeding have expanded beyond yield; in fact, multiple selection criteria including oil content and protein content have been applied. Therefore, an understanding of how traits covariation is essential for the genetic improvement of multiple complex traits [27].

In this study, we collected 809 diverse soybean accessions, cultivated them at three locations for two years, and phenotyped them for 84 agronomic traits. Whole-genome sequencing (WGS) at an 8.3 × depth produced more than 11 million genetic markers. The endeavor from comprehensive GWAS analyses enabled the identification of the underlying genetic loci, loci interaction, and genetic networks across traits.

## Results

### Genotyping and phenotyping of 809 diverse soybean accessions

**Fig. 1:**
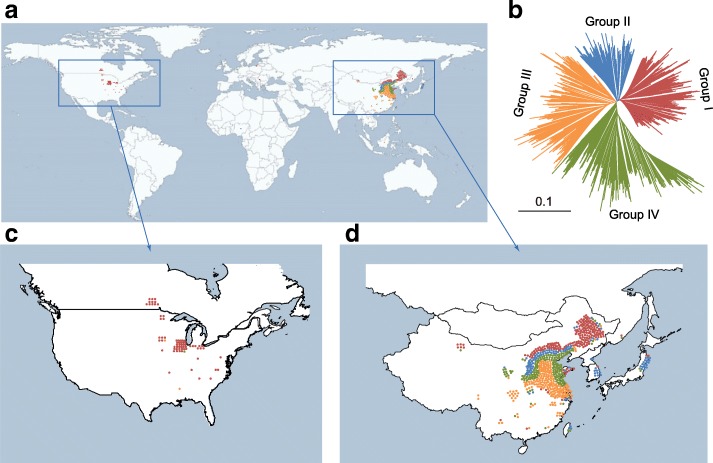
Geographic distribution and genetic structure of 809 soybean accessions. **a** Geographic distribution of the 809 soybean accessions. Each accession is displayed as a *dot*. **b** Genetic structure of the 809 soybean accessions. The accessions are clustered by the neighbor-joining tree using whole-genome SNPs. The length of the lines on the tree indicates the simple matching distance. **c**, **d** The areas with dense collections (Asia and North America) are magnified separately. The colors of the *dots* in (**a**, **c**, and **d**) correspond to their groups in (**b**)

On the basis of our previous investigated 130 landraces and 110 cultivars [28], we collected additional 291 landraces and 278 cultivars in this study, which composed a population with a total of 809 soybean accessions (Additional file 1: Table S1). The population consisted of 70 previously reported representative accessions [29], 160 Chinese core collection accessions [30], and 579 other accessions from different countries and regions. The 421 landraces and the 388 cultivars covered the main soybean producing areas, including China, Korea, Japan, Russia, the United States, and Canada, but not South America (Fig. 1a; Additional file 1: Table S1). Of the 809 accessions, 240 were sequenced in a previous study and the other 569 lines were sequenced in the present study. In total, 66.8 billion paired-end reads (7.0 Tb of sequences) were generated with a mean depth of approximately 8.3 × for each accession (Additional file 1: Table S1). After mapping against the reference genome, single-nucleotide polymorphism (SNP) calling, and imputation (see “Methods”), a total of 10,415,168 SNPs and 1,033,071 small indels (≤6 bp) were identified (Additional file 2: Table S2). To assess the quality of the genotype data, we validated 37 randomly selected SNPs in 96 accessions using the Sanger method (see “Methods”) and the results demonstrated that the accuracy of the identified SNPs was 99.8% (Additional file 3: Table S3; Additional file 4: Table S4).

The neighbor-joining tree suggested that the 809 accessions could be classified into four main clades (Fig. 1b), which were associated with their geographical distribution (Fig. 1c and d). An investigation of population structures with varying levels of K-means using fastStructure [31] also predicted that the optimal number of subpopulations was approximately K = 4 (Additional file 5: Figure S1). The analyses suggested that the accessions exhibited a subpopulation structure, which was used as a covariate within the GWAS model.

We grew all of the 809 accessions in Beijing for two years (in 2013 and 2014). We assayed 45 morphology traits each year, including those related to yield, color, architecture, organ shape, and growth period (Additional file 6: Table S5). In 2013, we also measured 39 nutrient composition traits that related to oil content, protein content, fatty acid components, and amino acid components (Additional file 6: Table S5) through gas chromatography–mass spectrometry (GC-MS).

Soybean grows across a range of latitudes from 50°N to 35°S [32]. We found significant differences in some of the traits, such as those related to the growth period, architecture, yield, and nutrient composition, between the accessions from higher latitudes (above 40.5°N) and those from lower latitudes (below 40.5°N) (Additional file 5: Figure S2). These differences may have been caused by the tendency of soybean to adapt to a limited latitudinal region due to its photoperiod sensitivity [33]. As a result, we replanted the accessions that from high latitudes (n = 275) at a location northeast to Beijing (Mudanjiang, Heilongjiang Province) and the rest from low latitudes (534) at a southern location (Zhoukou, Henan Province) to fully assess their potentials. For both locations, most of the morphology trait measurements were repeated in 2014 and 2015, and the nutrient composition trait measurements were repeated in 2014. The overall performances of the 809 accessions were predicted as the best linear unbiased prediction (BLUP) using a mixed linear model (MLM), which was implemented using the lme4 package for R.

### Whole-genome screening for significantly associated loci (SAL)

We conducted a GWAS on the 84 traits based on more than four million of the markers (SNPs with a minor allele frequency [MAF] ≥ 0.05]) genotyped from the 809 accessions through a MLM implemented in Efficient Mixed-Model Association eXpedited (EMMAX) software. The population structure was represented by the first three principal components, which was fitted as fixed effects. Kinship was used to define the variance structure of the random variables for the total genetic effects of the 809 accessions. No inflated *P* values were found and most markers (99%) exhibited *P* values equal to those expected under the null hypothesis, suggesting that the MLM controlled population structure and cryptic relationships well. To control both false positives and false negatives, we also conducted permutation tests by randomly shuffling the phenotypes to break their relationship with genotypes to derive a genome-wide threshold (see “Methods” and Additional file 7: Table S6). By using the empirical threshold, we identified 150 SAL that significantly associated with 57 of the 84 traits, using all 809 accessions (Additional file 8: Table S7; Additional file 5: Figures S3–86).

**Fig. 2:**
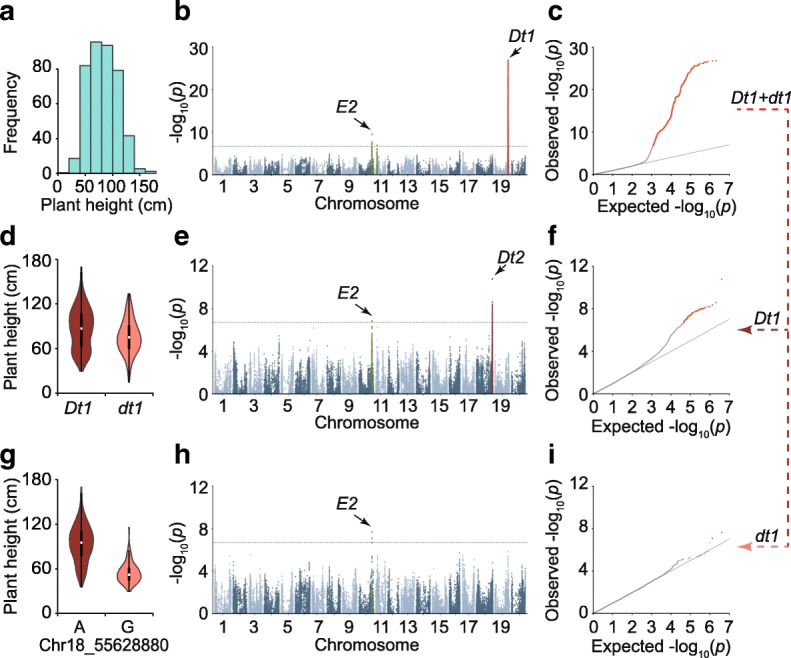
GWAS of the soybean plant height. **a** Distribution of the plant height values across all of the 809 soybean accessions. **b** GWAS result from all accessions. In the GWAS result, both known genes *Dt1* and *E2* are identified. **c** Quantile–quantile plot for plant height. **d** The plant height variation between different *Dt1* alleles in all 809 accessions. The known gene *Dt1* separates the 809 accessions into two subgroups with different plant height means. **e** The GWAS result of plant height using the accessions from the *Dt1* subgroup. **f** Quantile–quantile plot for plant height of *Dt1* subgroup. **g** Plant height variation between different *Dt2* genotypes in the *Dt1* subgroup. **h** The GWAS result of plant height using the accessions from the *dt1* subgroup. **i** Quantile–quantile plot for plant height of *dt1* subgroup. GWAS results are presented by negative log_10_ *P* values against position on each of 20 chromosomes. *Horizontal dashed lines* indicate the genome-wide significant threshold (2 × 10^–7^)

Epistasis, or the interaction between genes, plays an important role in controlling complex inheritance [34]. For instance, *Dt1* exerts an epistatic effect on *Dt2* in the regulation of plant height in soybean [35, 36]. In this study, we detected three SAL for plant height using all tested accessions (Fig. 2a–c). Among these SAL, one overlapped with the *Dt1* locus [37, 38] and another overlapped with *E2*, a locus that is responsible for bloom date [39]. However, the *Dt2* locus was not detected. If an epistatic gene exhibits a significantly strong effect, it can hinder the identification of other interactive genes that exert minor effects [34, 40]. We then classified the entire population into two sub-populations (termed *Dt1* and *dt1* subgroups), based on the genotypes of the highest association site of the *Dt1* locus. A GWAS of the plant height in each of these two subgroups revealed two additional SAL in the *Dt1* subgroup, which included the *Dt2* locus (Fig. 2e and f). However, the *Dt2* locus cannot be detected in the *dt1* subgroup (Fig. 2h and i). This finding confirmed the results of previous epistasis analyses [35, 36]. In contrast, the *E2* locus was detected in both the *Dt1* and *dt1* subgroups (Fig. 2e and h), suggesting that *E2* and *Dt1* does not exert an epistatic effect. The *Dt2* locus precisely explains the phenotypic variation in plant height within the subgroup of the *Dt1* allele (Fig. 2g) compared with the *Dt1* locus alone (Fig. 2d).

To validate our method, we performed a new investigation of association loci using the previously reported methods of SNP-fixing [41] and multiple loci analysis [42]. These approaches provided the same results as our method (Additional file 5: Figure S87). We further investigated another trait, namely leaf area. The results obtained from our method, the SNP-fixing, and multiple loci analysis all demonstrated that the locus of Chr19_45150769 can interact with *Ln* to control leaf area (Additional file 5: Figure S88), confirming the reliability of our method. Following this method, for each of the primary 150 SAL, we subdivided the 809 accessions into two subgroups according to the genotypes of the locus with the lowest *P* value within the primary SAL. We also conducted permutation tests to derive the empirical thresholds and thereby to determine the secondary associated loci. We found very similar trends for the primary and secondary SAL within each trait type (Additional file 7: Table S6). Under these empirical thresholds, we identified 95 additional secondary SAL (Additional file 9: Table S8). In total, we identified 245 SAL, which included 46 SAL that overlapped with previously reported genes, 64 SAL that overlapped with reported QTLs, and 135 SAL that have not been characterized (Additional file 8: Table S7; Additional file 9: Table S8).

### Genetic architecture of fatty acid content

**Fig. 3:**
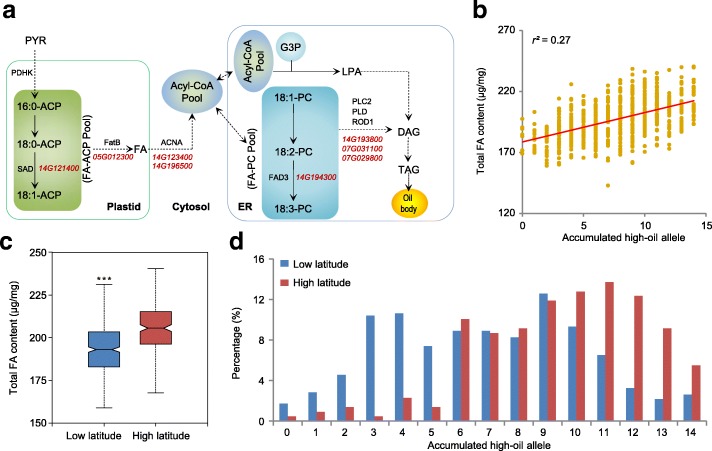
Dissection of genetic regulation of the fatty acid content in soybean. **a** Candidate genes in the lipid metabolic pathway that are responsible for the variation of fatty acid (FA) synthesis in soybean germplasm. The pathway is modified from *Arabidopsis*. The *dotted lines* represent multiple reaction steps. **b** *Plot* of the total FA content against the accumulation of high-oil-content alleles. The *x-axis* indicates the number of accumulated high-oil alleles from all candidate genes in the soybean germplasm; the *y-axis* shows the total FA content in the corresponding population. **c** Total FA content of the germplasm from low-latitude and high-latitude areas. ***P < 0.001 (one-sided Student’s *t*-test, n = 461, 219). **d** Proportion of accumulated high-oil alleles in low-latitude and high-latitude populations. *ACP* acyl carrier protein, *DAG* diacylglycerol, *G3P* glycerol-3-phosphate, *FA* fatty acid, *LPA* lysophosphatidic acid, *PC* phosphatidylcholine, *PYR* pyruvate, *TAG* triacylglycerol, *ACNA* acyl-CoA n-acyltransferase, *FAD* fatty acid desaturase, *FatB* fatty acyl-ACP thioesterase B, *PDHK* pyruvate dehydrogenase kinase, *PLC* phospholipase C, *PLD* phospholipase D, *ROD1* reduced oleate desaturation 1, *SAD* stearoyl-acyl-carrier-protein desaturase, *ER* endoplasmic reticulum

Soybean is an important oilseed crop. Our analyses dissected the genetic architecture of the fatty acid content in the soybean natural population. Fatty acid biosynthesis-related genes, such as the genes encoding fatty acyl-ACP thioesterases B (FatB), plant stearoyl-acyl-carrier protein desaturase (SAD), and fatty acid desaturase 3 (FAD3), have been reported to be responsible for fatty acid accumulation in soybean [43–45]. In this study, we found five additional fatty acid biosynthesis-related genes located within the SAL regions (Fig. 3a; Additional file 10: Table S9). The differential alleles of these eight genes exhibited significant differences in the total fatty acid (TFA) content (Additional file 5: Figure S89). In addition to the genes involved in fatty acid biosynthesis, the genes that participate in lipid biosynthesis could also affect the fatty acid accumulation [46]. We identified six lipid biosynthesis-related genes in the fatty acid-related SAL regions (Additional file 11: Table S10). The different alleles of these lipid biosynthesis-related genes also showed significant differences in the TFA content (Additional file 5: Figure S90).

We observed that the TFA content increased with the accumulation of high-fatty-acid alleles of these genes in the soybean germplasm (Fig. 3b). Further analysis demonstrated that the TFA content in high-latitude accessions was significantly higher than that of low-latitude accessions (Fig. 3c). Correspondingly, we found that high-latitude accessions accumulated more high-fatty-acid alleles than low-latitude accessions (Fig. 3d; Additional file 5: Figure S91a). The results indicated that, similar to those in maize [13], the oil synthesis-related genes in soybean functioned additively to accumulate fatty acid. A genotype investigation of the ten most widely cultivated high-oil cultivars in China illuminated that they did not possess all of the high-fatty-acid alleles in the 14 genes (Additional file 5: Figure S91b), which suggested that the pyramiding of more high-fatty-acid alleles in these lines will allow the development of a soybean variety with a higher oil content.

### Genetic network of loci associated with phenotypes

We found that the 84 traits related to growth period, architecture, color, seed development, oil content, or protein content tended to be correlated within these trait classifications (Additional file 5: Figure S92), suggesting that they might be genetically co-regulated. The plotting of the SAL across the soybean genome revealed that they were clustered according to the phylogeny relationship of traits rather than distributed randomly on the chromosomes (Additional file 5: Figure S93).

**Fig. 4:**
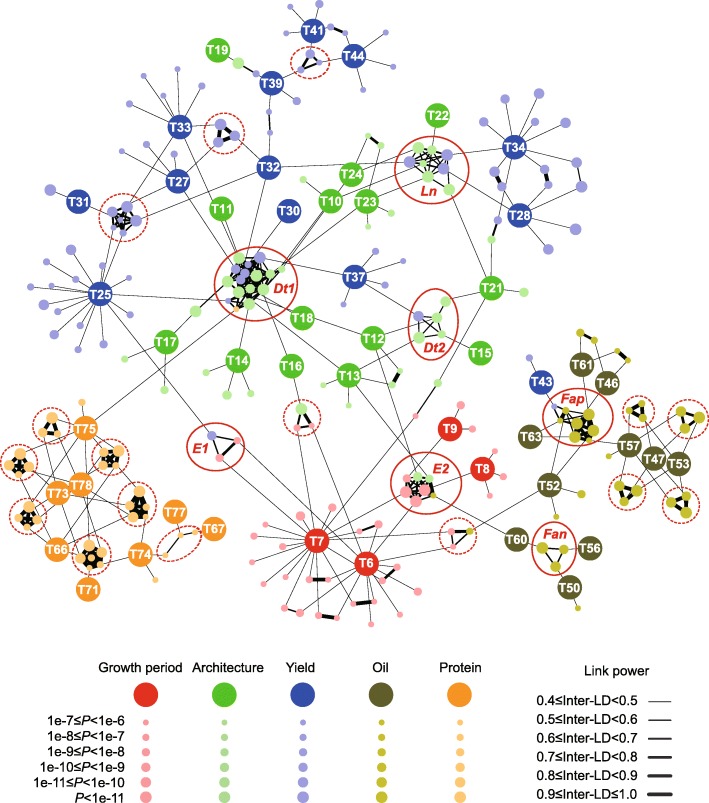
Association networks across different traits in soybean. The nodes represent traits and their responsible SAL. The edges between the SAL from different traits are linked by LD. Only the edges with an average LD ≥ 0.4 are displayed. The trait abbreviations match those in Additional file 6: Table S5. The overlapped SAL covering *Dt1*, *Dt2*, *E1*, *E2*, *Ln*, *Fan*, and *Fap* are indicated by the actual *circles*. Other linked SAL covering unknown QTL are indicated by the *dotted circles*

Pleiotropy and linkage disequilibrium (LD) play important roles in identifying correlations among phenotypes [23]. To dissect the genetic architecture of the correlations across different traits, we analyzed the association networks using a previously reported method [47] with slight modification (see “Methods”). The network analysis revealed that the SAL were connected for most of the traits (Fig. 4), with the exception of two traits related to color (Additional file 5: Figure S94). Consistent with the correlation pattern of the traits (Additional file 5: Figure S92), the SAL controlling association phenotypes, such as growth period, architecture, yield, oil biosynthesis, or protein biosynthesis prefer to cluster as more closely linked networks (Fig. 4; Additional file 12: Table S11). Additionally, we determined that a number of SAL, such as the *E2*, *E1*, *Dt1*, *Dt2*, *Ln*, *Fan*, *Fap*, and several newly identified loci, played roles as key nodes in the regulation of different traits (Fig. 4; Additional file 5: Figure S93). One noteworthy example is the *Dt1* locus. We revealed that, besides controlling plant height, the *Dt1* locus also affected other yield related traits, such as the branch density, stem pod density, stem node number, number of three-seed per pod, and total seed number (Additional file 12: Table S11), which was validated by the comparison of these traits in *Dt1* and *dt1* isogenic lines (Additional file 5: Figure S95).

**Fig. 5:**
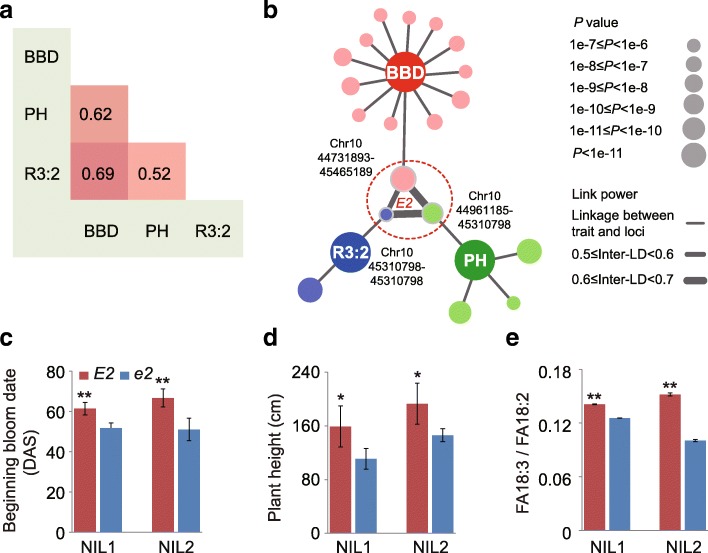
Phenotype correlations and genetic networks of associated loci. **a** The correlation among three traits: BBD, PH, and R3:2 of linolenic acid (FA18:3) to linoleic acid (FA18:2). **b** The association networks across PH, BBD, and R3:2. The genetic network presents the SAL with average LD ≥ 0.4. An overlapped SAL covering *E2* is indicated by the *dotted circle*. Phenotype data (mean ± s.d., n = 4) of different alleles of *E2* in different *E2* near isogenic lines are illustrated for BBC (**c**), PH (**d**), and R3:2 of linolenic to linoleic acid (**e**). NIL1 (PI 547553, *E1E2s-tt* vs. PI 547549, *E1e2s-tt*). NIL2 (ZK164, *E1E2E3E4* vs. ZK166, *E1e2E3E4*). *E1*, *E2*, *E3*, *E4*: loci controlling flowering ability, *s-t*: locus controlling plant height, *T*: locus controlling pubescence color. *DAS* day after sowing. *P < 0.05; **P < 0.01 (one-sided Student’s t-test)

Yield and quality are two major considerations in variety development for almost all crops. However, the loci simultaneously controlling yield-related and quality-related traits have seldom been reported [48]. In this study, we found that *E2* may exhibit pleiotropy across the traits related to yield and seed quality. Plant height (PH) and beginning bloom date (BBD) exhibited a significantly positive correlation (Fig. 5a). We found that these two traits shared a common SAL, which overlapped with the *E2* locus (Fig. 5b). This finding was consistent with previous reports that the major genes and QTLs are shared for flowering, maturity, and plant height in soybean [33, 49]. Interestingly, we found that the ratio of linolenic acid to linoleic acid (FA 18:3 to FA18:2, R3:2) also exhibited significantly positive correlations to PH and BBD (Fig. 5a), and shared *E2* with these two traits in the association network (Fig. 5b), suggesting that *E2* exhibits pleiotropy across PH, BBD, and R3:2. To verify the effects of the *E2* locus in the association network, PH, BBD, and R3:2 were compared between two pairs of *E2* and *e2* isogenic lines (PI 547553, *E1E2s-tt* vs. PI 547549, *E1e2s-tt*; ZK164, *E1E2E3E4* vs. ZK166, *E1e2E3E4*). The results showed that the values of PH, BBD, and R3:2 in the *E2* lines were significantly higher values than those in the *e2* lines (Fig. 5c–e), confirming that the *E2* locus plays an important role in regulating these three important agronomic traits in a simultaneous manner.

## Discussion

Plant breeding aims to pyramid multiple desirable traits into a single variety. However, due to trait correlations, breeders must choose to either simultaneously improve correlated traits or accept potentially undesirable effects associated with the correlation [23]. A better understanding of the genetic networks underlying these different traits helps breeders to develop effective strategies for variety development. For example, in past decades, rice functional genomics has progressed rapidly, resulting in the identification of some key genes that control both yield and grain quality [50]. The well-established genetic information has allowed scientists to propose a clear path to design the breeding of high-yield, superior-quality, hybrid super rice [4]. However, compared with rice, fundamental studies on the genetic dissection of complex traits in soybean have to take more progress to reach the same level.

Epistasis, or the interaction between genes associated with a trait, add the complexity to the genetic dissection of complex traits. The SNP-fixing [41] and multiple loci analysis [42] have been proven to be two robust methods for the identification of epistasis loci. In this study, we developed another method to identify the epistasis loci by splitting the entire population into sub-populations based on the genotypes of the highest association site and subsequently performing a second-round GWAS for each sub-population. The reliability of our results was comparable to that of the results obtained through the SNP-fixing approach and multiple loci analysis (Fig. 2; Additional file 5: Figures S87 and 88), but is an advantage in determining the epistasis relationship between different haplotypes. For instance, our analysis clearly showed that an epistatic effect was only detected between *Dt1* and *Dt2* but not between *dt1* and *Dt2*, suggesting that *dt1* is a loss/weak-of-function allele compared with *Dt1* (Fig. 2). Further validation of detailed epistatic relationship between different alleles we identified (Additional file 9: Table S8) using F2 or recombinant inbred line populations will be helpful for future functional study.

In total, we identified 245 SAL for 57 agronomical traits. Most of the reported genes that have been identified through forward genetics to control related agronomical traits, such as *Dt1*, *Dt2*, *E1*, *E2*, *Ln*, *PDH1*, *Fan*, and *Fap*, were identified. In addition, a total of 135 SAL were previously unchartered (Additional file 8: Table S7; Additional file 9: Table S8), such as the three SAL for flowering time in Chr5, Chr11, and Chr19 (Additional file 5: Figure S96). However, we indeed failed to detect the SAL for 27 traits.

We evaluated the statistical power of our analysis (Additional file 5: Figure S97, see “Methods”) and the results demonstrated that the statistical power was mainly determined by the number of quantitative trait nucleotides (QTNs) although it increased with the increase of the heritability. For instance, when a trait is controlled by small number of QTNs, such as QTN = 2, even with a heritability as low as 0.25, the statistical power reached 86%. However, for a trait that is controlled by more QTNs, such as QTN = 10, the statistical power only reached 70% even with a heritability as high as 0.75. Thereafter, we speculated that genetic complexity and lack of a major QTL are the main reasons for the inability to detect traits without SAL. For instance, we found that the seed weight exhibited a heritability of approximately 0.62 in the studied population, but no SAL for this trait was detected, which might be due to the fact that dozens of genes are responsible for the seed weight of plants [51]. Another reason might be due to the stringent threshold applied in this study. For many traits for which we did not find SAL, such as the 100-seed weight (Additional file 5: Figure S40), number of two-seed pod (Additional file 5: Figure S28), seed length (Additional file 5: Figure S42), and FA18:1 content (Additional file 5: Figure S50), clear association signals were detected, even though these signals did not pass the threshold. Taking the flowering time as an example, although a number of GWAS signals did not reach the threshold (Additional file 5: Figure S96), the homologues of the reported Arabidopsis flowering time-related genes were identified surrounding the highest-association loci of the GWAS signals. The stringent criterion might have caused false negatives, but guaranteed a lower false discovery rate (FDR) for every trait. We anticipate that the scientists working in similar areas will be quite interested in the information from this study, which will likely facilitate the identification of the responsible genes. Nevertheless, we also found that the positions of a small number of SAL might be inaccurate due to genome assembly errors (an example is shown in Additional file 5: Figure S98). Consequently, future studies should also use additional genomic approaches to confirm these SAL.

In addition to the identification of many SAL, we revealed the association networks across different traits. For example, we identified some SAL that functioned as key nodes for connecting different traits, whereas most SAL specifically controlled individual traits (Fig. 4). This information will be helpful guidance for the breeders attempting to establish a clear strategy for variety development. If the heritable covariation between different traits needs to be broken, using the specific SAL for individual traits might be more effective than the node SAL. In contrast, if the heritable covariation needs to be increased, the selection of the node SAL might be a better choice. Furthermore, the amount of genomic data provided a better understanding of the allelic variation for the genetic resource collections and will also facilitate breeders to propose an efficient path for variety improvement by design. For instance, we found that the five well cultivated high-yield varieties in the middle of China (Huang Huai Hai region) possessed less high-fatty-acid alleles for the 14 fatty acid-related SAL (Additional file 5: Figure S91b). Because the yield-related and fatty-acid-related networks were relatively independent (Fig. 4), by pyramiding all the high-fatty-acid SAL alleles into these high-yield varieties will potentially highly develop both high-yield and high-oil new varieties. Of course, a strict background selection should be performed because the favor alleles for other traits from these high-yield varieties should be maximally maintained.

## Conclusions

In summary, our work presented here provides a large dataset of loci and genes responsible for important agronomic traits in soybean, which will facilitate future functional studies and variety development.

## Methods

### Planting and phenotyping

A total of 809 soybean accessions were selected for this study. For phenotyping, all 809 accessions were planted at the Experimental Station of the Institute of Genetics and Developmental Biology, Chinese Academy of Sciences, Beijing (40°22′N and 116°23′E) during the summer seasons in 2013 and 2014. The 275 accessions collected from northern areas were planted in Mudanjiang (44°58′N and 129°60′E), Heilongjiang Province during the summer seasons in 2014 and 2015. The remaining 534 accessions collected from Huang Huai Hai and southern areas were planted in Zhoukou (33°62′N and 114°65′E), Henan Province during the summer seasons in 2014 and 2015. Normal seeds were selected and sowed in deeply ploughed fields with proper moisture content (15–20%). The seed was planted in three-row plots in a randomized complete block design with three replications for each environment. Only one accession was planted in each plot and the plots were 5 m in length with a row spacing of 0.4 m. The space between two plots was 0.4 m. After three weeks, the seedlings were manually thinned to achieve an equal density of 120,000 individuals per hectare.

We used the same phenotyping procedure and scoring standards in all six environments. In total, we characterized 84 sets of phenotypes related to yield, coloration, architecture, growth period, and seed composition with a miss rate < 10%. The identification of growth periods, including BBD, full bloom date, pod maturity date, and reproduction stage length, was based on a previous description of reproductive stages [52]. Traits related to flower and leaf were observed and measured at the full-bloom stage. Yield-related traits, such as pod number, seed number, and seed weight, were counted or measured in the laboratory after harvest. Detailed information regarding the phenotyping procedure and scoring standards is provided in Additional file 6: Table S5. For the assessment of the traits that need to be evaluated during the growing season, at least five healthy individuals from each plot were randomly selected and used for phenotyping. For the traits that need to be evaluated after harvesting, the healthy plants from the three replications of each accession were first collected and at least five individuals were randomly selected and used for phenotyping. The narrow-sense heritability was estimated by using GAPIT [53]. For the correlation analysis, we treated the binary traits as continued traits and converted the values into 0 or 1 and then did the correlation analysis with other quantitative traits.

### Oil and protein sample preparation and GC-MS analysis

After drying at 80 °C for 2 h, approximately 5 g of mature and well-rounded seeds were milled to a fine powder with an electric grinder. Solid fractions were filtered out using a 0.25-mm sieve. The powders were divided into two sub-samples and measured at the same time. Six micrograms of soybean power were used to determine the lipid content, according to a previously reported protocol with minor modifications [54]. Fatty acids were released from the total lipids and methylated by adding 0.8 mL of 1.25 M HCl-methanol and 20 μL of 5 mg/mL heptadecanoic acid (used as an internal standard) for 4 h at 50 °C. Then, 1 mL of hexane and 1.5 mL of 0.9% NaCl (v/v) were added to the cooled vial. After shaking for 5 min, 750 μL of the hexane layer was transferred to a new injection vial after centrifugation for 10 min at 3000 *g* and dried by flow nitrogen. The dried samples were re-dissolved in 500 μL of hexane for further GC-MS analysis.

For total amino acid analysis, 6 mg of soybean power was completely hydrolyzed by adding 300 μL of 6 M HCl spiked in 0.5 mg/mL L-norleucine (used as an internal standard) for 24 h at 100 °C [55]. After centrifugation for 30 min at 16,500 *g*, 50 μL of supernatant was transferred to a new 1.5 mL Eppendorf tube and dried at 100 °C. The dried samples were derivatized according to Fiehn’s protocol [56].

One microliter of the prepared sample (for both fatty acid and amino acid analysis) was injected into the Trace DSQII GC-MS system (Thermo Fisher Scientific), which was equipped with a DB-23 column (Agilent Technologies, 60 m × 0.25 mm × 0.25 μm) at a split ratio of 1:20 for fatty acid analysis and a DB-5MS column (Agilent Technologies, 30 m × 0.25 mm × 0.25 μm) at a split ratio of 1:50 for amino acid analysis. For fatty acid measurement, the oven was programmed as follows: 150 °C for 1 min, ramp to 200 °C at 4 °C/min, ramp to 220 °C at 2 °C/min, and finally ramp to 250 °C at 25 °C/min, holding 5 min with 1.1 mL/min helium as carrier gas [57, 58]. The temperatures of the injector, transfer line, and ion source were set to 250 °C, 250 °C, and 230 °C, respectively. For amino acid measurement, the oven was programmed as follows: 100 °C for 1 min, ramp to 240 °C at 10 °C/min, and finally ramp to 300 °C at 30 °C/min, holding 5 min with 1.1 mL/min helium as carrier gas. The temperatures of the injector, transfer line, and ion source were set to 250 °C, 250 °C, and 280 °C, respectively.

### Overall performances of the 809 soybean accessions across environments

The overall performances of the 809 soybean accessions were calculated as the best linear unbiased prediction (BLUP), the same method used to calculate the overall performances of 5000 maize inbred lines to eliminate environment effects [12]. The calculation was performed by using the function of “lmer” in the lme4 package. The fixed effects in the MLM included the overall mean and the effects of the planting environment. The planting environments were defined as each combination of year and location. The random effects in the MLM included the line effects, the interaction between environments and lines, and the residuals. The solutions of line effects (i.e. BLUP) were used as the overall performances of the 809 soybean accessions across environments.

### DNA preparation and sequencing

Among the 809 soybean accessions, 240 were obtained from our previous study [28] (Additional file 1: Table S1). The genomic DNA of the other 569 additional accessions was extracted from the young leaves of a single soybean plant for each accession, after three weeks of growth. DNA extraction was performed using the cetyltrimethylammonium bromide (CTAB) method [59]. The library of each accession was constructed with an insert size of approximately 500 bp, following the manufacturer’s instructions (Illumina Inc., 9885 Towne Centre Drive, San Diego, CA 92121, USA). All soybean varieties were sequenced on Illumina HiSeq 2000 sequencer and Illumina HiSeq 2500 sequencer at BerryGenomics Company (http://www.berrygenomics.com/. Beijing, China). Detailed information of the 809 accessions, including geographical distribution and sequencing depth, is provided in Additional file 1: Table S1.

### Read alignment and variation calling

The re-sequencing reads of the 809 accessions were mapped to the soybean reference genome [60] (Williams 82 assembly V2.0) at the Phytozome v11.0 website (http://www.phytozome.net/soybean) with BWA [61] (version 0.7.5a-r405) using the default parameters. We generated the BAM format of the mapping results and filtered the non-unique and unmapped reads with SAMtools [62] (version:0.1.19). The Picard package (http://broadinstitute.github.io/picard/, version: 1.87) was applied to filter the duplicated reads.

The Genome Analysis Toolkit [63] (GATK, version: 3.1-1-g07a4bf8) was applied for SNP and INDEL calling. Annotations of SNP and INDEL were performed based on gene model set v2.0 from Phytozome v11.0 using ANNOVAR [64] (version: 2015-03-22). The k-nearest neighbor-based method (http://202.127.18.228/fimg/intr.php) was then used for missing data imputation, after which the miss rate decreased from 2.1% to 0.057% and the heterozygous rate decreased from 3.4% to 0.17%. To evaluate the SNPs calling and imputation accuracy, we randomly selected ten fragments (primers information is listed in Additional file 3: Table S3) across the genome that contained 37 SNPs for additional validation. These fragments were amplified in 96 randomly selected soybean accessions and sequenced using the Sanger method. The comparisons between SNP calling and Sanger sequencing are shown in Additional file 4: Table S4.

The results showed that the accuracy rate of imputation SNP reached 99.8%. According to the genome annotation, the varieties were divided into exonic regions, splicing sites (within 2 bp of a splicing junction), 5’UTRs, 3’UTRs, intragenic regions, upstream and downstream regions (within a 1-kb region upstream/downstream from the transcription start/end site), and intergenic regions. The SNPs in coding regions were further categorized into non-synonymous SNPs (cause amino acid changes), synonymous SNPs (do not cause amino acid changes), stopgain SNPs (create a stop codon), and stoploss SNPs (eliminate a stop codon). The INDELs in coding regions were further categorized into non-frameshift (do not cause frameshift changes), frameshift (cause frameshift changes), stopgain, and stoploss INDELs.

### Population genetics analysis and GWAS

A neighbor-joining tree was constructed using the PHYLIP software [65] (version 3.68) on the basis of a distance matrix, using the whole-genome SNPs shared by all the accessions. A principal component analysis (PCA) of the population was performed via EIGENSOFT software [66] (version 4.2). The population structure was calculated using the Bayesian clustering program fastStructure [31]. LD was calculated using PLINK [67] (version: 1.90) with the parameter --ld-window-r2 0 --ld-window 99999 --ld-window-kb 1000. Only SNPs with MAF ≥ 0.05 and missing rate < 0.1 in the population were used in the GWAS. An association analysis was performed using the EMMAX (beta version) [68] software package. The matrix of pairwise genetic distances, which were derived from the simple matching coefficients, as the variance-covariance matrix of the random effects, was also calculated by EMMAX.

### Determination of genome-wide threshold

We randomly shuffled observed real phenotypes to break the connections between these phenotypes and their corresponding genotypes. Then, we applied the GWAS on the permuted phenotypes by using the same model that was used for real observed phenotypes. The most significant *P* value across the whole genome was recorded. This random process was repeated 1000 times. The distribution of the most significant *P* values across the 1000 replicates was used to determine the threshold, which was the *P* value corresponding to a 5% chance of a type I error.

Ideally, each trait should have its own threshold. To derive robust thresholds, we grouped the 84 traits into four types based on their phenotypic distribution. We found the thresholds were very similar within each of the types we defined as follows:

1. Binary traits: examples include color (purple vs. white)
2. Quantitative traits with normal distribution
3. Quantitative traits with skewed distribution
4. Binary-like quantitative traits: examples include four-seed pod number and ratio with extremely skewed frequency distributions

We tested multiple traits in each category and randomly selected one trait out of each category to illustrate the empirical thresholds (Additional file 7: Table S6). The first three types of traits had very similar thresholds (negative log_10_ *P* values = 6.5–6.7). We used the most stringent threshold (6.7) as the criterion for these three types of traits. Although this criterion may have caused false negatives, it guaranteed that the type I error was below 5% for every trait. The last type of traits had much more stringent criteria. For example, the four-seed per pod ratio had threshold of 8.3 (negative log_10_ *P* value). We used this threshold for all binary-like quantitative traits.

### Identification of additional minor-effect loci

To identify minor-effect loci by eliminating the effect of epistasis or interactions between genes, additional GWAS were performed. We first divided the 809 accessions into two subgroups according to the genotype of the SNP with the lowest *P* value out of all SAL across the whole genome. Next, association analysis was performed only if the subgroup consisted of more than 100 accessions. With the same method, the significant thresholds of minor loci were determined (Additional file 7: Table S6). Negative log *P* value of 8.4 was used as threshold for all binary-like quantitative traits. We used the more stringent threshold (6.6) as the criterion for the traits in other two categories. The significant associated loci and not having been identified before grouping were considered as new identified association signals.

### Assessment of statistical examination

Using the genotype data of 809 soybean accessions, a set of SNPs (2, 5, and 10) were randomly selected as causal loci for the simulated traits using the method described previously [69]. Three levels of heritability (*h* ^2^ = 0.25, 0.5, and 0.75) were evaluated for examination of statistical power in all settings of causal loci. For each combination of heritability and number of causal loci, a total of 1000 replicates were conducted for the simulation of phenotypes and association tests. In each of GWAS, the threshold was set as 2 × 10^–7^, the cutoff from permutation tests on real traits with normal distribution.

Statistical power and false positive rate (FDR) were evaluated on the intervals around the loci above the threshold. An interval was defined as the consecutive region with SNPs in LD (above 0.6) around the associated locus. Statistical power was calculated as the proportion of intervals containing causal loci over the total number of causal loci weighted by variance they explained. FDR was calculated as the proportion of the intervals without causal locus over the total number of intervals with a SNP above the threshold. The averages and standard error of statistical power and FDR over the 1000 replicates were reported.

### Construction of association networks

The association networks were constructed using the software Cytoscape [70] (Version: 3.2.1), with traits and their corresponding SAL as nodes, and the link between trait and SAL, SAL and SAL (average *r* ^2^ ≥ 0.4) as edges. The effective score for each SAL was represented by the lowest *P* value. The link between each two SAL was represented by their average LD (Inter-LD). Inter-LD was calculated as follows:

$$ Inter- LD=1/2\times \left(\frac{LD\left( SAL1, SAL2\right)}{PmaxLD(SAL1)},+,\frac{LD\left( SAL1, SAL2\right)}{PmaxLD(SAL2)}\right), $$

where LD (SAL_1_, SAL_2_) equals the mean of pairwise LD value (*r* ^2^) between all the SNPs from SAL_1_ to all the SNPs from SAL_2_; PmaxLD (SAL_1_) equals the largest possible LD value within the SAL_1_ region, obtained by calculating the mean *r* ^2^ of each SNP to all SNPs from the SAL_1_ region, and then choosing the maximum mean of the LD value to represent this region’s PmaxLD; and PmaxLD (SAL_2_) equals the largest possible LD value within the SAL_2_ region, obtained by calculating the mean *r* ^2^ of each SNP to all SNPs from SAL_2_ region, and then choosing the maximum mean of the LD value to represent this region’s PmaxLD. Pairwise *r* ^2^ values were calculated between all significant SNPs using PLINK [67].

## Additional files

Additional file 1: Table S1. Summary of the 809 soybean accessions. (XLSX 55 kb)

Additional file 2: Table S2. Whole-genome SNP and INDEL distribution. (PDF 133 kb)

Additional file 3: Table S3. Primers used for SNP validation. (XLSX 11 kb)

Additional file 4: Table S4. The SNPs getting from before imputation, after imputation, and Sanger sequencing validation. (XLSX 55 kb)

Additional file 5: Figures S1-S98. with legends. GWAS results of individual traits and the correlation of different traits. (PDF 21,979 kb) (PDF 21766 kb)

Additional file 6: Table S5. Information of 84 phenotype traits. (XLSX 15 kb)

Additional file 7: Table S6. Permuted significant thresholds of representative traits. (XLSX 10 kb)

Additional file 8: Table S7. Genome-wide association signals of 57 agronomic traits. (XLSX 30 kb)

Additional file 9: Table S8. Genome-wide association signals of additional minor-effect loci. (XLSX 23 kb)

Additional file 10: Table S9. Candidate causal genes in the fatty acid biosynthesis pathway. (XLSX 10 kb)

Additional file 11: Table S10. Candidate causal genes in the lipid biosynthesis pathway. (XLSX 11 kb)

Additional file 12: Table S11. Link loci across 51 traits. (XLSX 21 kb)
