# Supplementary material for: Genome-wide association studies dissect the genetic networks underlying agronomical traits in soybean
Source: Genome Biol. 2017 Aug 24;18:161. doi: 10.1186/s13059-017-1289-9 (PMC5571659; doi:10.1186/s13059-017-1289-9)
Supplement: Supplementary file 2 — Whole-genome SNP and INDEL distribution. (PDF 133 kb) [file 13059_2017_1289_MOESM2_ESM.pdf]

Supplementary Table S2 Whole-genome SNP and INDEL distribution

Type	SUM	Exonic	Intergenic	Intronic	Splicing	Upstream	Downstream	5'UTR	3'UTR
SNP	10,415,168	358,426	7,715,773	1,013,011	3,508	585,107	545,852	95,322	140,837
INDEL	1,033,071	15,420	651,744	148,782	505	94,290	87,663	17,552	25,032

Type	Nonsynonymous	Synonymous	Stopgain	Stoploss
SNP	210,686	140,335	6,656	749

Type	Frameshift	Nonframeshift	Stopgain	Stoploss
INDEL	11,203	3,830	342	45
