# Supplementary material for: Genome-wide association studies dissect the genetic networks underlying agronomical traits in soybean
Source: Genome Biol. 2017 Aug 24;18:161. doi: 10.1186/s13059-017-1289-9 (PMC5571659; doi:10.1186/s13059-017-1289-9)
Supplement: Supplementary file 5 — with legends. GWAS results of individual traits and the correlation of different traits. (PDF 21,979 kb) (PDF 21766 kb) [file 13059_2017_1289_MOESM5_ESM.pdf]

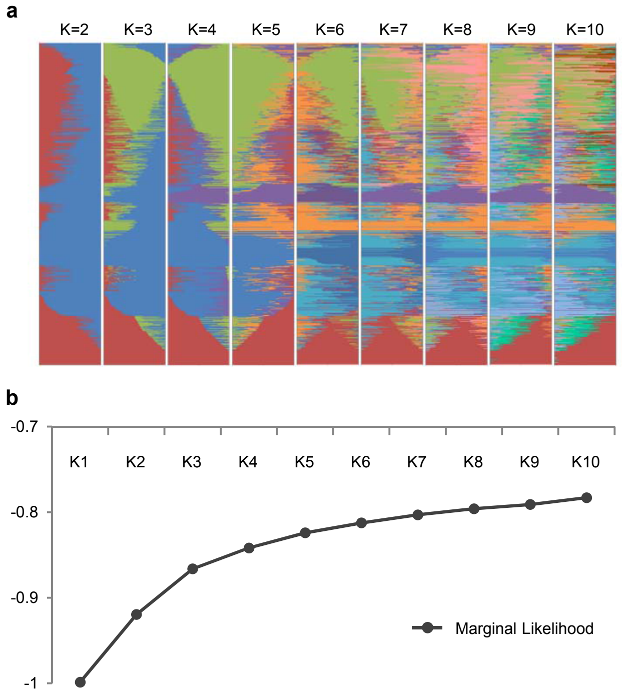

Supplementary Figure 1 Population structure of the diversity panel. (**a**) Genetic structure analysis using the program fastStructure. Each color represents one ancestral population. Each accession is represented by a horizontal bar, and the length of each colored segment in each horizontal bar represents the proportion contributed by ancestral population. (**b**) K = 4 is chosen as the optimal number of subpopulations.

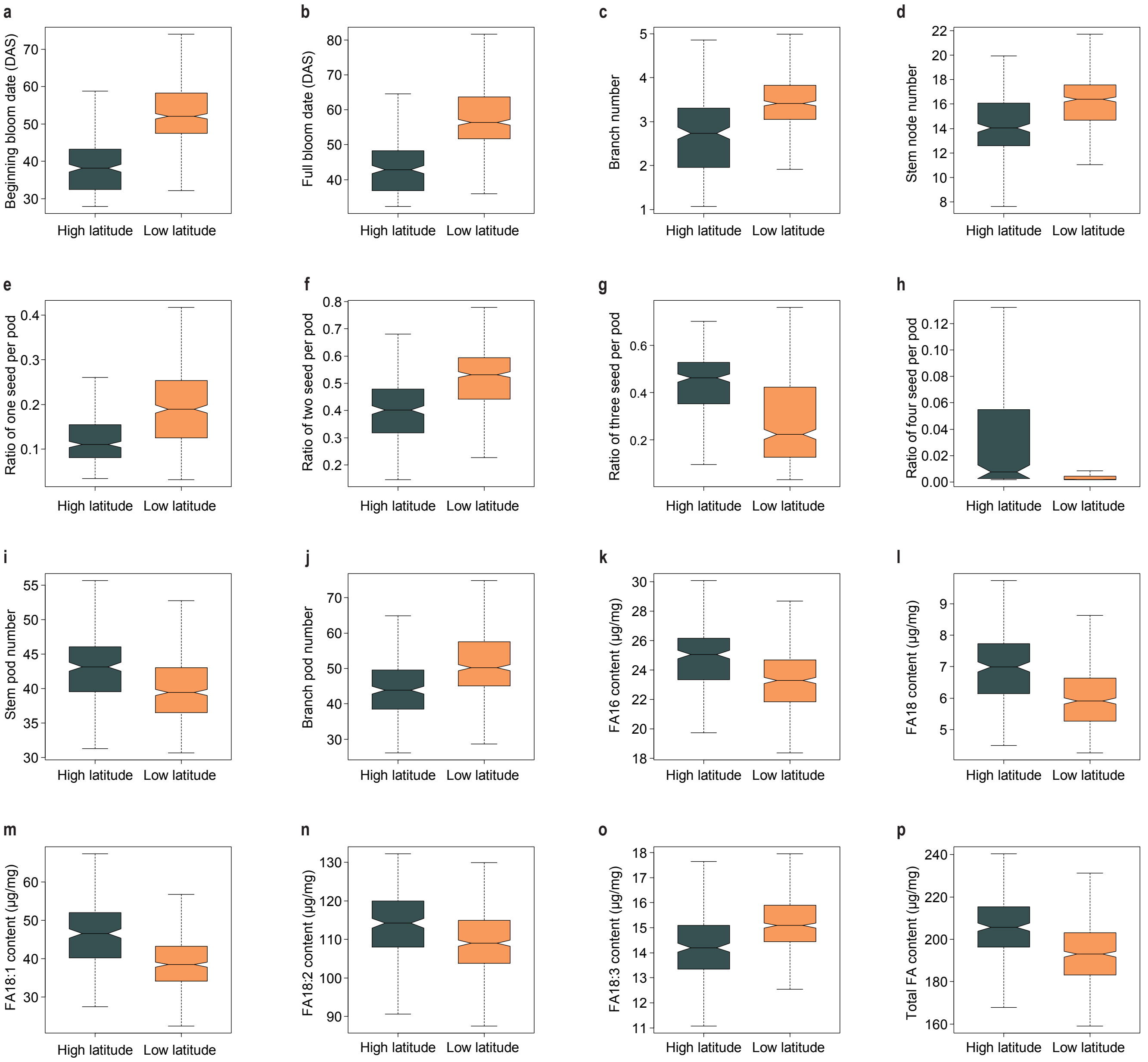

Supplementary Figure 2 Phenotyping differences between high latitude and low latitude soybean accessions. (**a**) Beginning bloom date (*n* = 257, 513). (**b**) Full bloom date (*n* = 257, 513). (**c**) Branch number (*n* = 254, 509). (**d**) Stem node number (*n* = 254, 509)). (**e**) Ratio of one seed per pod (*n* = 253, 501). (**f**) Ratio of two seed per pod (*n* = 253, 501). (**g**) Ratio of three seed per pod (*n* = 253, 501). (**h**) Ratio of four seed per pod (*n* = 253, 501). (**i**) Pod number on the stem (*n* = 254, 509). (**j**) Pod number on the branch (*n* = 254, 509). (**k**) Fatty acid 16 content (*n* = 236, 486). (**l**) Fatty acid 18 content (*n* = 236, 486). (**m**) Fatty acid 18:1 content (*n* = 236, 486). (**n**) Fatty acid 18:2 content (*n* = 236, 486). (**o**) Fatty acid 18:3 content (*n* = 236, 486). (**p**) Total fatty acid content (*n* = 236, 486). DAS, day after sowing. Significant difference is detected in each trait at $P < 0.001$ (one-sided Student's *t*-test).

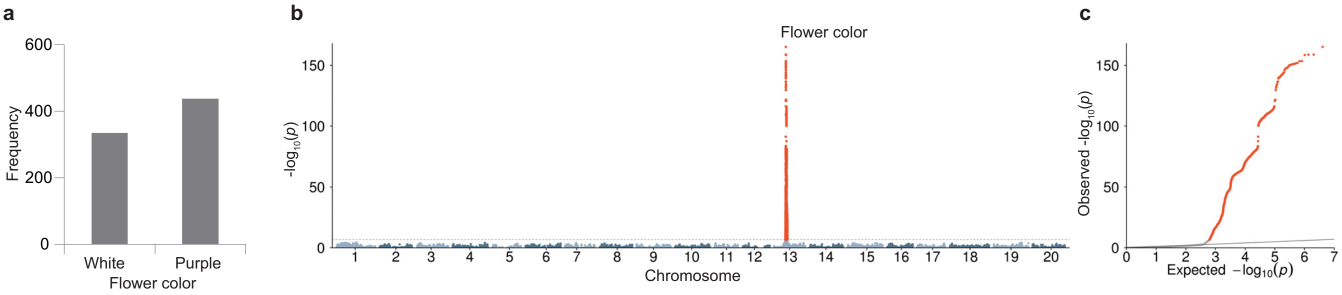

Supplementary Figure 3 GWAS of flower color using EMMAX. (**a**) Frequency distribution of flower color. (**b**) Manhattan plots for flower color. Negative $\log_{10}$ *P*-values from a genome-wide scan are plotted against SNP positions of 20 chromosomes. (**c**) Quantile-quantile plot for flower color. The horizontal dash line indicates the significant threshold ($2{\times}10^{-7}$). Trait-associated SNPs above the significant threshold are colored in red.

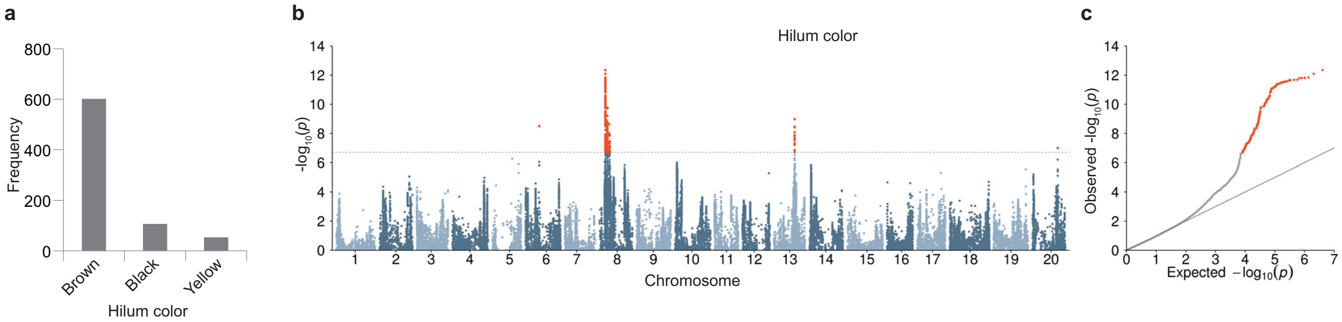

Supplementary Figure 4 GWAS of hilum color using EMMAX. (**a**) Frequency distribution of hilum color. (**b**) Manhattan plots for hilum color. Negative $\log_{10}$ *P*-values from a genome-wide scan are plotted against SNP positions of 20 chromosomes. (**c**) Quantile-quantile plot for hilum color. The horizontal dash line indicates the significant threshold (2×10^{-7}). Trait-associated SNPs above the significant threshold are colored in red.

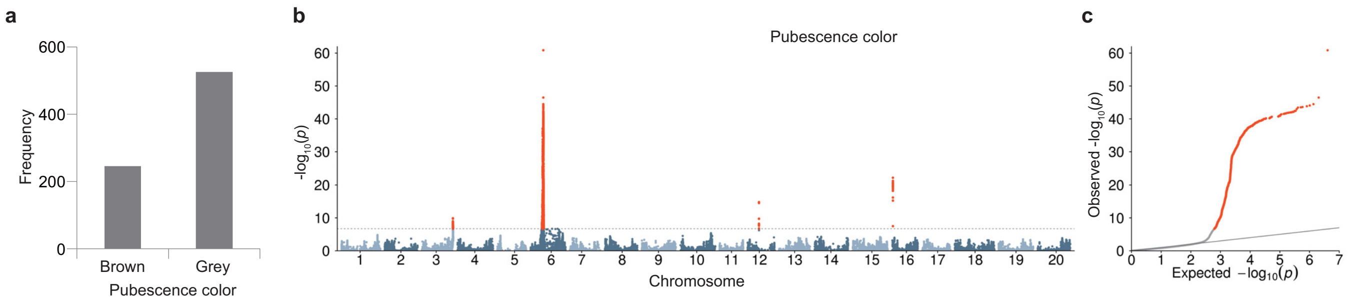

Supplementary Figure 5 GWAS of pubescence color using EMMAX. (**a**) Frequency distribution of pubescence color. (**b**) Manhattan plots for pubescence color. Negative $\log_{10}$ *P*-values from a genome-wide scan are plotted against SNP positions of 20 chromosomes. (**c**) Quantile-quantile plot for pubescence color. The horizontal dash line indicates the significant threshold (2×10^{-7}). Trait-associated SNPs above the significant threshold are colored in red.

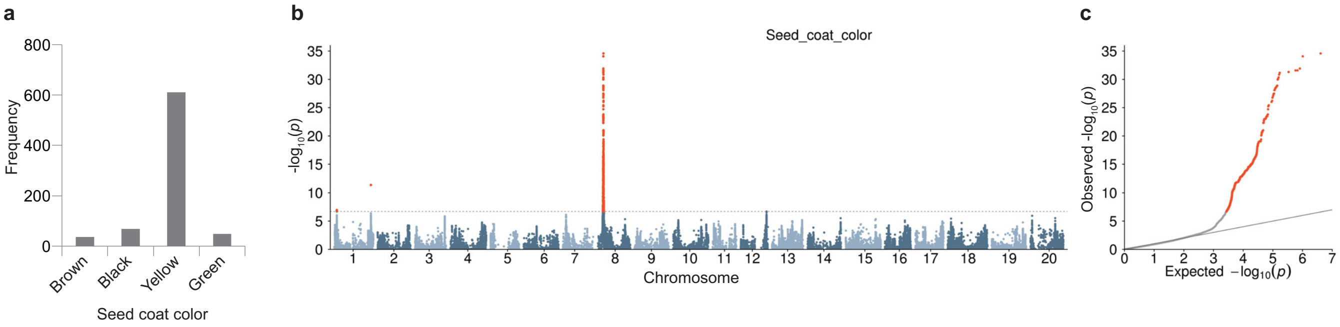

Supplementary Figure 6 GWAS of seed coat color using EMMAX. (**a**) Frequency distribution of seed coat color. (**b**) Manhattan plots for seed coat color. Negative $\log_{10}$ *P*-values from a genome-wide scan are plotted against SNP positions of 20 chromosomes. (**c**) Quantile-quantile plot for seed coat color. The horizontal dash line indicates the significant threshold (2×10^{-7}). Trait-associated SNPs above the significant threshold are colored in red.

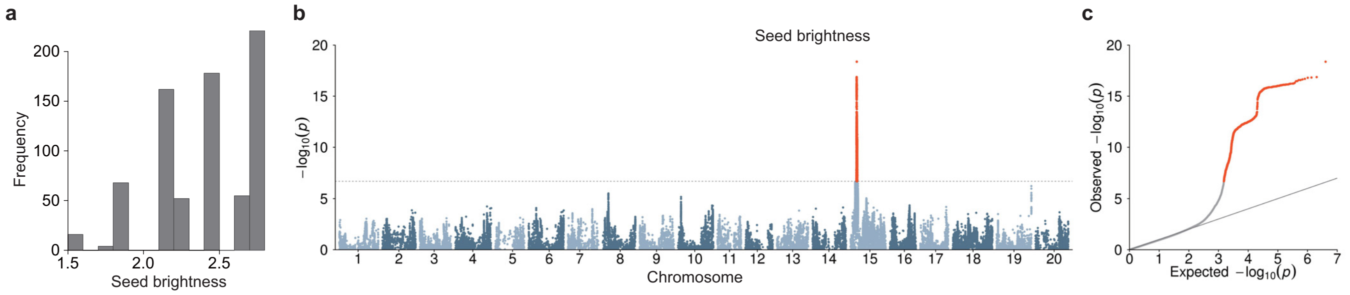

Supplementary Figure 7 GWAS of seed brightness using EMMAX. (**a**) Frequency distribution of seed brightness. (**b**) Manhattan plots for seed brightness. Negative $\log_{10}$ *P*-values from a genome-wide scan are plotted against SNP positions of 20 chromosomes. (**c**) Quantile-quantile plot for seed brightness. The horizontal dash line indicates the significant threshold (2×10^{-7}). Trait-associated SNPs above the significant threshold are colored in red.

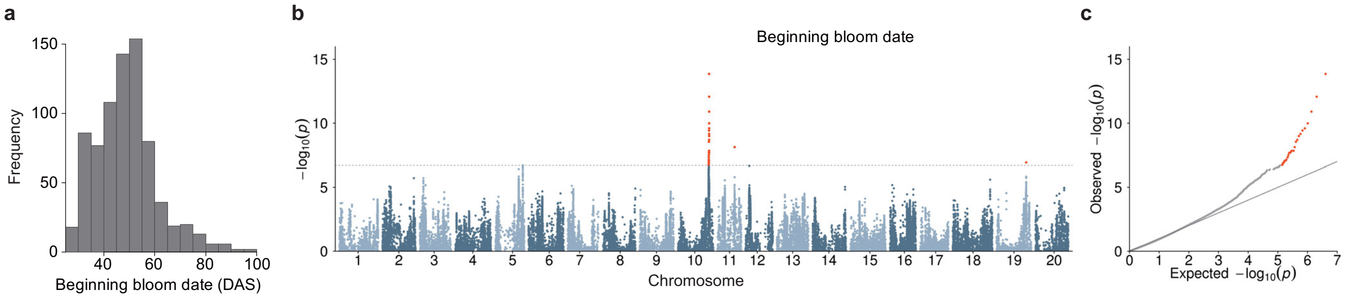

Supplementary Figure 8 GWAS of beginning bloom date using EMMAX. (**a**) Frequency distribution of beginning bloom date. (**b**) Manhattan plots for beginning bloom date. Negative $\log_{10}$ *P*-values from a genome-wide scan are plotted against SNP positions of 20 chromosomes. (**c**) Quantile-quantile plot for beginning bloom date. The horizontal dash line indicates the significant threshold (2×10^{-7}). Trait-associated SNPs above the significant threshold are colored in red. DAS, day after sowing.

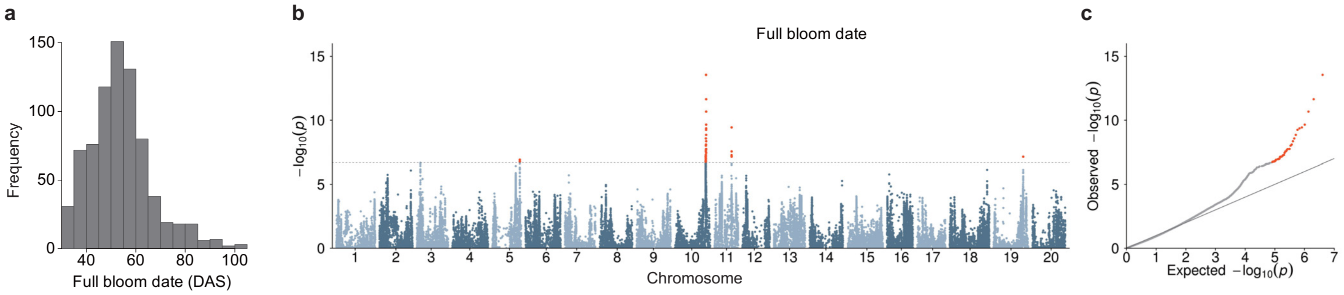

Supplementary Figure 9 GWAS of full bloom date using EMMAX. (**a**) Frequency distribution of full bloom date. (**b**) Manhattan plots for full bloom date. Negative $\log_{10}$ *P*-values from a genome-wide scan are plotted against SNP positions of 20 chromosomes. (**c**) Quantile-quantile plot for full bloom date. The horizontal dash line indicates the significant threshold (2×10^{-7}). Trait-associated SNPs above the significant threshold are colored in red. DAS, day after sowing.

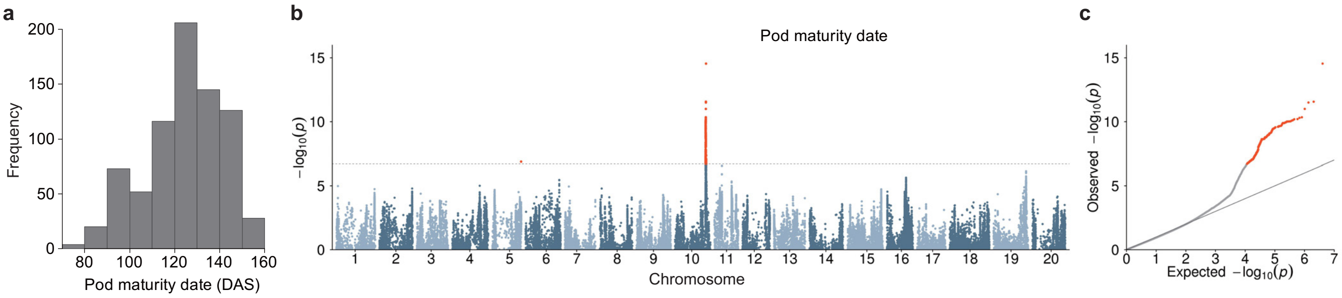

Supplementary Figure 10 GWAS of pod maturity date using EMMAX. (**a**) Frequency distribution of pod maturity date. (**b**) Manhattan plots for pod maturity date. Negative $\log_{10}$ *P*-values from a genome-wide scan are plotted against SNP positions of 20 chromosomes. (**c**) Quantile-quantile plot for pod maturity date. The horizontal dash line indicates the significant threshold (2×10^{-7}). Trait-associated SNPs above the significant threshold are colored in red. DAS, day after sowing.

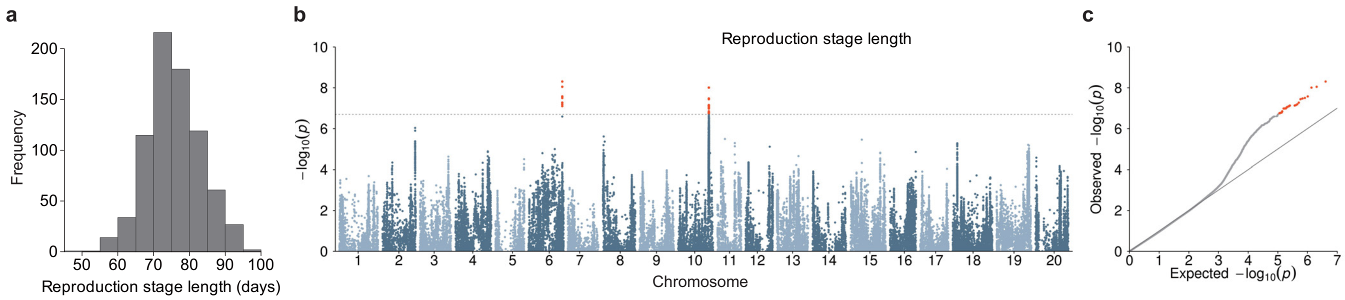

Supplementary Figure 11 GWAS of reproduction stage length using EMMAX. (**a**) Frequency distribution of reproduction stage length. (**b**) Manhattan plots for reproduction stage length. Negative $\log_{10}$ *P*-values from a genome-wide scan are plotted against SNP positions of 20 chromosomes. (**c**) Quantile-quantile plot for reproduction stage length. The horizontal dash line indicates the significant threshold (2×10^{-7}). Trait-associated SNPs above the significant threshold are colored in red.

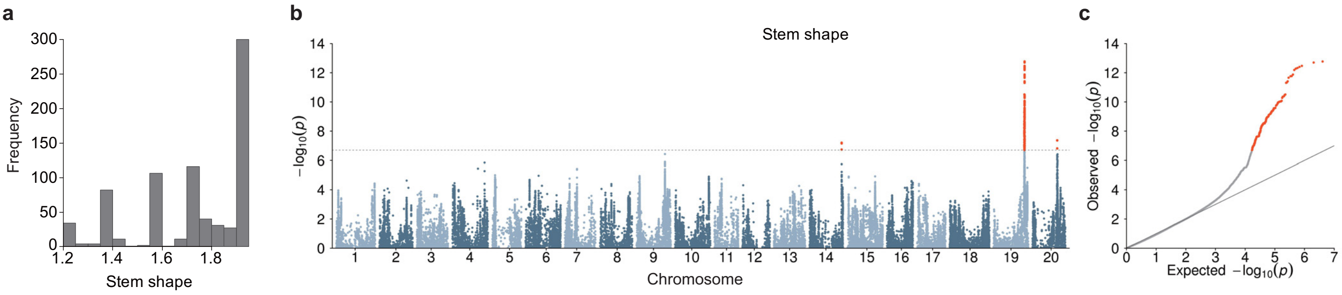

Supplementary Figure 12 GWAS of stem shape using EMMAX. (**a**) Frequency distribution of stem shape. (**b**) Manhattan plots for stem shape. Negative $\log_{10}$ *P*-values from a genome-wide scan are plotted against SNP positions of 20 chromosomes. (**c**) Quantile-quantile plot for stem shape. The horizontal dash line indicates the significant threshold (2×10^{-7}). Trait-associated SNPs above the significant threshold are colored in red.

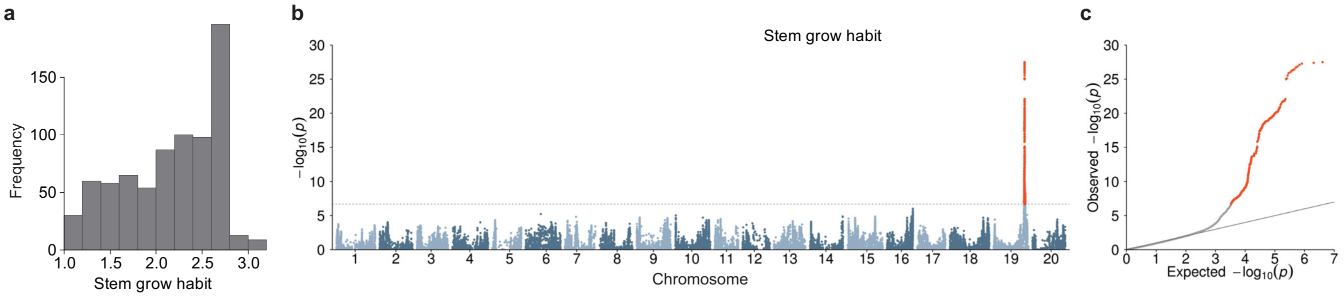

Supplementary Figure 13 GWAS of stem grow habit using EMMAX. (**a**) Frequency distribution of stem grow habit. (**b**) Manhattan plots for stem grow habit. Negative $\log_{10}$ *P*-values from a genome-wide scan are plotted against SNP positions of 20 chromosomes. (**c**) Quantile-quantile plot for stem grow habit. The horizontal dash line indicates the significant threshold (2×10^{-7}). Trait-associated SNPs above the significant threshold are colored in red.

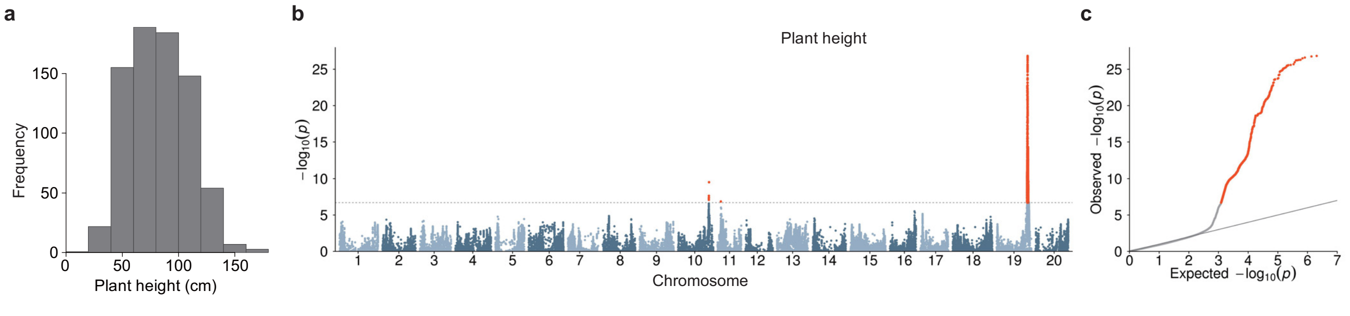

Supplementary Figure 14 GWAS of plant height using EMMAX. (**a**) Frequency distribution of plant height. (**b**) Manhattan plots for plant height. Negative $\log_{10}$ *P*-values from a genome-wide scan are plotted against SNP positions of 20 chromosomes. (**c**) Quantile-quantile plot for plant height. The horizontal dash line indicates the significant threshold ($2{\times}10^{-7}$). Trait-associated SNPs above the significant threshold are colored in red.

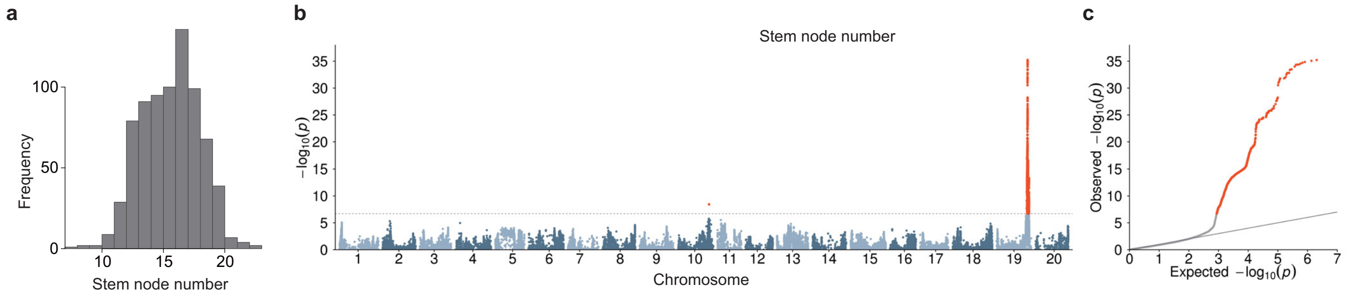

Supplementary Figure 15 GWAS of stem node number using EMMAX. (**a**) Frequency distribution of stem node number. (**b**) Manhattan plots for stem node number. Negative $\log_{10}$ *P*-values from a genome-wide scan are plotted against SNP positions of 20 chromosomes. (**c**) Quantile-quantile plot for stem node number. The horizontal dash line indicates the significant threshold (2×10^{-7}). Trait-associated SNPs above the significant threshold are colored in red.

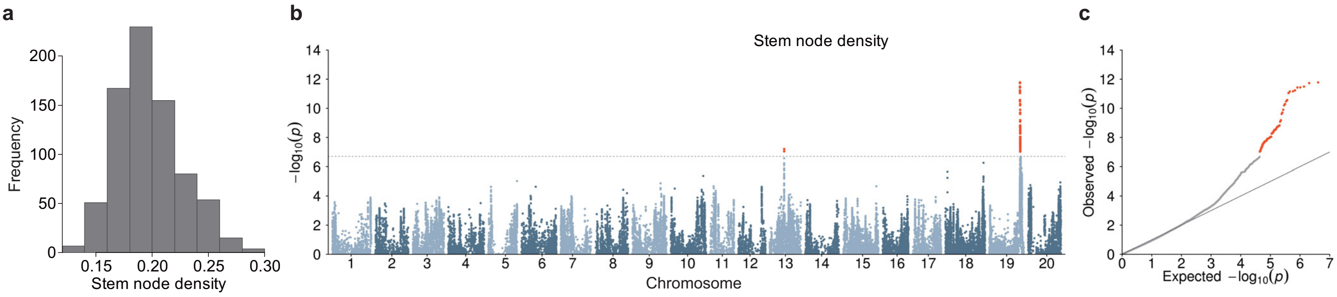

Supplementary Figure 16 GWAS of stem node density using EMMAX. (**a**) Frequency distribution of stem node density. (**b**) Manhattan plots for stem node density. Negative $\log_{10}$ *P*-values from a genome-wide scan are plotted against SNP positions of 20 chromosomes. (**c**) Quantile-quantile plot for stem node density. The horizontal dash line indicates the significant threshold ($2×10^{-7}$). Trait-associated SNPs above the significant threshold are colored in red.

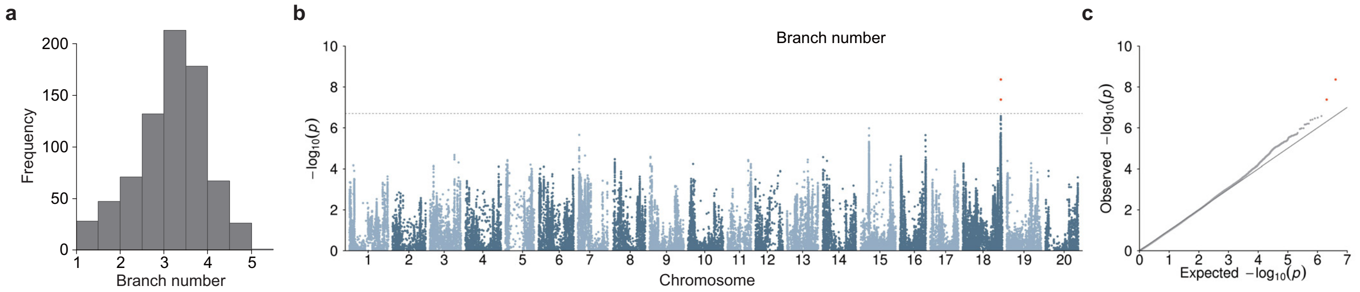

Supplementary Figure 17 GWAS of branch number using EMMAX. (**a**) Frequency distribution of branch number. (**b**) Manhattan plots for branch number. Negative $\log_{10}$ *P*-values from a genome-wide scan are plotted against SNP positions of 20 chromosomes. (**c**) Quantile-quantile plot for branch number. The horizontal dash line indicates the significant threshold (2×10^{-7}). Trait-associated SNPs above the significant threshold are colored in red.

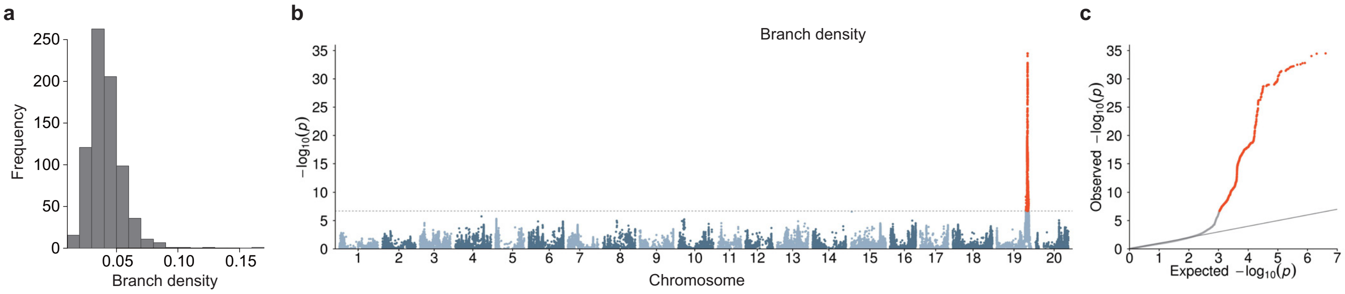

Supplementary Figure 18 GWAS of branch density using EMMAX. (**a**) Frequency distribution of branch density. (**b**) Manhattan plots for branch density. Negative $\log_{10}$ *P*-values from a genome-wide scan are plotted against SNP positions of 20 chromosomes. (**c**) Quantile-quantile plot for branch density. The horizontal dash line indicates the significant threshold (2×10^{-7}). Trait-associated SNPs above the significant threshold are colored in red.

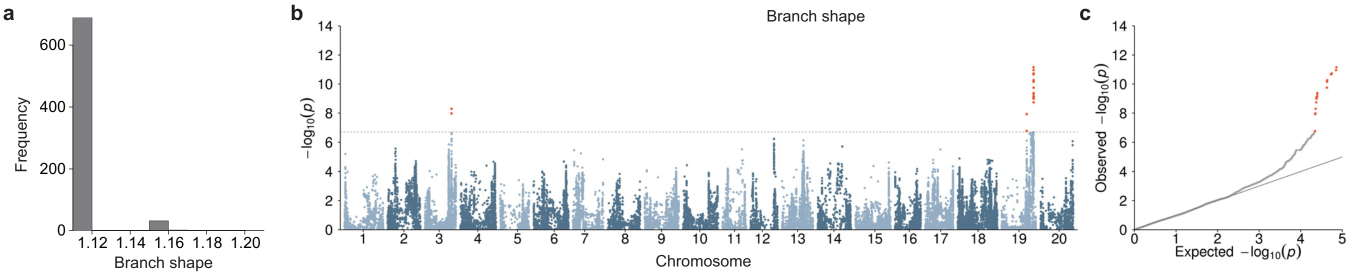

Supplementary Figure 19 GWAS of branch shape using EMMAX. (**a**) Frequency distribution of branch shape. (**b**) Manhattan plots for branch shape. Negative $\log_{10}$ *P*-values from a genome-wide scan are plotted against SNP positions of 20 chromosomes. (**c**) Quantile-quantile plot for branch shape. The horizontal dash line indicates the significant threshold ($2×10^{-7}$). Trait-associated SNPs above the significant threshold are colored in red.

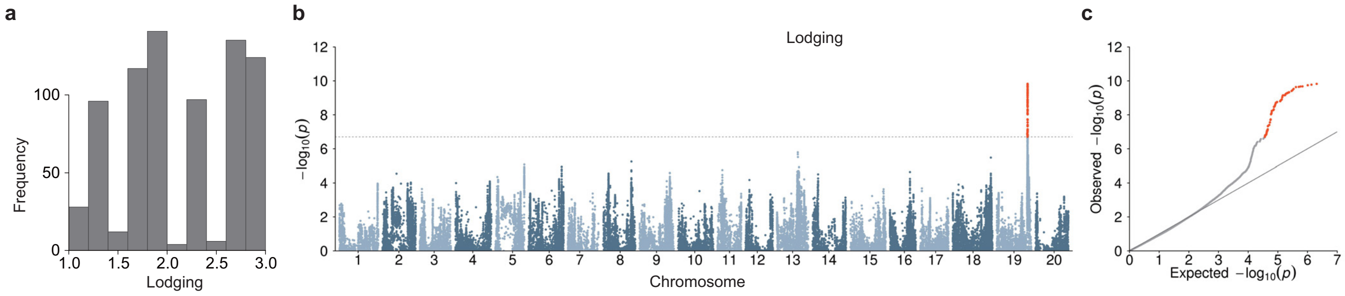

Supplementary Figure 20 GWAS of lodging using EMMAX. (**a**) Frequency distribution of lodging. (**b**) Manhattan plots for lodging. Negative $\log_{10}$ *P*-values from a genome-wide scan are plotted against SNP positions of 20 chromosomes. (**c**) Quantile-quantile plot for lodging. The horizontal dash line indicates the significant threshold (2×10^{-7}). Trait-associated SNPs above the significant threshold are colored in red.

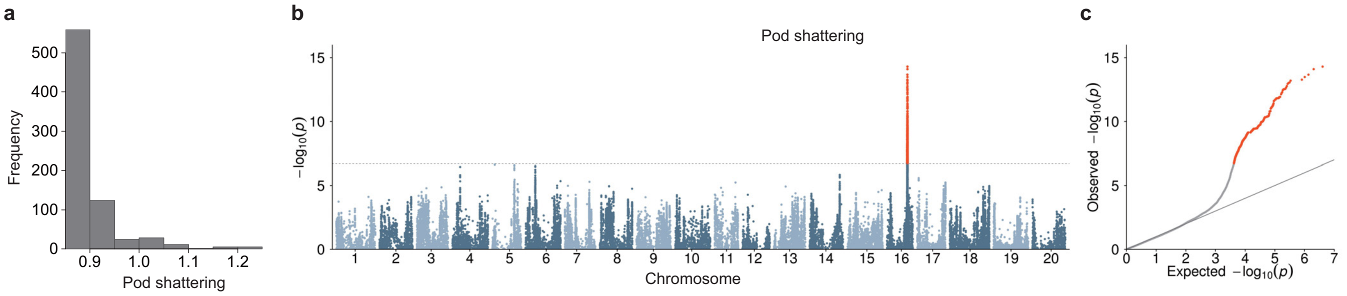

Supplementary Figure 21 GWAS of pod shattering using EMMAX. (**a**) Frequency distribution of pod shattering. (**b**) Manhattan plots for pod shattering. Negative $\log_{10}$ *P*-values from a genome-wide scan are plotted against SNP positions of 20 chromosomes. (**c**) Quantile-quantile plot for pod shattering. The horizontal dash line indicates the significant threshold (2×10^{-7}). Trait-associated SNPs above the significant threshold are colored in red.

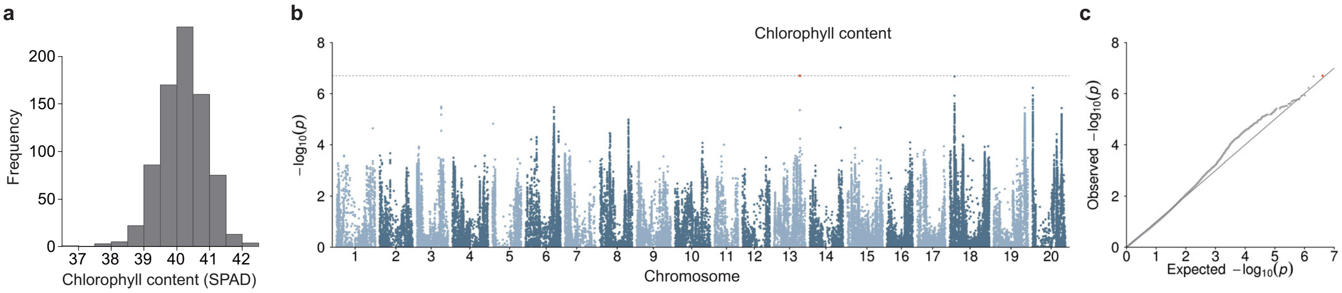

Supplementary Figure 22 GWAS of chlorophyll content using EMMAX. (**a**) Frequency distribution of chlorophyll content. (**b**) Manhattan plots for chlorophyll content. Negative $\log_{10}$ *P*-values from a genome-wide scan are plotted against SNP positions of 20 chromosomes. (**c**) Quantile-quantile plot for chlorophyll content. The horizontal dash line indicates the significant threshold (2×10^{-7}). Trait-associated SNPs above the significant threshold are colored in red. SPAD, soil plant analysis development.

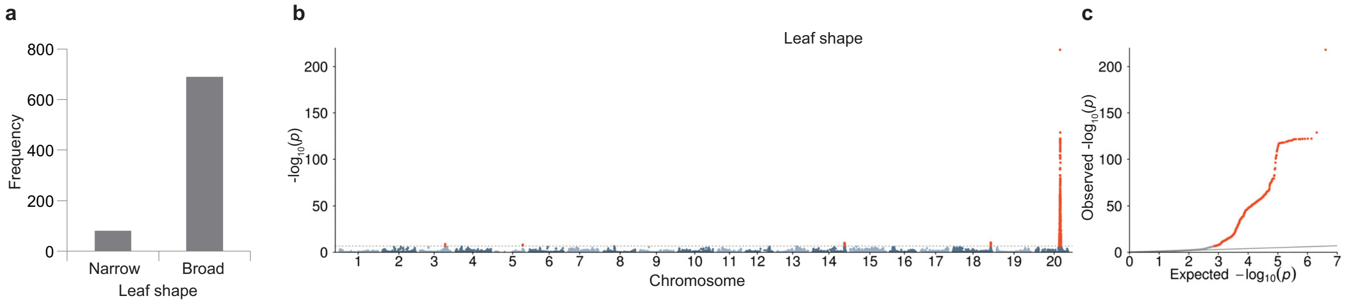

Supplementary Figure 23 GWAS of leaf shape using EMMAX. (**a**) Frequency distribution of leaf shape. (**b**) Manhattan plots for leaf shape. Negative $\log_{10}$ *P*-values from a genome-wide scan are plotted against SNP positions of 20 chromosomes. (**c**) Quantile-quantile plot for leaf shape. The horizontal dash line indicates the significant threshold (2×10^{-7}). Trait-associated SNPs above the significant threshold are colored in red.

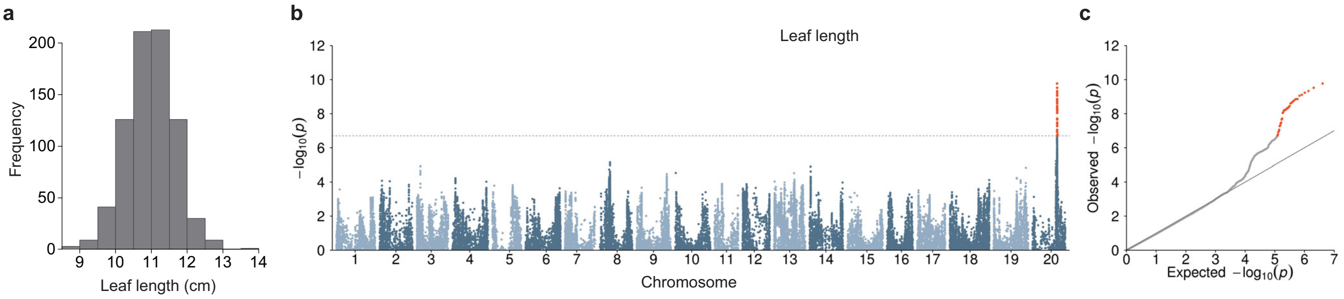

Supplementary Figure 24 GWAS of leaf length using EMMAX. (**a**) Frequency distribution of leaf length. (**b**) Manhattan plots for leaf length. Negative $\log_{10}$ *P*-values from a genome-wide scan are plotted against SNP positions of 20 chromosomes. (**c**) Quantile-quantile plot for leaf length. The horizontal dash line indicates the significant threshold (2×10^{-7}). Trait-associated SNPs above the significant threshold are colored in red.

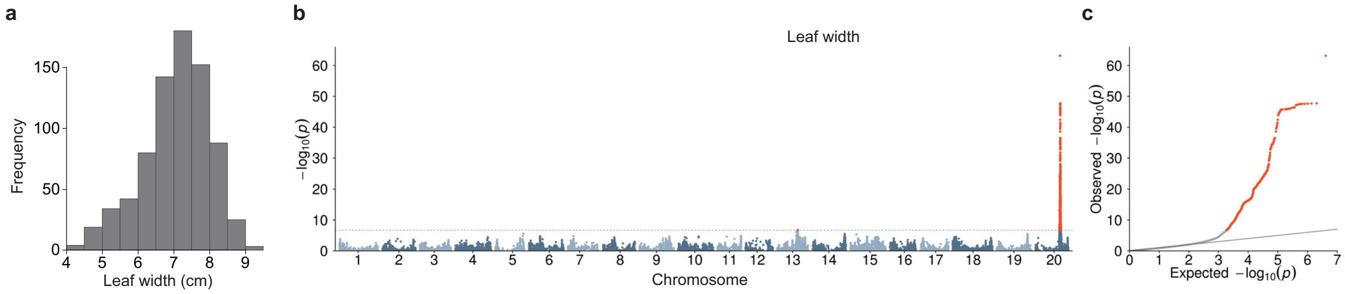

Supplementary Figure 25 GWAS of leaf width using EMMAX. (**a**) Frequency distribution of leaf width. (**b**) Manhattan plots for leaf width. Negative $\log_{10}$ *P*-values from a genome-wide scan are plotted against SNP positions of 20 chromosomes. (**c**) Quantile-quantile plot for leaf width. The horizontal dash line indicates the significant threshold (2×10^{-7}). Trait-associated SNPs above the significant threshold are colored in red.

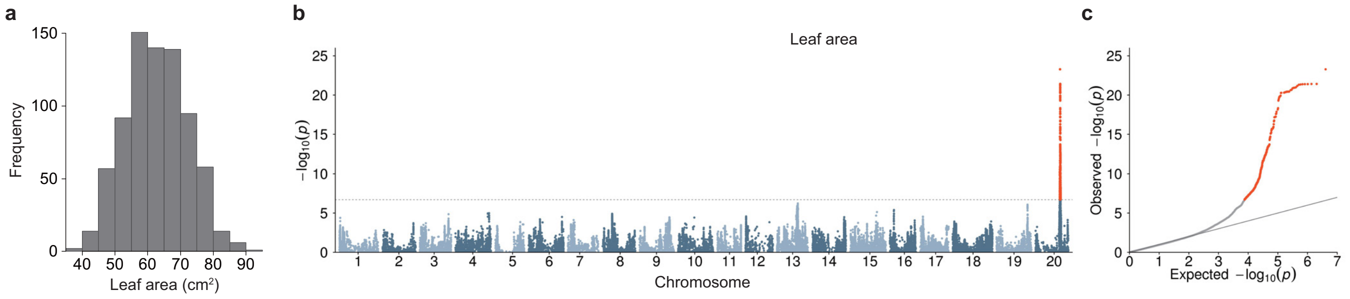

Supplementary Figure 26 GWAS of leaf area using EMMAX. (**a**) Frequency distribution of leaf area. (**b**) Manhattan plots for leaf area. Negative $\log_{10}$ *P*-values from a genome-wide scan are plotted against SNP positions of 20 chromosomes. (**c**) Quantile-quantile plot for leaf area. The horizontal dash line indicates the significant threshold (2×10^{-7}). Trait-associated SNPs above the significant threshold are colored in red.

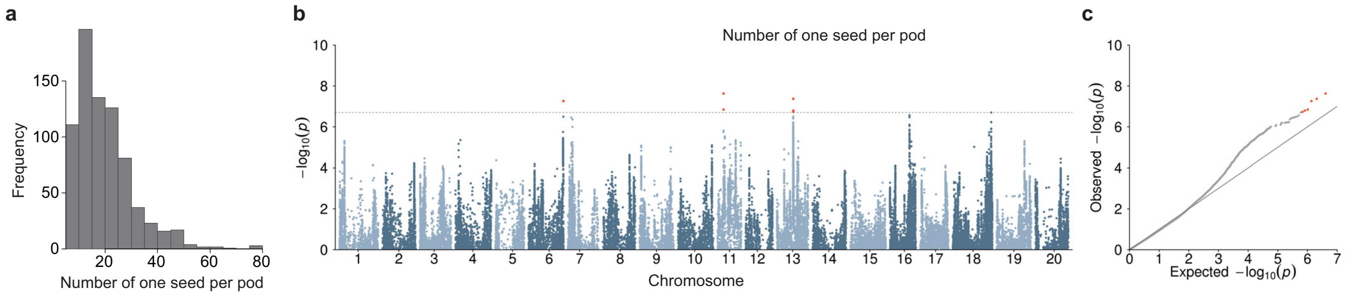

Supplementary Figure 27 GWAS of number of one seed per pod using EMMAX. (**a**) Frequency distribution of number of one seed per pod. (**b**) Manhattan plots for number of one seed per pod. Negative $\log_{10}$ *P*-values from a genome-wide scan are plotted against SNP positions of 20 chromosomes. (**c**) Quantile-quantile plot for number of one seed per pod. The horizontal dash line indicates the significant threshold (2×10^{-7}). Trait-associated SNPs above the significant threshold are colored in red.

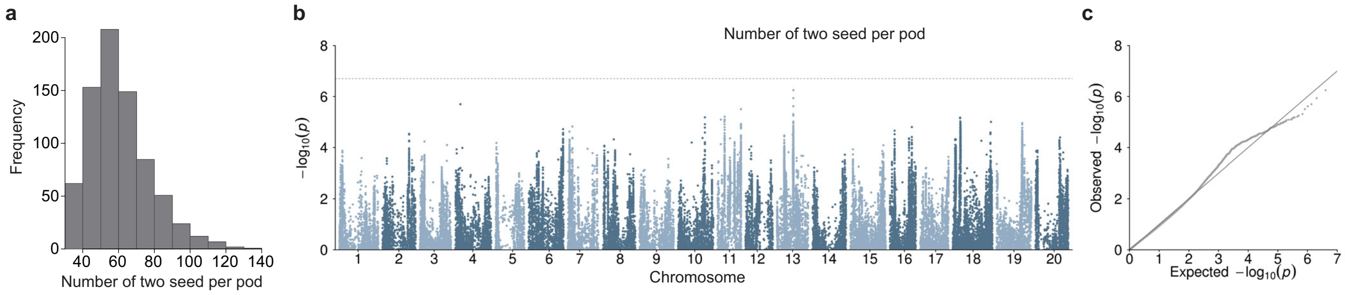

Supplementary Figure 28 GWAS of number of two seed per pod using EMMAX. (**a**) Frequency distribution of number of two seed per pod. (**b**) Manhattan plots for number of two seed per pod. Negative $\log_{10}$ *P*-values from a genome-wide scan are plotted against SNP positions of 20 chromosomes. (**c**) Quantile-quantile plot for number of two seed per pod. The horizontal dash line indicates the significant threshold (2×10^{-7}).

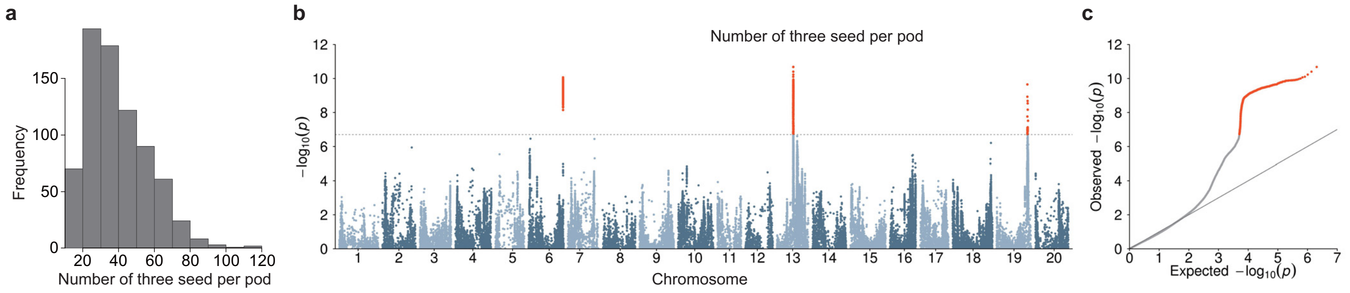

Supplementary Figure 29 GWAS of number of three seed per pod using EMMAX. (**a**) Frequency distribution of number of three seed per pod. (**b**) Manhattan plots for number of three seed per pod. Negative $\log_{10}$ *P*-values from a genome-wide scan are plotted against SNP positions of 20 chromosomes. (**c**) Quantile-quantile plot for number of three seed per pod. The horizontal dash line indicates the significant threshold (2×10^{-7}). Trait-associated SNPs above the significant threshold are colored in red.

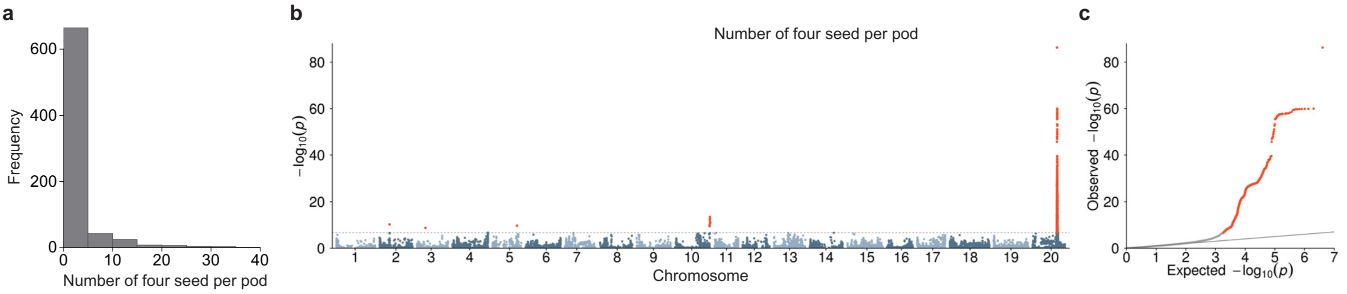

Supplementary Figure 30 GWAS of number of four seed per pod using EMMAX. (**a**) Frequency distribution of number of four seed per pod. (**b**) Manhattan plots for number of four seed per pod. Negative $\log_{10}$ *P*-values from a genome-wide scan are plotted against SNP positions of 20 chromosomes. (**c**) Quantile-quantile plot for number of four seed per pod. The horizontal dash line indicates the significant threshold (5×10^{-9}). Trait-associated SNPs above the significant threshold are colored in red.

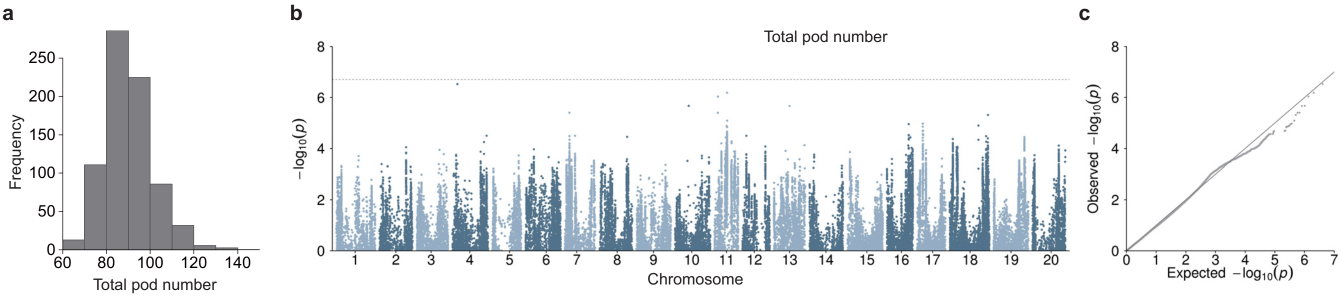

Supplementary Figure 31 GWAS of total pod number using EMMAX. (**a**) Frequency distribution of total pod number. (**b**) Manhattan plots for total pod number. Negative $\log_{10}$ *P*-values from a genome-wide scan are plotted against SNP positions of 20 chromosomes. (**c**) Quantile-quantile plot for total pod number. The horizontal dash line indicates the significant threshold (2×10^{-7}).

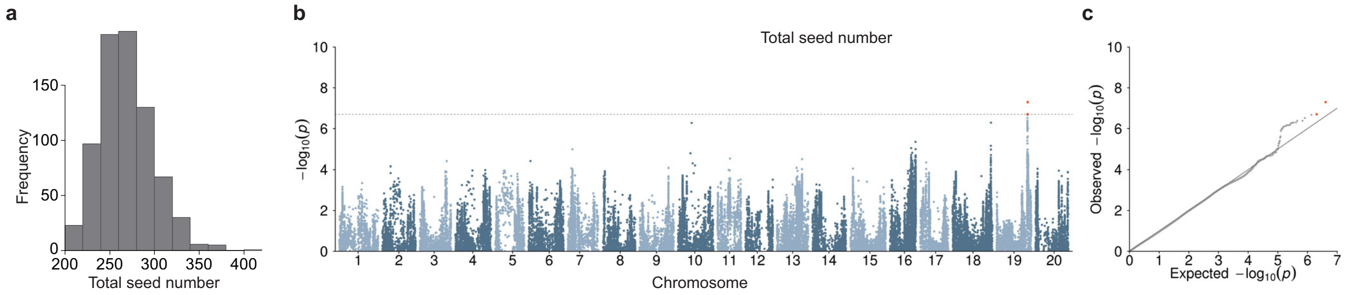

Supplementary Figure 32 GWAS of total seed number using EMMAX. (**a**) Frequency distribution of total seed number. (**b**) Manhattan plots for total seed number. Negative $\log_{10}$ *P*-values from a genome-wide scan are plotted against SNP positions of 20 chromosomes. (**c**) Quantile-quantile plot for total seed number. The horizontal dash line indicates the significant threshold (2×10^{-7}). Trait-associated SNPs above the significant threshold are colored in red.

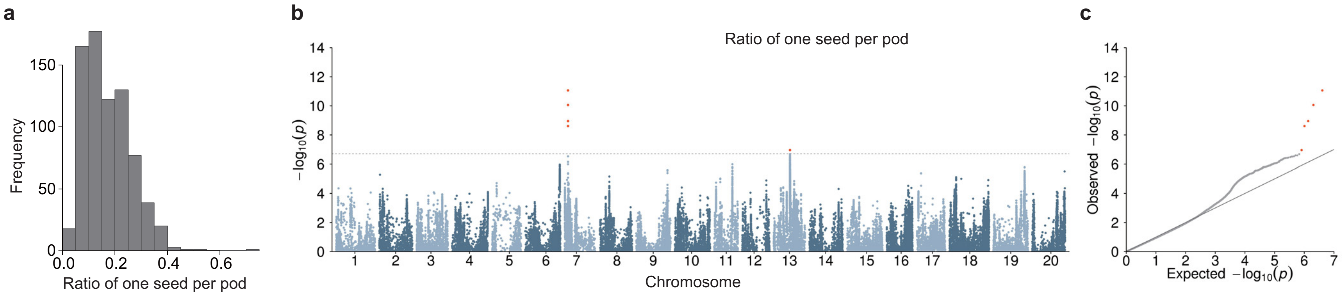

Supplementary Figure 33 GWAS of ratio of one seed per pod using EMMAX. (**a**) Frequency distribution of ratio of one seed per pod. (**b**) Manhattan plots for ratio of one seed per pod. Negative $\log_{10}$ *P*-values from a genome-wide scan are plotted against SNP positions of 20 chromosomes. (**c**) Quantile-quantile plot for ratio of one seed per pod. The horizontal dash line indicates the significant threshold (2×10^{-7}). Trait-associated SNPs above the significant threshold are colored in red.

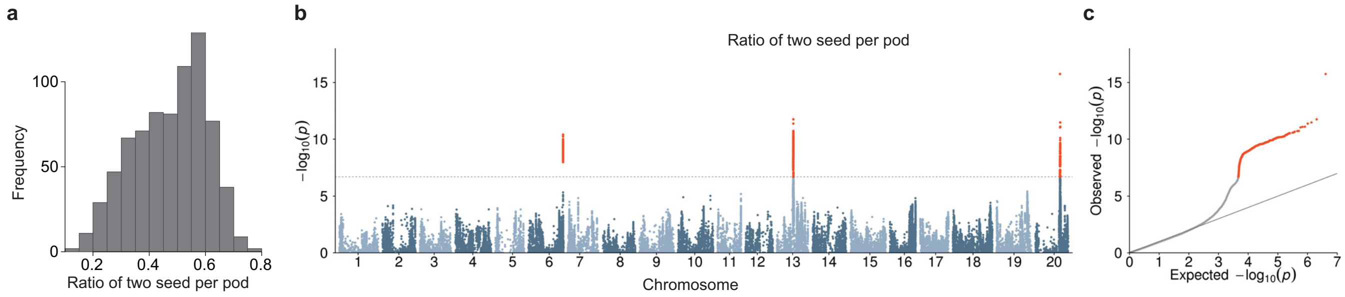

Supplementary Figure 34 GWAS of ratio of two seed per pod using EMMAX. (**a**) Frequency distribution of ratio of two seed per pod. (**b**) Manhattan plots for ratio of two seed per pod. Negative $\log_{10}$ *P*-values from a genome-wide scan are plotted against SNP positions of 20 chromosomes. (**c**) Quantile-quantile plot for ratio of two seed per pod. The horizontal dash line indicates the significant threshold (2×10^{-7}). Trait-associated SNPs above the significant threshold are colored in red.

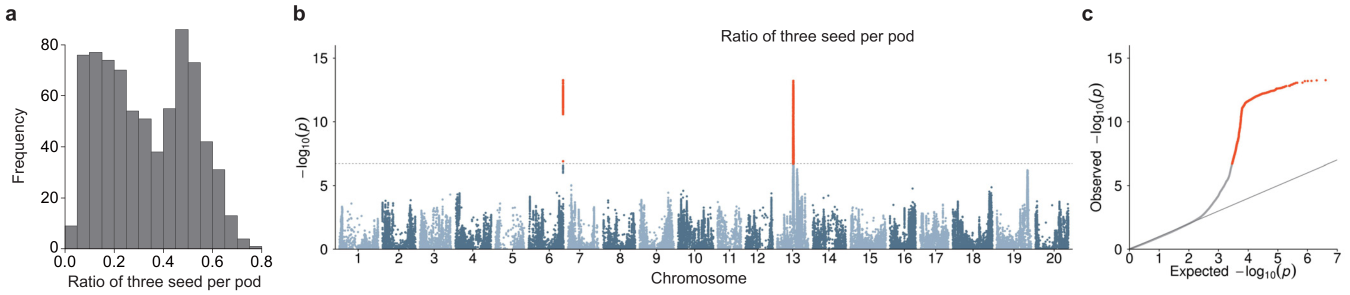

Supplementary Figure 35 GWAS of ratio of three seed per pod using EMMAX. (**a**) Frequency distribution of ratio of three seed per pod. (**b**) Manhattan plots for ratio of three seed per pod. Negative $\log_{10}$ *P*-values from a genome-wide scan are plotted against SNP positions of 20 chromosomes. (**c**) Quantile-quantile plot for ratio of three seed per pod. The horizontal dash line indicates the significant threshold (2×10^{-7}). Trait-associated SNPs above the significant threshold are colored in red.

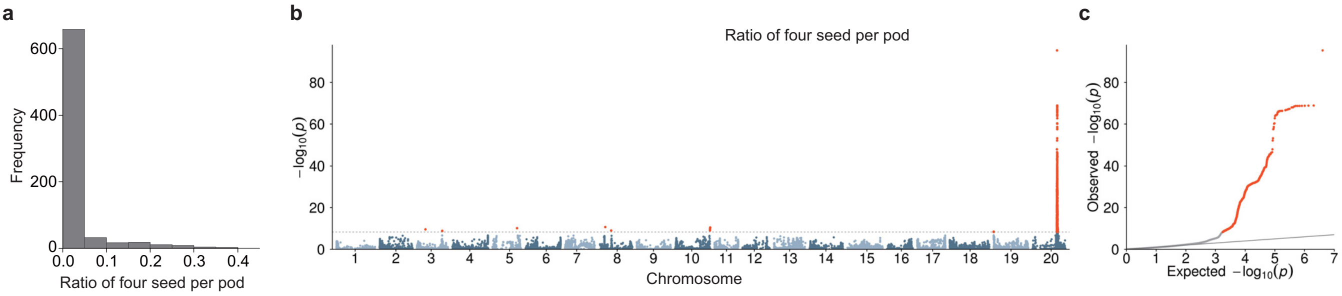

Supplementary Figure 36 GWAS of ratio of four seed per pod using EMMAX. (**a**) Frequency distribution of ratio of four seed per pod. (**b**) Manhattan plots for ratio of four seed per pod. Negative $\log_{10}$ *P*-values from a genome-wide scan are plotted against SNP positions of 20 chromosomes. (**c**) Quantile-quantile plot for ratio of four seed per pod. The horizontal dash line indicates the significant threshold (5×10^{-9}). Trait-associated SNPs above the significant threshold are colored in red.

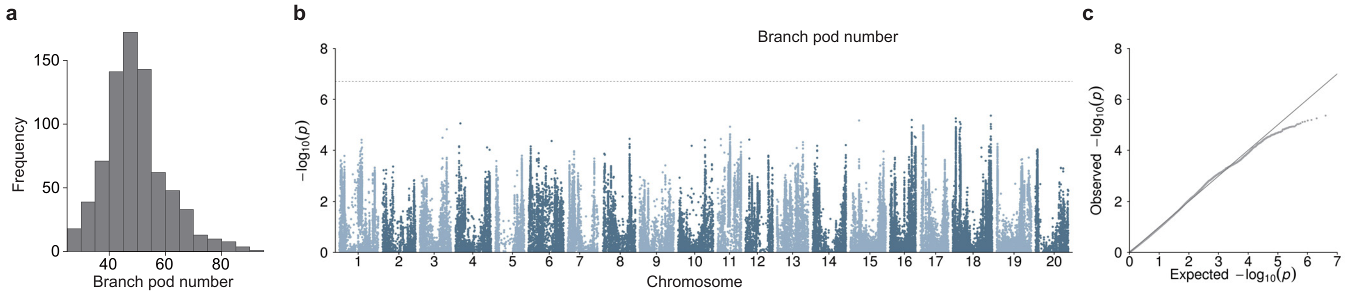

Supplementary Figure 37 GWAS of branch pod number using EMMAX. (**a**) Frequency distribution of branch pod number. (**b**) Manhattan plots for branch pod number. Negative $\log_{10}$ *P*-values from a genome-wide scan are plotted against SNP positions of 20 chromosomes. (**c**) Quantile-quantile plot for branch pod number. The horizontal dash line indicates the significant threshold (2×10^{-7}).

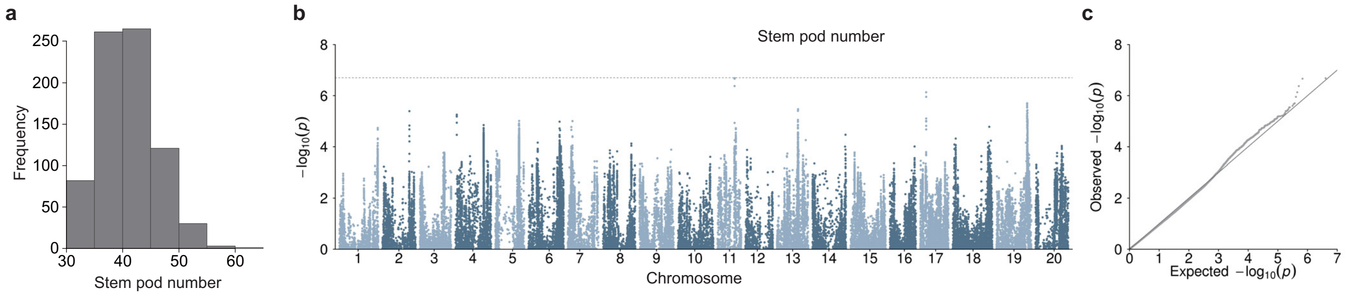

Supplementary Figure 38 GWAS of stem pod number using EMMAX. (**a**) Frequency distribution of stem pod number. (**b**) Manhattan plots for stem pod number. Negative $\log_{10}$ *P*-values from a genome-wide scan are plotted against SNP positions of 20 chromosomes. (**c**) Quantile-quantile plot for stem pod number. The horizontal dash line indicates the significant threshold (2×10^{-7}).

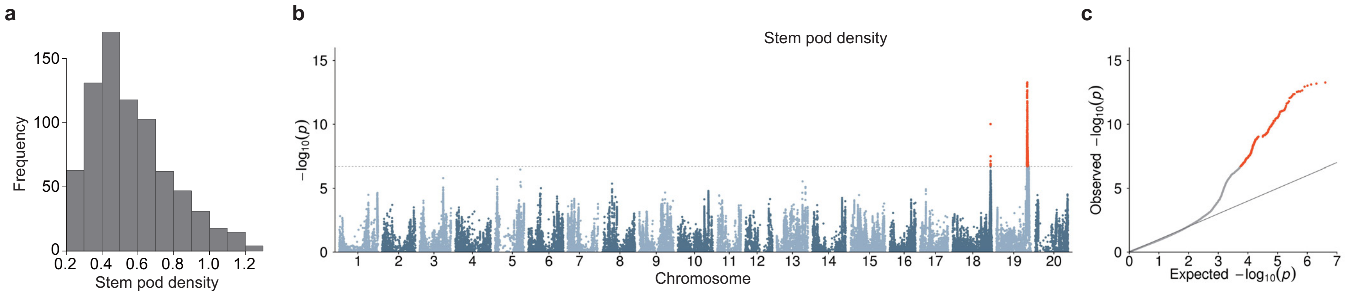

Supplementary Figure 39 GWAS of stem pod density using EMMAX. (**a**) Frequency distribution of stem pod density. (**b**) Manhattan plots for stem pod density. Negative $\log_{10}$ *P*-values from a genome-wide scan are plotted against SNP positions of 20 chromosomes. (**c**) Quantile-quantile plot for stem pod density. The horizontal dash line indicates the significant threshold (2×10^{-7}). Trait-associated SNPs above the significant threshold are colored in red.

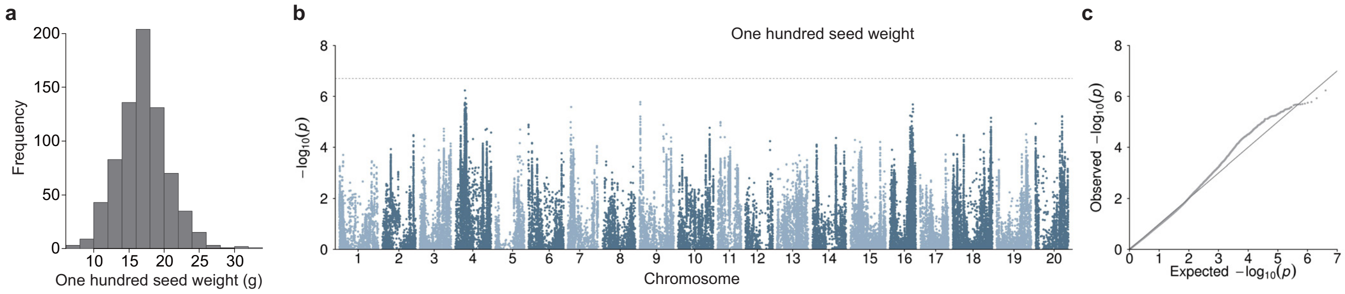

Supplementary Figure 40 GWAS of one hundred seed weight using EMMAX. (**a**) Frequency distribution of one hundred seed weight. (**b**) Manhattan plots for one hundred seed weight. Negative $\log_{10}$ *P*-values from a genome-wide scan are plotted against SNP positions of 20 chromosomes. (**c**) Quantile-quantile plot for one hundred seed weight. The horizontal dash line indicates the significant threshold (2×10^{-7}).

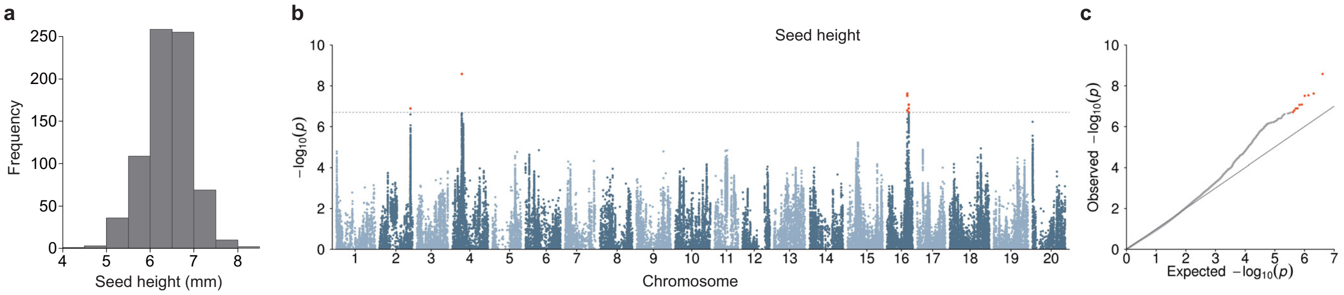

Supplementary Figure 41 GWAS of seed height using EMMAX. (**a**) Frequency distribution of seed height. (**b**) Manhattan plots for seed height. Negative $\log_{10}$ *P*-values from a genome-wide scan are plotted against SNP positions of 20 chromosomes. (**c**) Quantile-quantile plot for seed height. The horizontal dash line indicates the significant threshold (2×10^{-7}). Trait-associated SNPs above the significant threshold are colored in red.

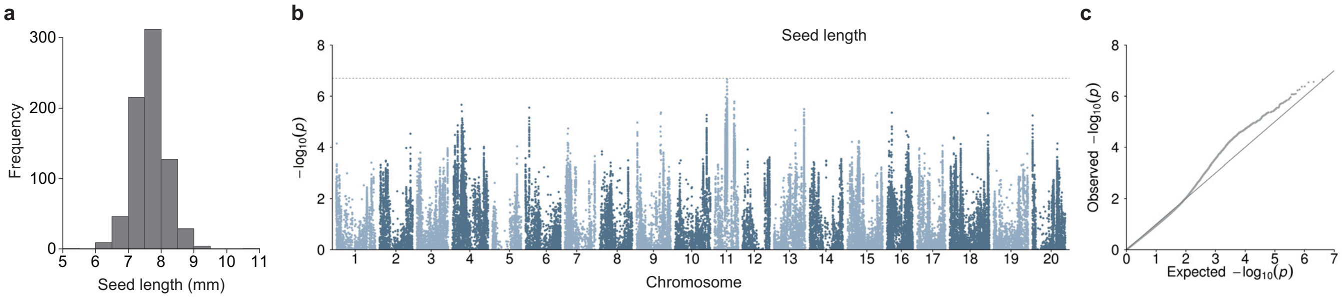

Supplementary Figure 42 GWAS of seed length using EMMAX. (**a**) Frequency distribution of seed length. (**b**) Manhattan plots for seed length. Negative $\log_{10}$ *P*-values from a genome-wide scan are plotted against SNP positions of 20 chromosomes. (**c**) Quantile-quantile plot for seed length. The horizontal dash line indicates the significant threshold (2×10^{-7}).

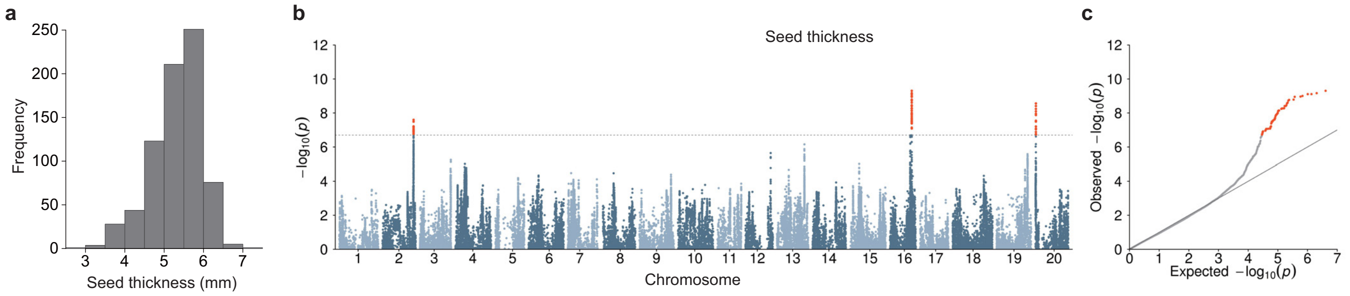

Supplementary Figure 43 GWAS of seed thickness using EMMAX. (**a**) Frequency distribution of seed thickness. (**b**) Manhattan plots for seed thickness. Negative $\log_{10}$ *P*-values from a genome-wide scan are plotted against SNP positions of 20 chromosomes. (**c**) Quantile-quantile plot for seed thickness. The horizontal dash line indicates the significant threshold (2×10^{-7}). Trait-associated SNPs above the significant threshold are colored in red.

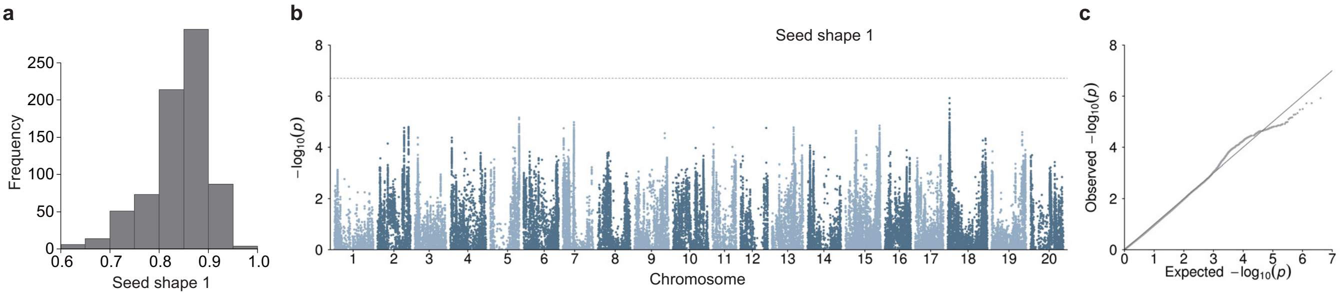

Supplementary Figure 44 GWAS of seed shape 1 using EMMAX. (**a**) Frequency distribution of seed shape 1. (**b**) Manhattan plots for seed shape 1. Negative $\log_{10}$ *P*-values from a genome-wide scan are plotted against SNP positions of 20 chromosomes. (**c**) Quantile-quantile plot for seed shape 1. The horizontal dash line indicates the significant threshold (2×10^{-7}).

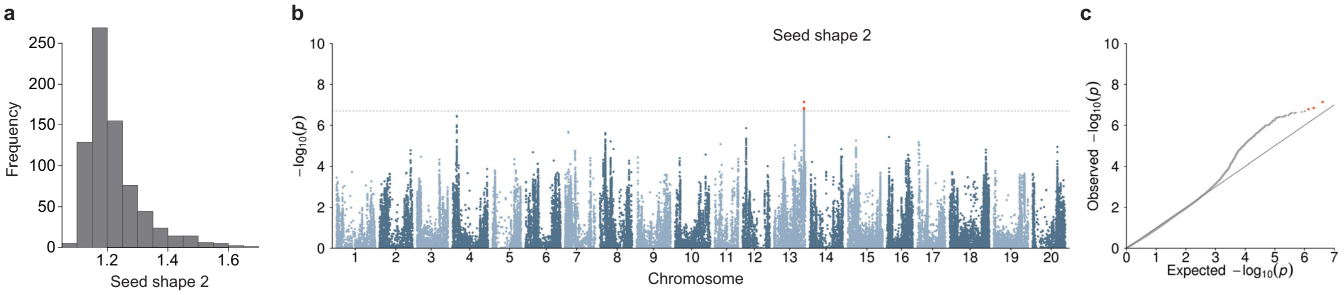

Supplementary Figure 45 GWAS of seed shape 2 using EMMAX. (**a**) Frequency distribution of seed shape 2. (**b**) Manhattan plots for seed shape 2. Negative $\log_{10}$ *P*-values from a genome-wide scan are plotted against SNP positions of 20 chromosomes. (**c**) Quantile-quantile plot for seed shape 2. The horizontal dash line indicates the significant threshold (2×10^{-7}). Trait-associated SNPs above the significant threshold are colored in red.

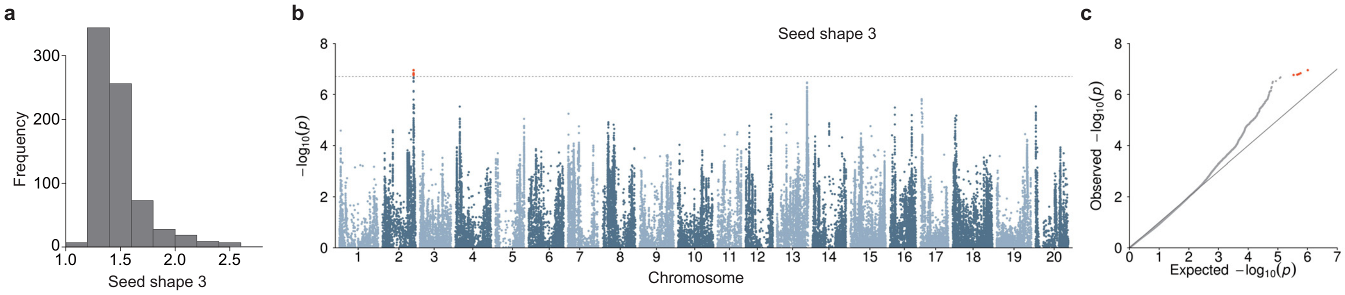

Supplementary Figure 46 GWAS of seed shape 3 using EMMAX. (**a**) Frequency distribution of seed shape 3. (**b**) Manhattan plots for seed shape 3. Negative $\log_{10}$ *P*-values from a genome-wide scan are plotted against SNP positions of 20 chromosomes. (**c**) Quantile-quantile plot for seed shape 3. The horizontal dash line indicates the significant threshold (2×10^{-7}). Trait-associated SNPs above the significant threshold are colored in red.

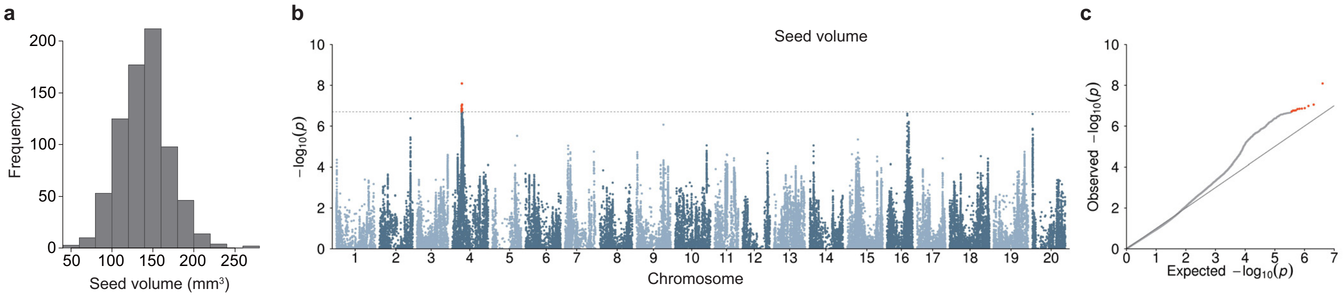

Supplementary Figure 47 GWAS of seed volume using EMMAX. (**a**) Frequency distribution of seed volume. (**b**) Manhattan plots for seed volume. Negative $\log_{10}$ *P*-values from a genome-wide scan are plotted against SNP positions of 20 chromosomes. (**c**) Quantile-quantile plot for seed volume. The horizontal dash line indicates the significant threshold (2×10^{-7}). Trait-associated SNPs above the significant threshold are colored in red.

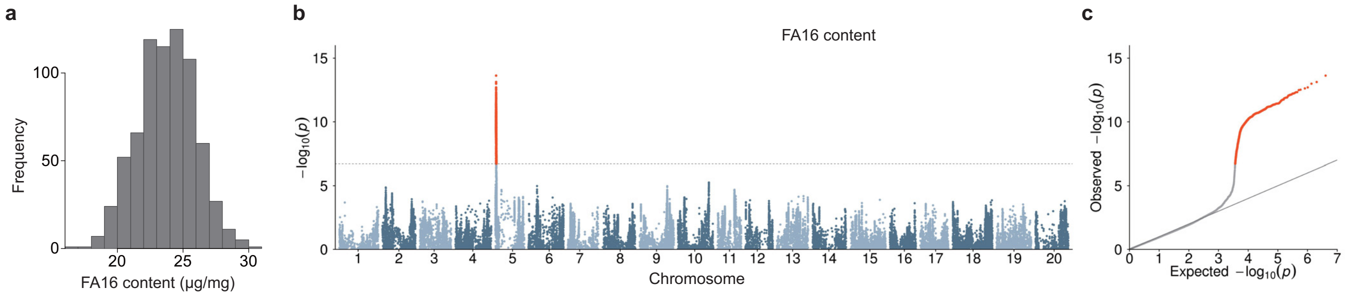

Supplementary Figure 48 GWAS of FA16 content using EMMAX. (**a**) Frequency distribution of FA16 content. (**b**) Manhattan plots for FA16 content. Negative $\log_{10}$ *P*-values from a genome-wide scan are plotted against SNP positions of 20 chromosomes. (**c**) Quantile-quantile plot for FA16 content. The horizontal dash line indicates the significant threshold (2×10^{-7}). Trait-associated SNPs above the significant threshold are colored in red. FA, fatty acid.

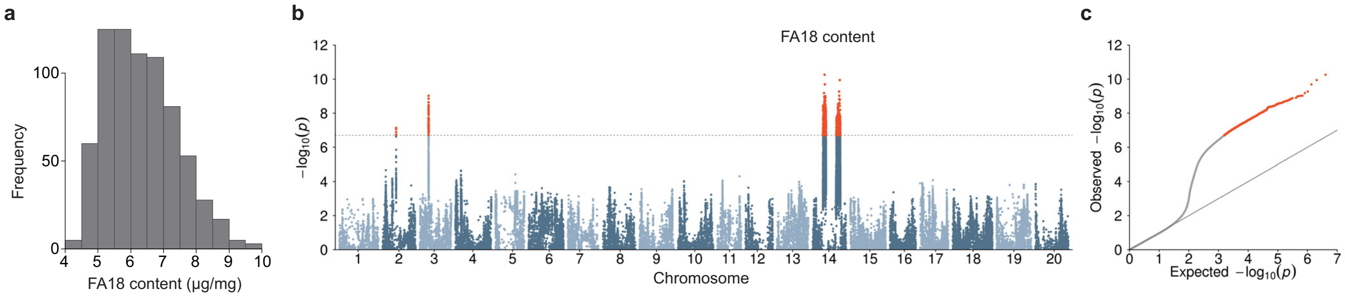

Supplementary Figure 49 GWAS of FA18 content using EMMAX. (**a**) Frequency distribution of FA18 content. (**b**) Manhattan plots for FA18 content. Negative $\log_{10}$ *P*-values from a genome-wide scan are plotted against SNP positions of 20 chromosomes. (**c**) Quantile-quantile plot for FA18 content. The horizontal dash line indicates the significant threshold (2×10^{-7}). Trait-associated SNPs above the significant threshold are colored in red. FA, fatty acid.

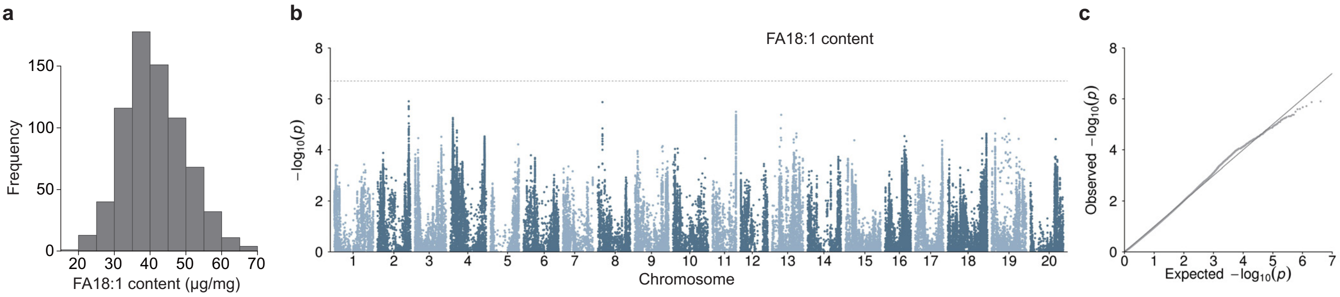

Supplementary Figure 50 GWAS of FA18:1 content using EMMAX. (**a**) Frequency distribution of FA18:1 content. (**b**) Manhattan plots for FA18:1 content. Negative $\log_{10}$ *P*-values from a genome-wide scan are plotted against SNP positions of 20 chromosomes. (**c**) Quantile-quantile plot for FA18:1 content. The horizontal dash line indicates the significant threshold (2×10^{-7}). FA, fatty acid.

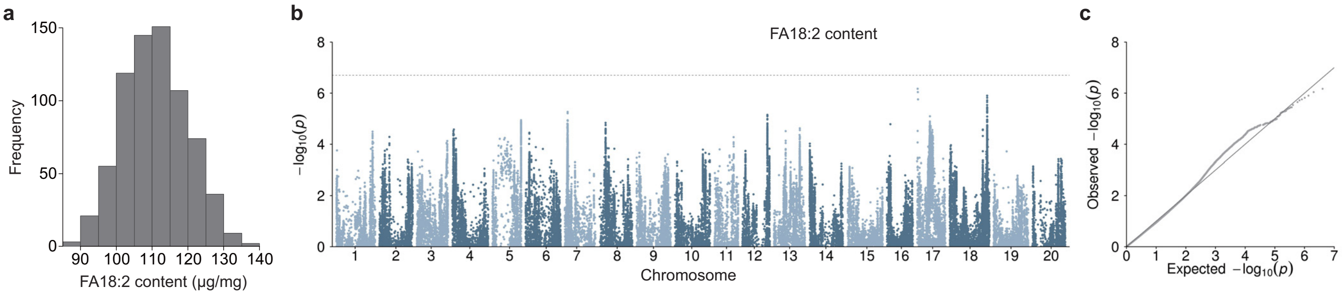

Supplementary Figure 51 GWAS of FA18:2 content using EMMAX. (**a**) Frequency distribution of FA18:2 content. (**b**) Manhattan plots for FA18:2 content. Negative $\log_{10}$ *P*-values from a genome-wide scan are plotted against SNP positions of 20 chromosomes. (**c**) Quantile-quantile plot for FA18:2 content. The horizontal dash line indicates the significant threshold (2×10^{-7}). FA, fatty acid.

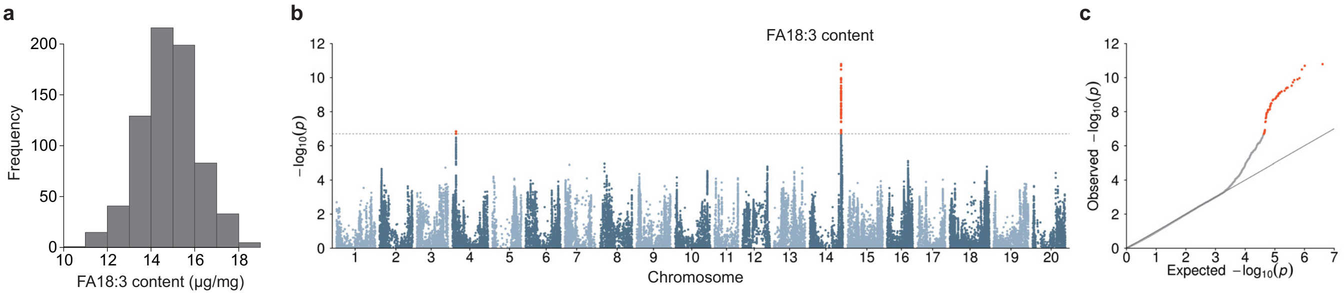

Supplementary Figure 52 GWAS of FA18:3 content using EMMAX. (**a**) Frequency distribution of FA18:3 content. (**b**) Manhattan plots for FA18:3 content. Negative $\log_{10}$ *P*-values from a genome-wide scan are plotted against SNP positions of 20 chromosomes. (**c**) Quantile-quantile plot for FA18:3 content. The horizontal dash line indicates the significant threshold (2×10^{-7}). Trait-associated SNPs above the significant threshold are colored in red. FA, fatty acid.

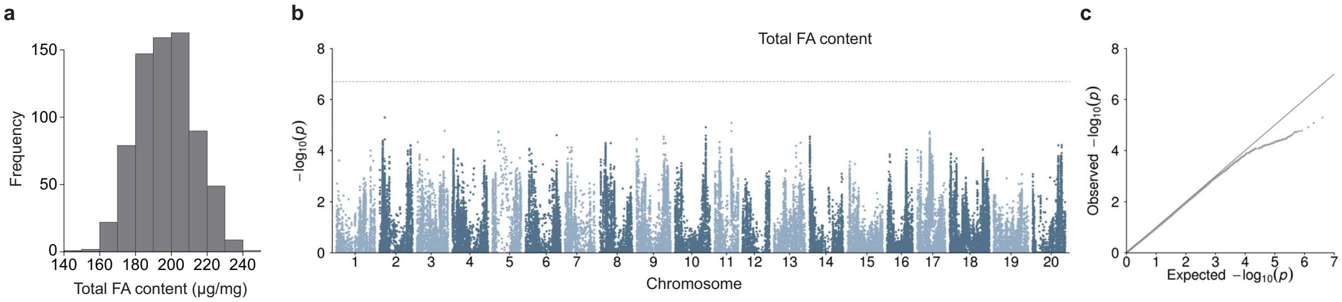

Supplementary Figure 53 GWAS of total FA content using EMMAX. (**a**) Frequency distribution of total FA content. (**b**) Manhattan plots for total FA content. Negative $\log_{10}$ *P*-values from a genome-wide scan are plotted against SNP positions of 20 chromosomes. (**c**) Quantile-quantile plot for total FA content. The horizontal dash line indicates the significant threshold ($2{\times}10^{-7}$). FA, fatty acid.

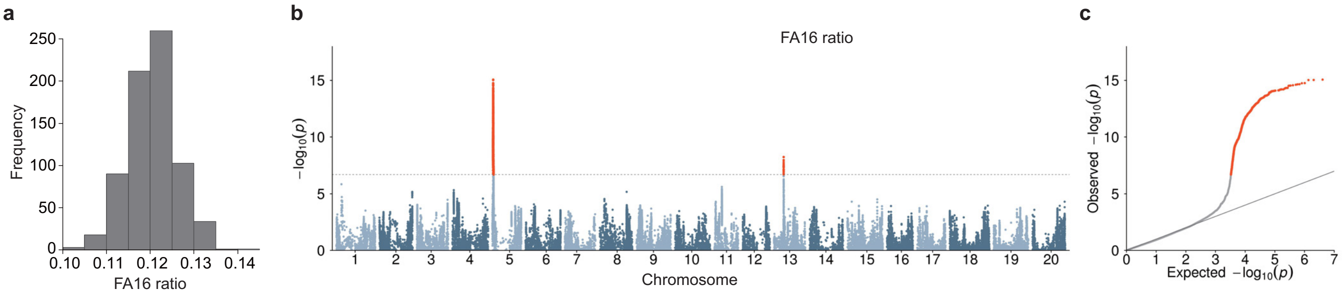

Supplementary Figure 54 GWAS of FA16 ratio using EMMAX. (**a**) Frequency distribution of FA16 ratio. (**b**) Manhattan plots for FA16 ratio. Negative $\log_{10}$ *P*-values from a genome-wide scan are plotted against SNP positions of 20 chromosomes. (**c**) Quantile-quantile plot for FA16 ratio. The horizontal dash line indicates the significant threshold (2×10^{-7}). Trait-associated SNPs above the significant threshold are colored in red. FA, fatty acid.

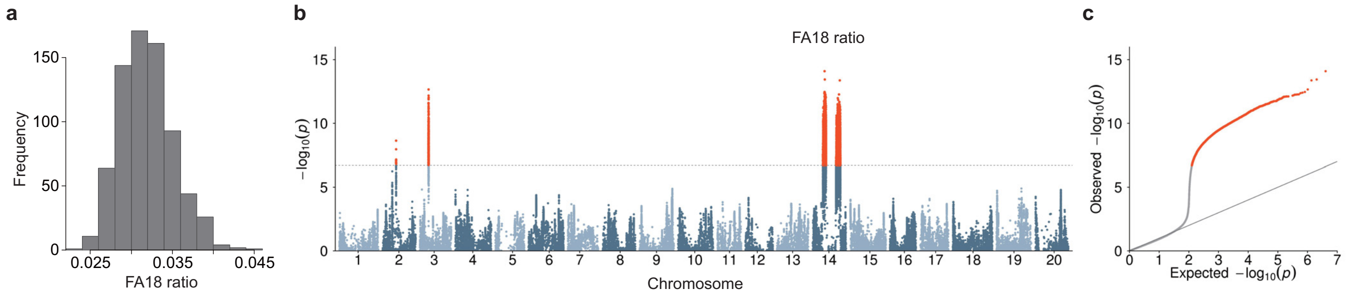

Supplementary Figure 55 GWAS of FA18 ratio using EMMAX. (**a**) Frequency distribution of FA18 ratio. (**b**) Manhattan plots for FA18 ratio. Negative $\log_{10}$ *P*-values from a genome-wide scan are plotted against SNP positions of 20 chromosomes. (**c**) Quantile-quantile plot for FA18 ratio. The horizontal dash line indicates the significant threshold (2×10^{-7}). Trait-associated SNPs above the significant threshold are colored in red. FA, fatty acid.

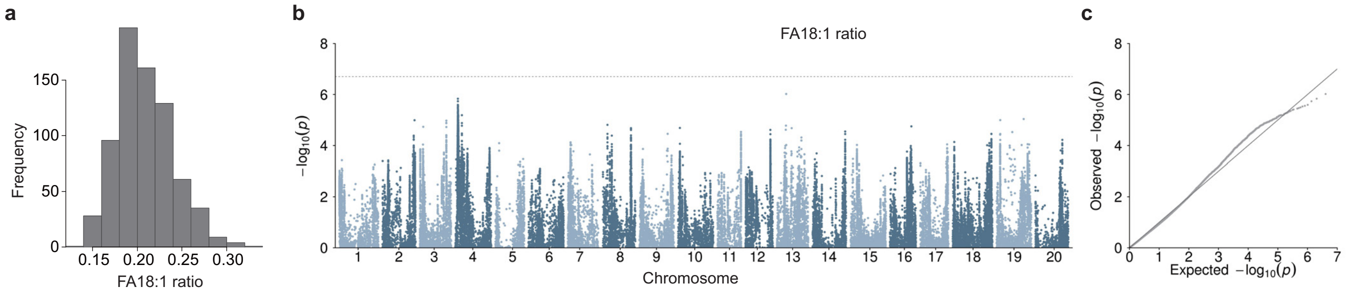

Supplementary Figure 56 GWAS of FA18:1 ratio using EMMAX. (**a**) Frequency distribution of FA18:1 ratio. (**b**) Manhattan plots for FA18:1 ratio. Negative $\log_{10}$ *P*-values from a genome-wide scan are plotted against SNP positions of 20 chromosomes. (**c**) Quantile-quantile plot for FA18:1 ratio. The horizontal dash line indicates the significant threshold (2×10^{-7}). FA, fatty acid.

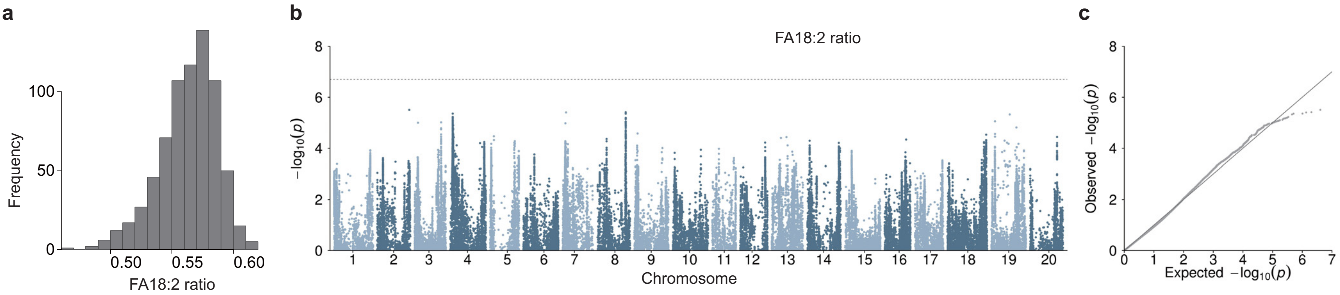

Supplementary Figure 57 GWAS of FA18:2 ratio using EMMAX. (**a**) Frequency distribution of FA18:2 ratio. (**b**) Manhattan plots for FA18:2 ratio. Negative $\log_{10}$ *P*-values from a genome-wide scan are plotted against SNP positions of 20 chromosomes. (**c**) Quantile-quantile plot for FA18:2 ratio. The horizontal dash line indicates the significant threshold (2×10^{-7}). FA, fatty acid.

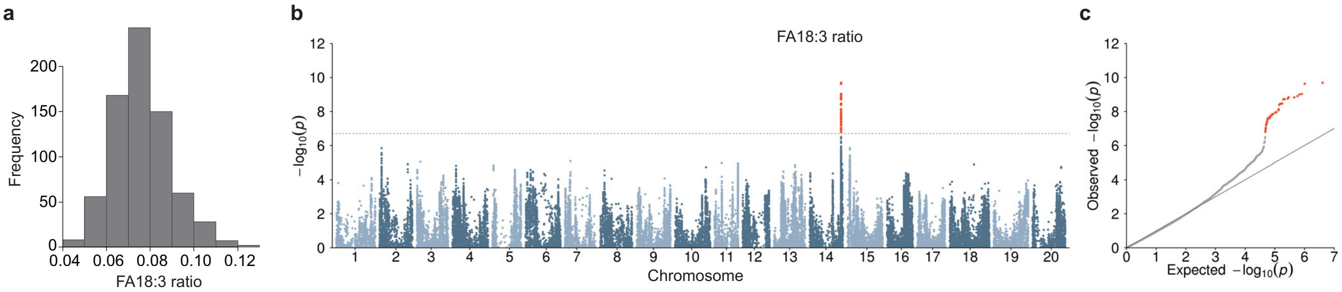

Supplementary Figure 58 GWAS of FA18:3 ratio using EMMAX. (**a**) Frequency distribution of FA18:3 ratio. (**b**) Manhattan plots for FA18:3 ratio. Negative $\log_{10}$ *P*-values from a genome-wide scan are plotted against SNP positions of 20 chromosomes. (**c**) Quantile-quantile plot for FA18:3 ratio. The horizontal dash line indicates the significant threshold (2×10^{-7}). Trait-associated SNPs above the significant threshold are colored in red. FA, fatty acid.

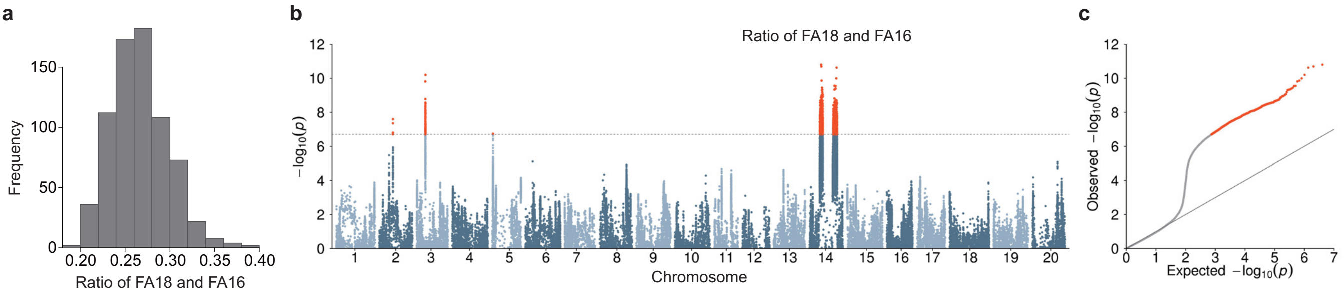

Supplementary Figure 59 GWAS of ratio of FA18 and FA16 using EMMAX. (**a**) Frequency distribution of ratio of FA18 and FA16. (**b**) Manhattan plots for ratio of FA18 and FA16. Negative $\log_{10}$ *P*-values from a genome-wide scan are plotted against SNP positions of 20 chromosomes. (**c**) Quantile-quantile plot for ratio of FA18 and FA16. The horizontal dash line indicates the significant threshold (2×10^{-7}). Trait-associated SNPs above the significant threshold are colored in red. FA, fatty acid.

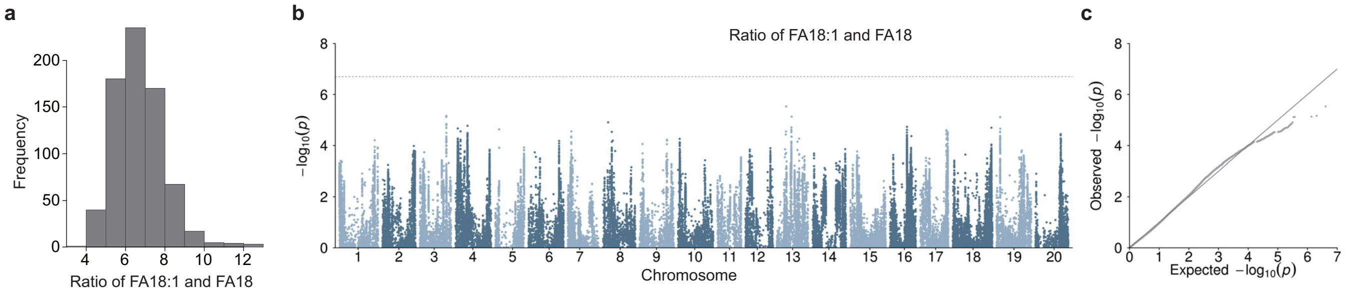

Supplementary Figure 60 GWAS of ratio of FA18:1 and FA18 using EMMAX. (**a**) Frequency distribution of ratio of FA18:1 and FA18. (**b**) Manhattan plots for ratio of FA18:1 and FA18. Negative $\log_{10}$ *P*-values from a genome-wide scan are plotted against SNP positions of 20 chromosomes. (**c**) Quantile-quantile plot for ratio of FA18:1 and FA18. The horizontal dash line indicates the significant threshold (2×10^{-7}). FA, fatty acid.

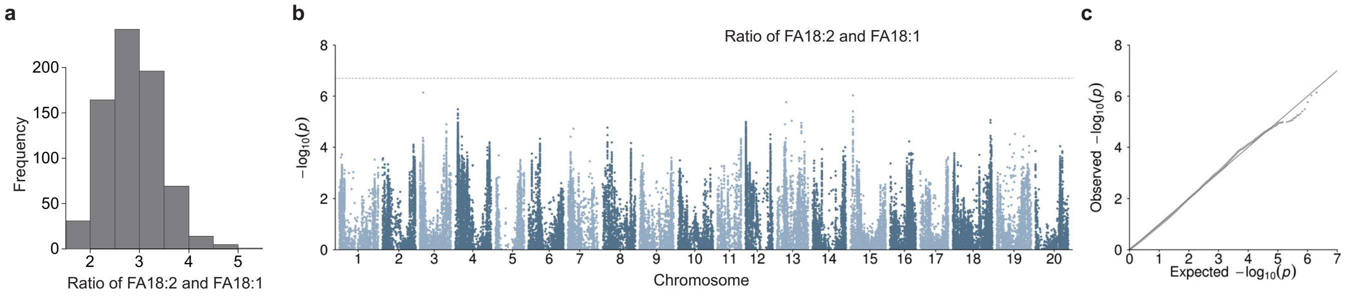

Supplementary Figure 61 GWAS of ratio of FA18:2 and FA18:1 using EMMAX. (**a**) Frequency distribution of ratio of FA18:2 and FA18:1. (**b**) Manhattan plots for ratio of FA18:2 and FA18:1. Negative $\log_{10}$ *P*-values from a genome-wide scan are plotted against SNP positions of 20 chromosomes. (**c**) Quantile-quantile plot for ratio of FA18:2 and FA18:1. The horizontal dash line indicates the significant threshold (2×10^{-7}). FA, fatty acid.

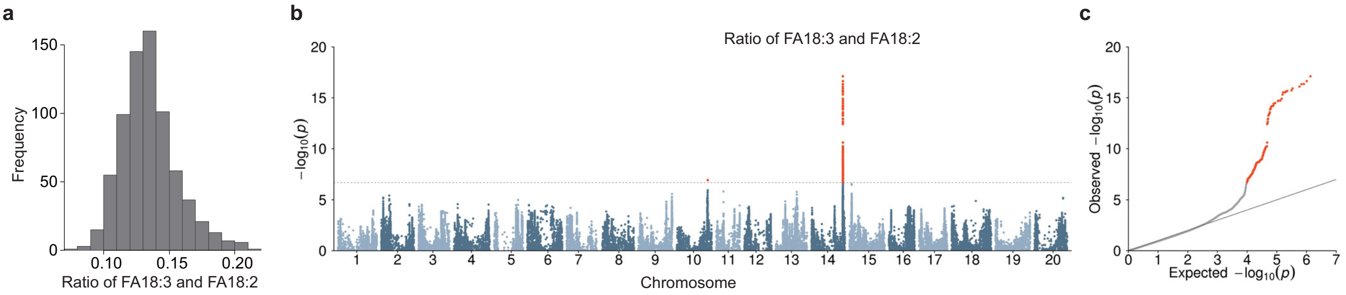

Supplementary Figure 62 GWAS of ratio of FA18:3 and FA18:2 using EMMAX. (**a**) Frequency distribution of ratio of FA18:3 and FA18:2. (**b**) Manhattan plots for ratio of FA18:3 and FA18:2. Negative $\log_{10}$ *P*-values from a genome-wide scan are plotted against SNP positions of 20 chromosomes. (**c**) Quantile-quantile plot for ratio of FA18:3 and FA18:2. The horizontal dash line indicates the significant threshold (2×10^{-7}). Trait-associated SNPs above the significant threshold are colored in red. FA, fatty acid.

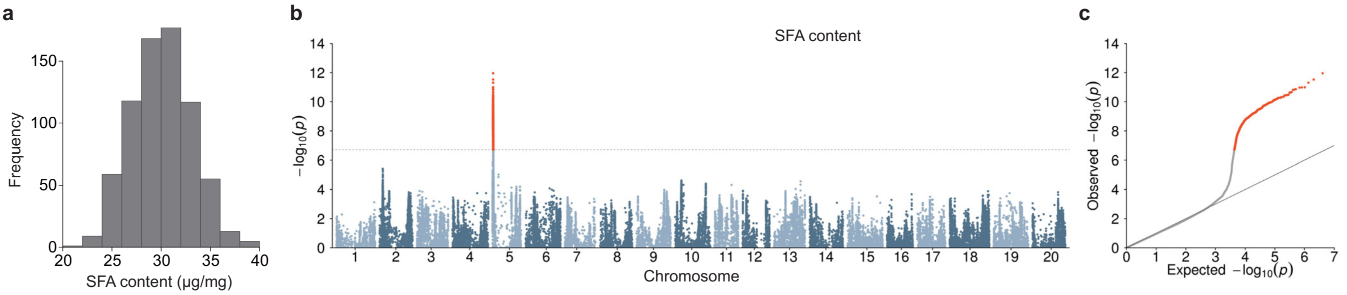

Supplementary Figure 63 GWAS of SFA content using EMMAX. (**a**) Frequency distribution of SFA content. (**b**) Manhattan plots for SFA content. Negative $\log_{10}$ *P*-values from a genome-wide scan are plotted against SNP positions of 20 chromosomes. (**c**) Quantile-quantile plot for SFA content. The horizontal dash line indicates the significant threshold (2×10^{-7}). Trait-associated SNPs above the significant threshold are colored in red. SFA, saturated fatty acid.

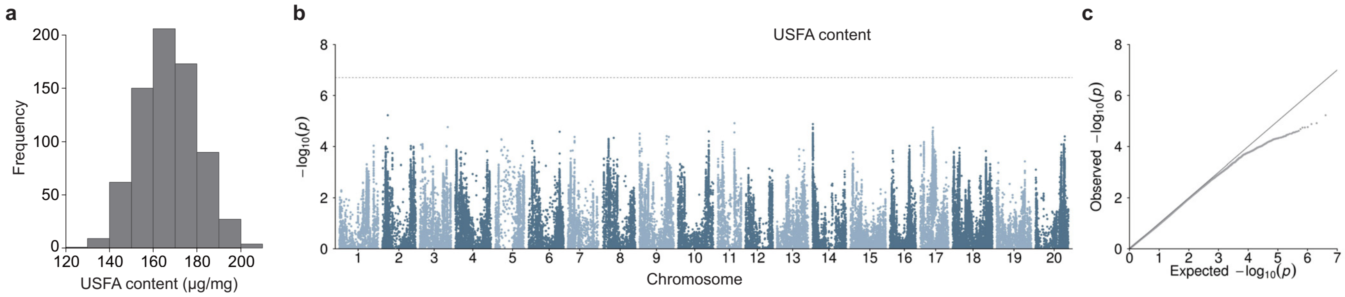

Supplementary Figure 64 GWAS of USFA content using EMMAX. (**a**) Frequency distribution of USFA content. (**b**) Manhattan plots for USFA content. Negative $\log_{10}$ *P*-values from a genome-wide scan are plotted against SNP positions of 20 chromosomes. (**c**) Quantile-quantile plot for USFA content. The horizontal dash line indicates the significant threshold (2×10^{-7}). USFA, unsaturated fatty acid.

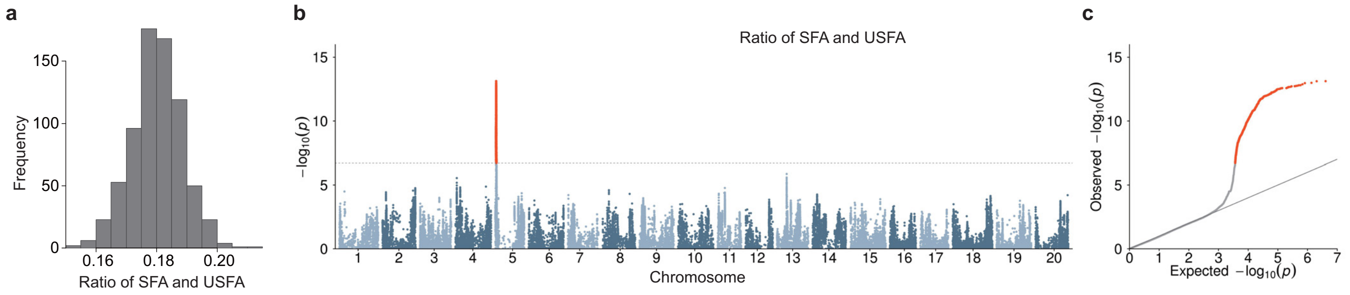

Supplementary Figure 65 GWAS of ratio of SFA and USFA using EMMAX. (**a**) Frequency distribution of ratio of SFA and USFA. (**b**) Manhattan plots for ratio of SFA and USFA. Negative $\log_{10}$ *P*-values from a genome-wide scan are plotted against SNP positions of 20 chromosomes. (**c**) Quantile-quantile plot for ratio of SFA and USFA. The horizontal dash line indicates the significant threshold (2×10^{-7}). Trait-associated SNPs above the significant threshold are colored in red. SFA, saturated fatty acid; USFA, unsaturated fatty acid.

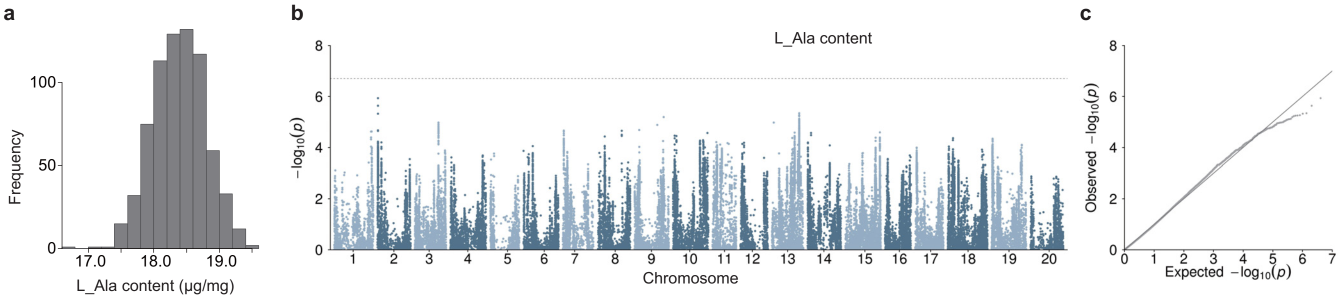

Supplementary Figure 66 GWAS of L_Ala content using EMMAX. (**a**) Frequency distribution of L_Ala content. (**b**) Manhattan plots for L_Ala content. Negative $\log_{10}$ *P*-values from a genome-wide scan are plotted against SNP positions of 20 chromosomes. (**c**) Quantile-quantile plot for L_Ala content. The horizontal dash line indicates the significant threshold ($2{\times}10^{-7}$).

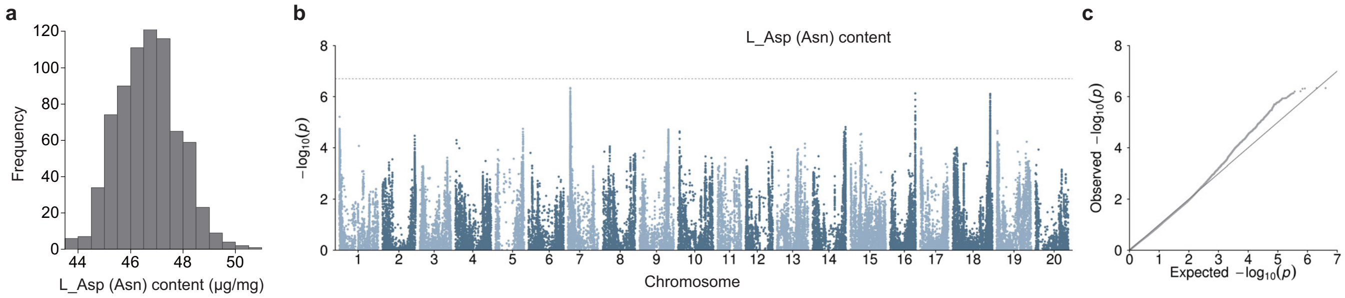

Supplementary Figure 67 GWAS of L_Asp (Asn) content using EMMAX. (**a**) Frequency distribution of L_Asp (Asn) content. (**b**) Manhattan plots for L_Asp (Asn) content. Negative $\log_{10}$ *P*-values from a genome-wide scan are plotted against SNP positions of 20 chromosomes. (**c**) Quantile-quantile plot for L_Asp (Asn) content. The horizontal dash line indicates the significant threshold (2×10^{-7}).

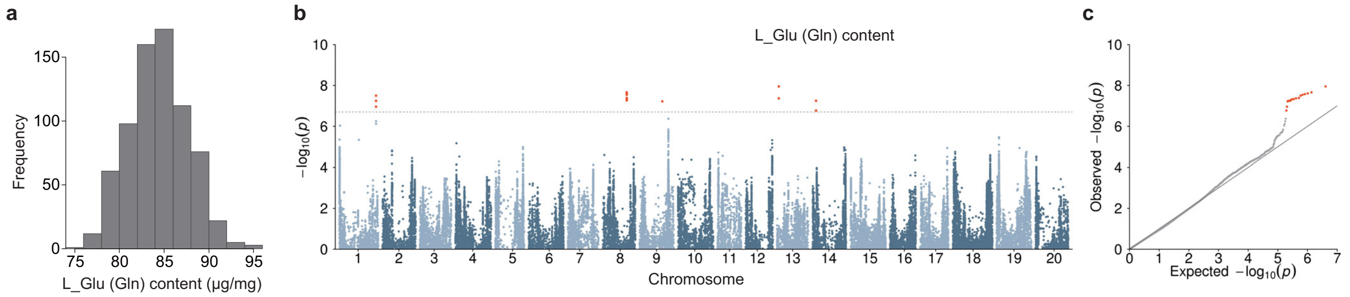

Supplementary Figure 68 GWAS of L_Glu (Gln) content using EMMAX. (**a**) Frequency distribution of L_Glu (Gln) content. (**b**) Manhattan plots for L_Glu (Gln) content. Negative $\log_{10}$ *P*-values from a genome-wide scan are plotted against SNP positions of 20 chromosomes. (**c**) Quantile-quantile plot for L_Glu (Gln) content. The horizontal dash line indicates the significant threshold ($2{\times}10^{-7}$). Trait-associated SNPs above the significant threshold are colored in red.

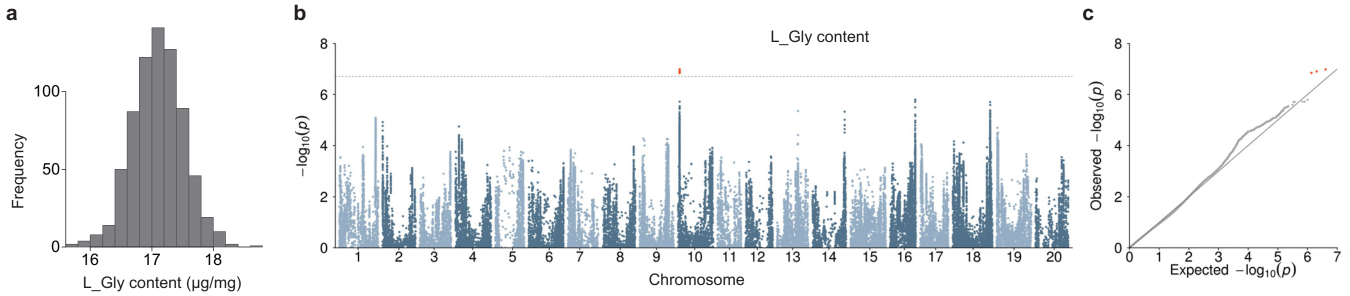

Supplementary Figure 69 GWAS of L_Gly content using EMMAX. (**a**) Frequency distribution of L_Gly content. (**b**) Manhattan plots for L_Gly content. Negative $\log_{10}$ *P*-values from a genome-wide scan are plotted against SNP positions of 20 chromosomes. (**c**) Quantile-quantile plot for L_Gly content. The horizontal dash line indicates the significant threshold (2×10^{-7}). Trait-associated SNPs above the significant threshold are colored in red.

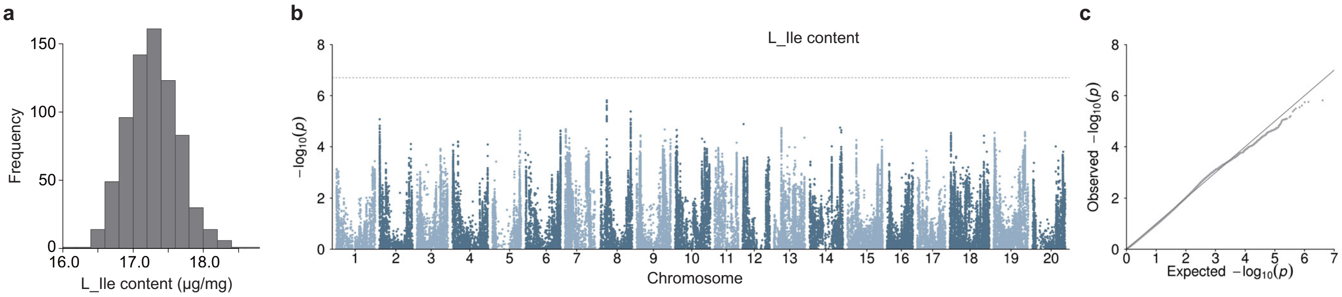

Supplementary Figure 70 GWAS of L_Ile content using EMMAX. (**a**) Frequency distribution of L_Ile content. (**b**) Manhattan plots for L_Ile content. Negative log_{10} *P*-values from a genome-wide scan are plotted against SNP positions of 20 chromosomes. (**c**) Quantile-quantile plot for L_Ile content. The horizontal dash line indicates the significant threshold ($2×10^{-7}$).

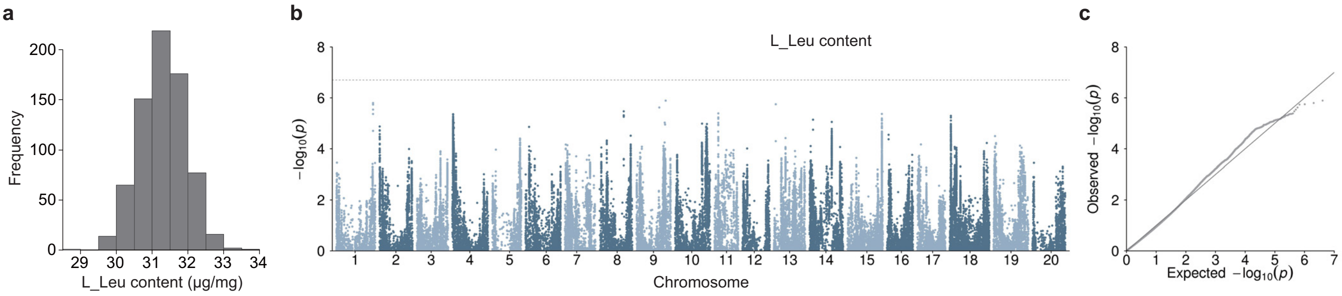

Supplementary Figure 71 GWAS of L_Leu content using EMMAX. (**a**) Frequency distribution of L_Leu content. (**b**) Manhattan plots for L_Leu content. Negative $\log_{10}$ *P*-values from a genome-wide scan are plotted against SNP positions of 20 chromosomes. (**c**) Quantile-quantile plot for L_Leu content. The horizontal dash line indicates the significant threshold (2×10^{-7}).

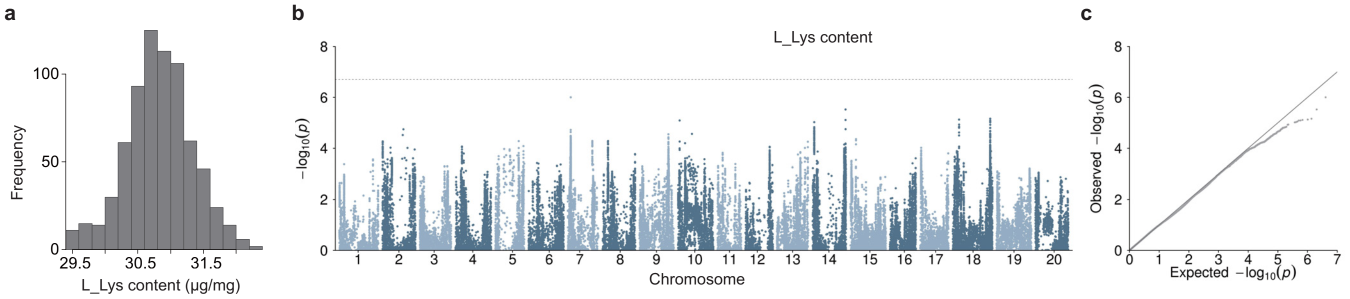

Supplementary Figure 72 GWAS of L_Lys content using EMMAX. (**a**) Frequency distribution of L_Lys content. (**b**) Manhattan plots for L_Lys content. Negative $\log_{10}$ *P*-values from a genome-wide scan are plotted against SNP positions of 20 chromosomes. (**c**) Quantile-quantile plot for L_Lys content. The horizontal dash line indicates the significant threshold (2×10^{-7}).

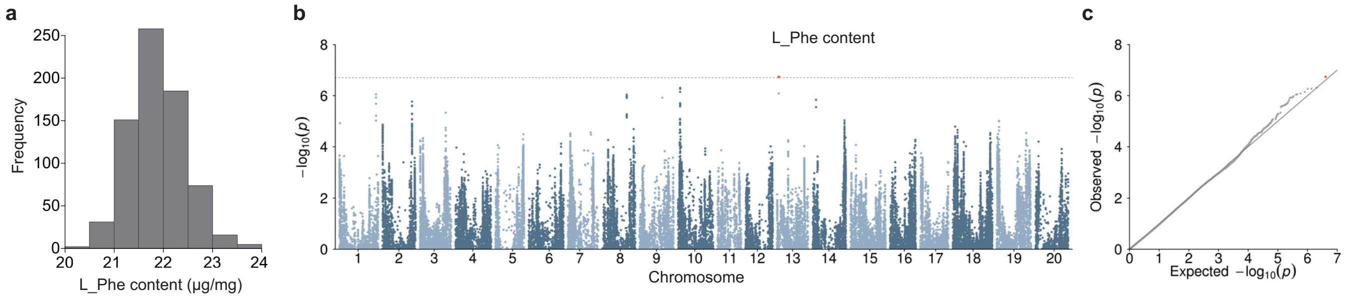

Supplementary Figure 73 GWAS of L_Phe content using EMMAX. (**a**) Frequency distribution of L_Phe content. (**b**) Manhattan plots for L_Phe content. Negative $\log_{10}$ *P*-values from a genome-wide scan are plotted against SNP positions of 20 chromosomes. (**c**) Quantile-quantile plot for L_Phe content. The horizontal dash line indicates the significant threshold (2×10^{-7}). Trait-associated SNPs above the significant threshold are colored in red.

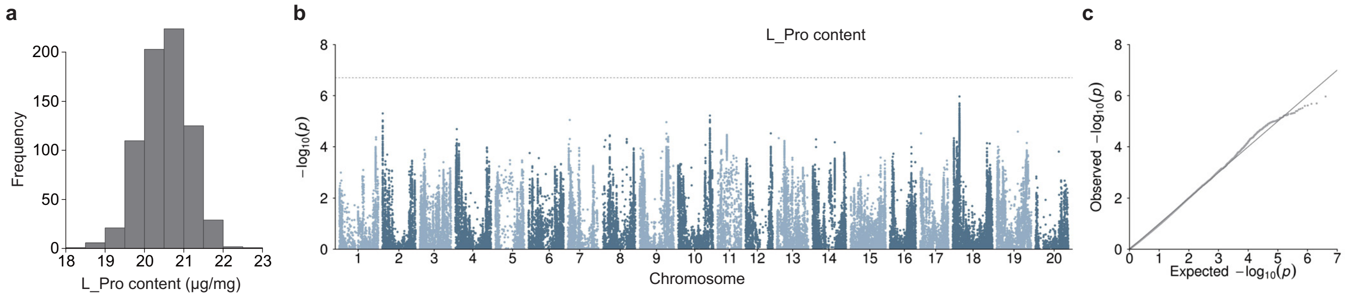

Supplementary Figure 74 GWAS of L_Pro content using EMMAX. (**a**) Frequency distribution of L_Pro content. (**b**) Manhattan plots for L_Pro content. Negative $\log_{10}$ *P*-values from a genome-wide scan are plotted against SNP positions of 20 chromosomes. (**c**) Quantile-quantile plot for L_Pro content. The horizontal dash line indicates the significant threshold (2×10^{-7}).

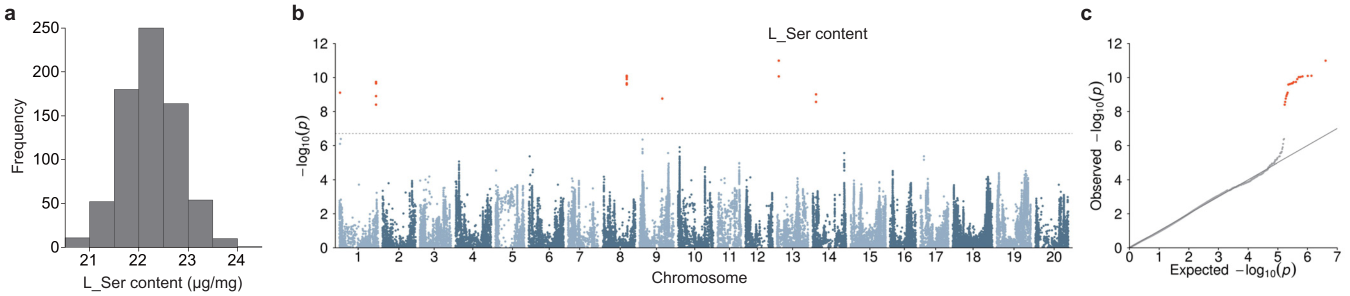

Supplementary Figure 75 GWAS of L_Ser content using EMMAX. (**a**) Frequency distribution of L_Ser content. (**b**) Manhattan plots for L_Ser content. Negative $\log_{10}$ *P*-values from a genome-wide scan are plotted against SNP positions of 20 chromosomes. (**c**) Quantile-quantile plot for L_Ser content. The horizontal dash line indicates the significant threshold (2×10^{-7}). Trait-associated SNPs above the significant threshold are colored in red.

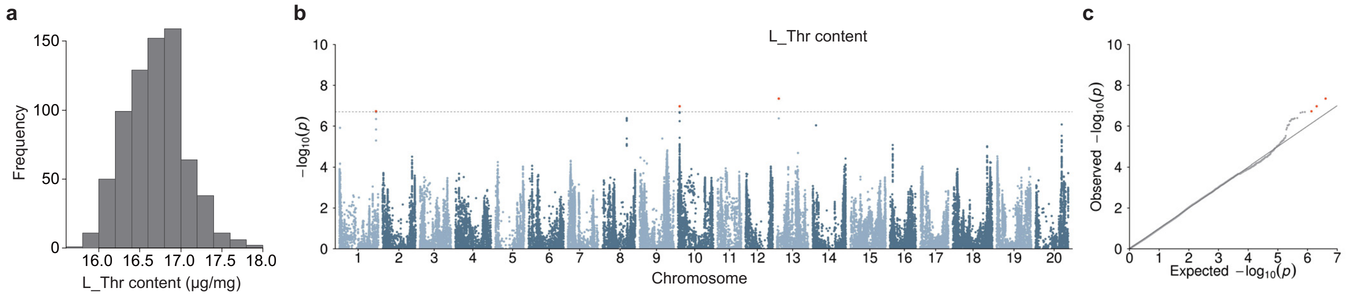

Supplementary Figure 76 GWAS of L_Thr content using EMMAX. (**a**) Frequency distribution of L_Thr content. (**b**) Manhattan plots for L_Thr content. Negative $\log_{10}$ *P*-values from a genome-wide scan are plotted against SNP positions of 20 chromosomes. (**c**) Quantile-quantile plot for L_Thr content. The horizontal dash line indicates the significant threshold ($2{\times}10^{-7}$). Trait-associated SNPs above the significant threshold are colored in red.

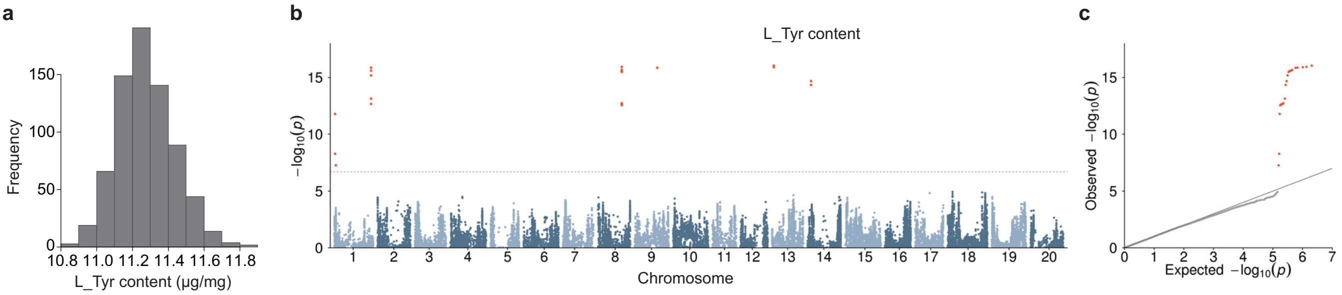

Supplementary Figure 77 GWAS of L_Tyr content using EMMAX. (**a**) Frequency distribution of L_Tyr content. (**b**) Manhattan plots for L_Tyr content. Negative $\log_{10}$ *P*-values from a genome-wide scan are plotted against SNP positions of 20 chromosomes. (**c**) Quantile-quantile plot for L_Tyr content. The horizontal dash line indicates the significant threshold ($2×10^{-7}$). Trait-associated SNPs above the significant threshold are colored in red.

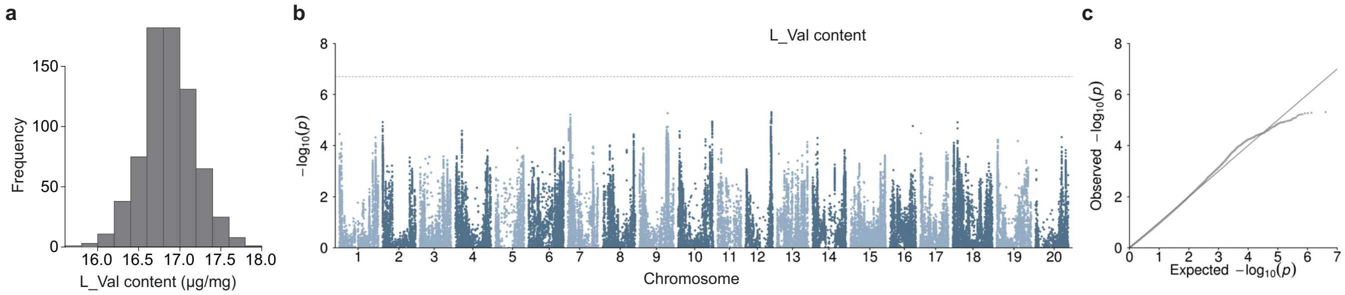

Supplementary Figure 78 GWAS of L_Val content using EMMAX. (**a**) Frequency distribution of L_Val content. (**b**) Manhattan plots for L_Val content. Negative $\log_{10}$ *P*-values from a genome-wide scan are plotted against SNP positions of 20 chromosomes. (**c**) Quantile-quantile plot for L_Val content. The horizontal dash line indicates the significant threshold (2×10^{-7}).

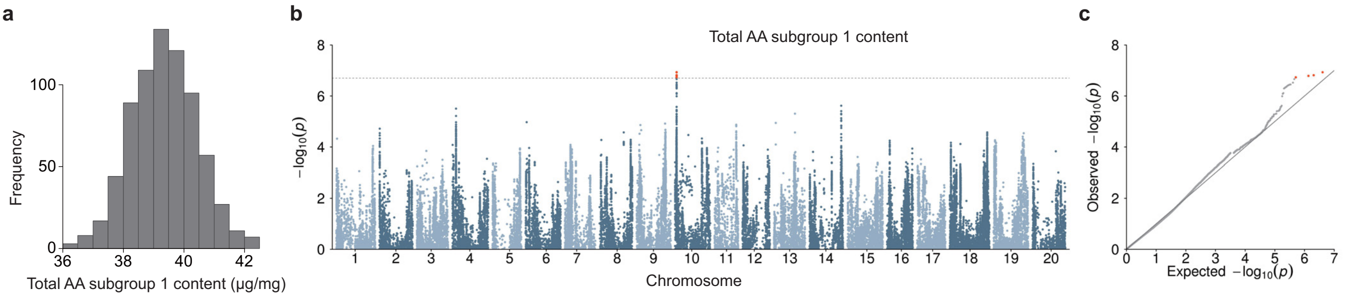

Supplementary Figure 79 GWAS of total AA subgroup 1 using EMMAX. (**a**) Frequency distribution of total AA subgroup 1 content. (**b**) Manhattan plots for total AA subgroup 1. Negative $\log_{10}$ *P*-values from a genome-wide scan are plotted against SNP positions of 20 chromosomes. (**c**) Quantile-quantile plot for total AA subgroup 1. The horizontal dash line indicates the significant threshold (2×10^{-7}). Trait-associated SNPs above the significant threshold are colored in red. AA, amino acid.

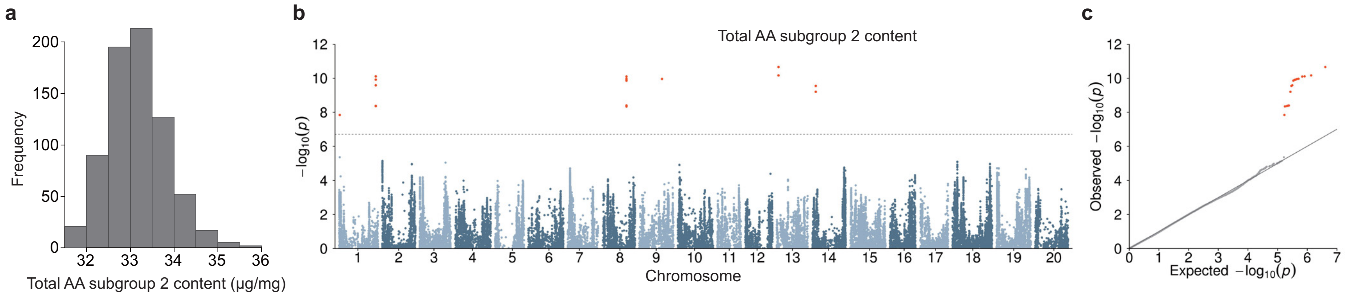

Supplementary Figure 80 GWAS of total AA subgroup 2 using EMMAX. (**a**) Frequency distribution of total AA subgroup 2 content. (**b**) Manhattan plots for total AA subgroup 2. Negative $\log_{10}$ *P*-values from a genome-wide scan are plotted against SNP positions of 20 chromosomes. (**c**) Quantile-quantile plot for total AA subgroup 2. The horizontal dash line indicates the significant threshold (2×10^{-7}). Trait-associated SNPs above the significant threshold are colored in red. AA, amino acid.

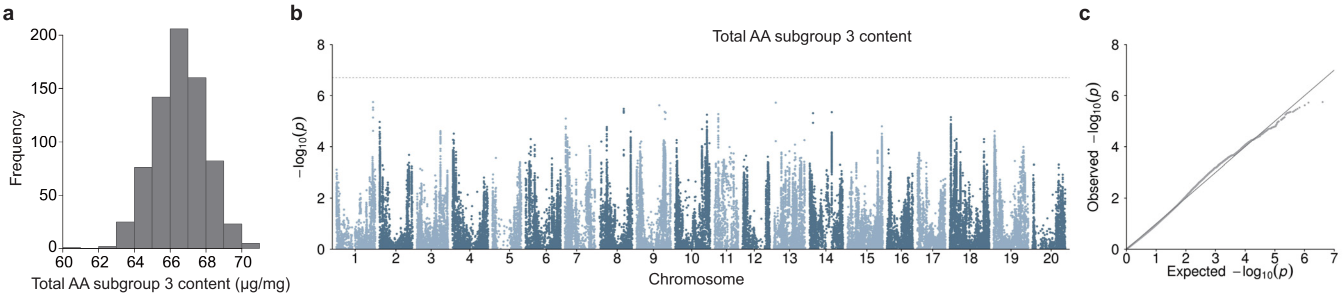

Supplementary Figure 81 GWAS of total AA subgroup 3 using EMMAX. (**a**) Frequency distribution of total AA subgroup 3 content. (**b**) Manhattan plots for total AA subgroup 3. Negative $\log_{10}$ *P*-values from a genome-wide scan are plotted against SNP positions of 20 chromosomes. (**c**) Quantile-quantile plot for total AA subgroup 3. The horizontal dash line indicates the significant threshold (2×10^{-7}). AA, amino acid.

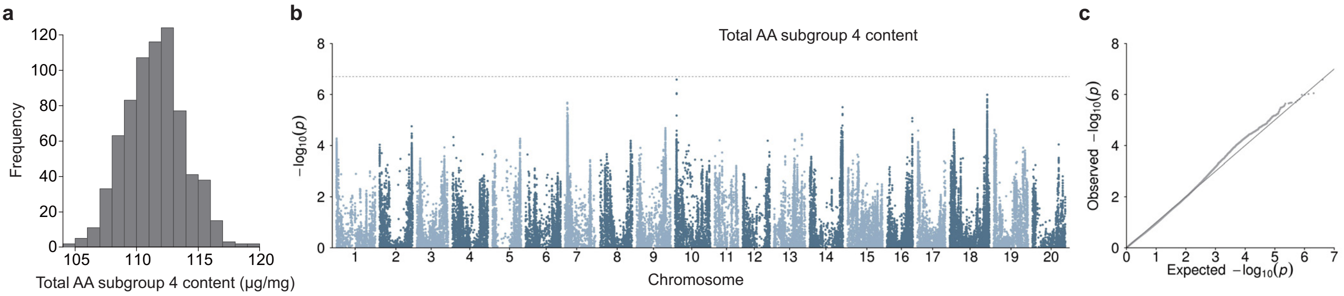

Supplementary Figure 82 GWAS of total AA subgroup 4 using EMMAX. (**a**) Frequency distribution of total AA subgroup 4 content. (**b**) Manhattan plots for total AA subgroup 4. Negative log_{10} *P*-values from a genome-wide scan are plotted against SNP positions of 20 chromosomes. (**c**) Quantile-quantile plot for total AA subgroup 4. The horizontal dash line indicates the significant threshold (2×10^{-7}). AA, amino acid.

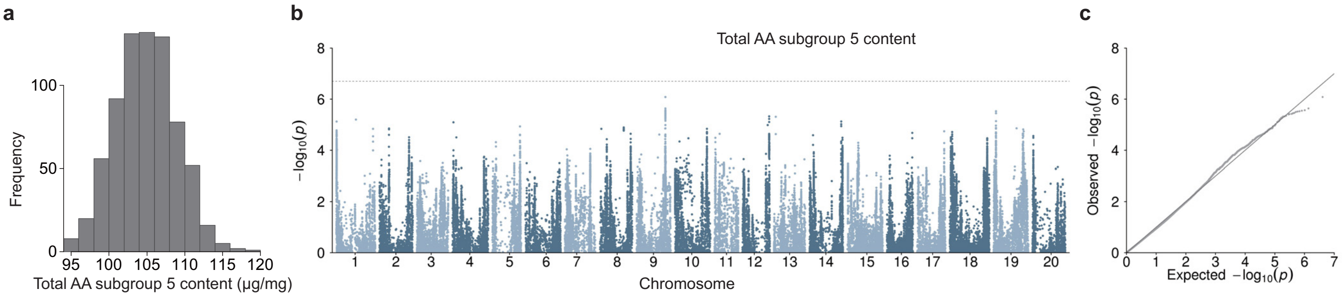

Supplementary Figure 83 GWAS of total AA subgroup 5 content using EMMAX. (**a**) Frequency distribution of total AA subgroup 5 content. (**b**) Manhattan plots for total AA subgroup 5 content. Negative $\log_{10}$ *P*-values from a genome-wide scan are plotted against SNP positions of 20 chromosomes. (**c**) Quantile-quantile plot for total AA subgroup 5 content. The horizontal dash line indicates the significant threshold (2×10^{-7}). AA, amino acid.

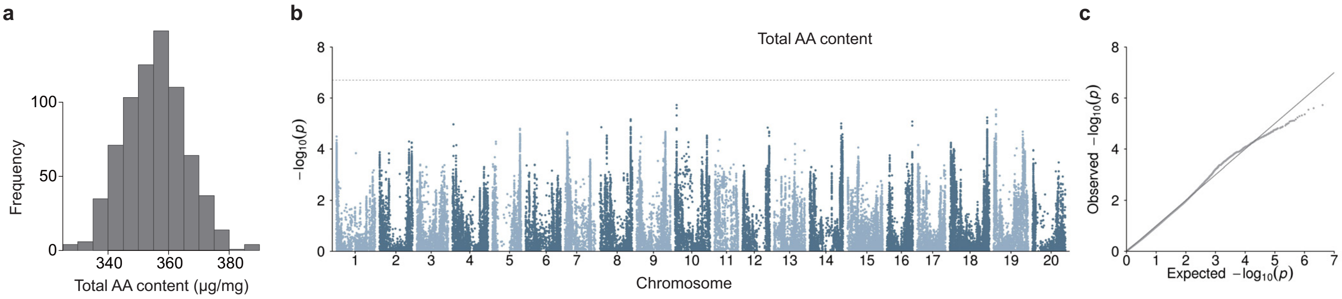

Supplementary Figure 84 GWAS of total AA content using EMMAX. (**a**) Frequency distribution of total AA content. (**b**) Manhattan plots for total AA content. Negative $\log_{10}$ *P*-values from a genome-wide scan are plotted against SNP positions of 20 chromosomes. (**c**) Quantile-quantile plot for total AA content. The horizontal dash line indicates the significant threshold (2×10^{-7}). AA, amino acid.

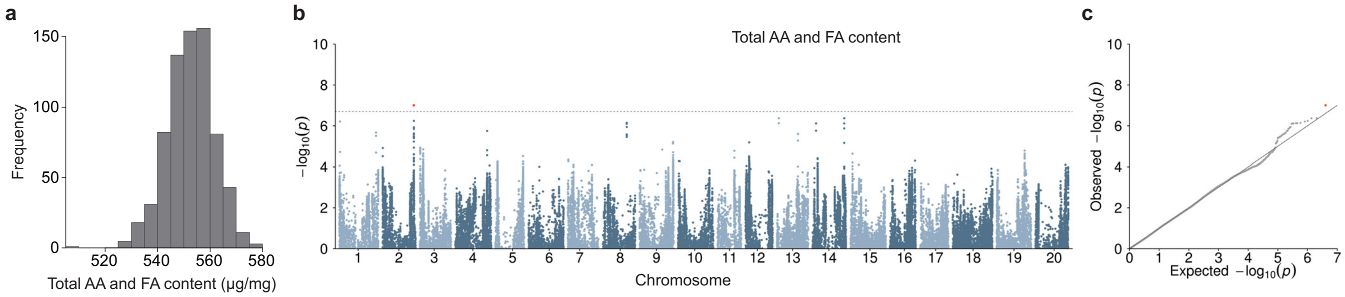

Supplementary Figure 85 GWAS of total AA and FA content using EMMAX. (**a**) Frequency distribution of total AA and FA content. (**b**) Manhattan plots for total AA and FA content. Negative $\log_{10}$ *P*-values from a genome-wide scan are plotted against SNP positions of 20 chromosomes. (**c**) Quantile-quantile plot for total AA and FA content. The horizontal dash line indicates the significant threshold (2×10^{-7}). Trait-associated SNPs above the significant threshold are colored in red. AA, amino acid; FA, fatty acid.

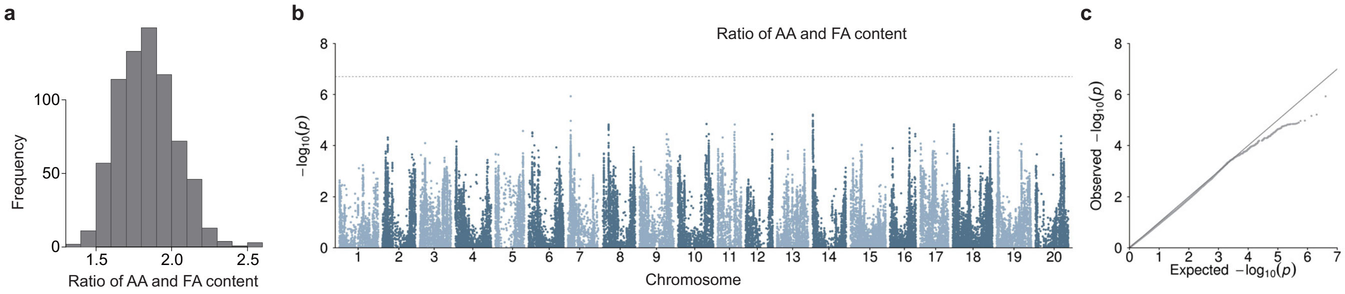

Supplementary Figure 86 GWAS of ratio of AA and FA content using EMMAX. (**a**) Frequency distribution of ratio of AA and FA content. (**b**) Manhattan plots for ratio of AA and FA content. Negative $\log_{10}$ *P*-values from a genome-wide scan are plotted against SNP positions of 20 chromosomes. (**c**) Quantile-quantile plot for ratio of AA and FA content. The horizontal dash line indicates the significant threshold (2×10^{-7}). AA, amino acid; FA, fatty acid.

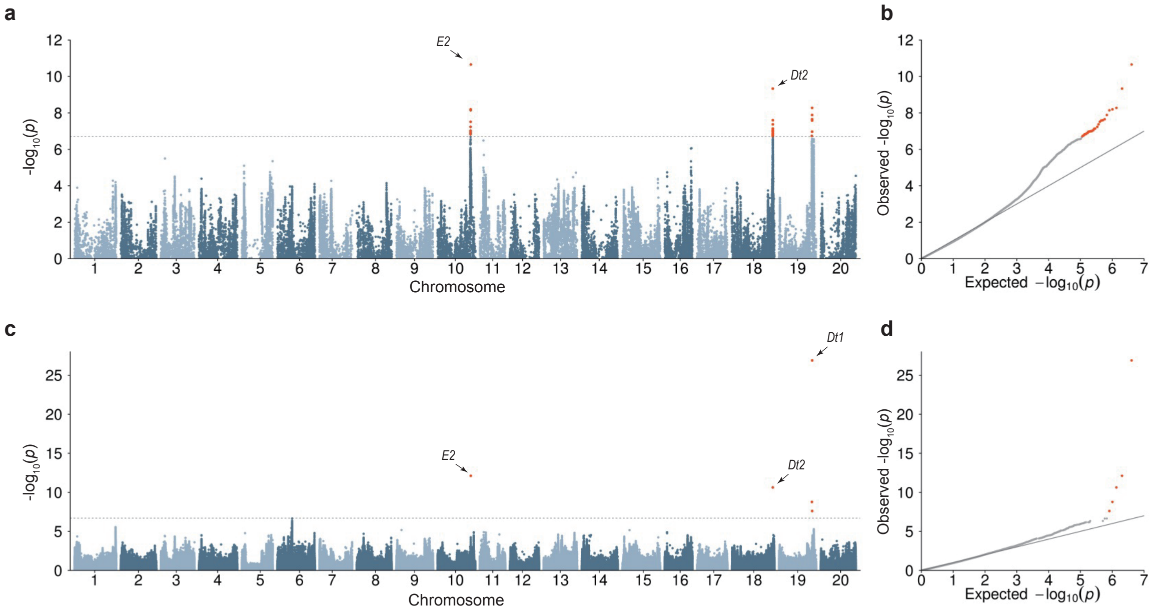

Supplementary Figure 87 Confirmation of minor loci of plant height. (**a**) and (**b**): GWAS of plant height assigning *Dt1* as a fixed effect in the mix line model. (**a**) Manhattan plots for plant height. Negative $\log_{10}$ *P*-values from a genome-wide scan are plotted against SNP positions of 20 chromosomes. (**b**) Quantile-quantile plot for plant height. (**c**) and (**d**): GWAS of plant height by multi-locus mixed-model approach. (**c**) Manhattan plots for plant height. Negative $\log_{10}$ *P*-values from a genome-wide scan are plotted against SNP positions of 20 chromosomes. (**d**) Quantile-quantile plot for plant height. The horizontal dash lines indicate the significant threshold (2×10^{-7}). Trait-associated SNPs above the significant threshold are colored in red.

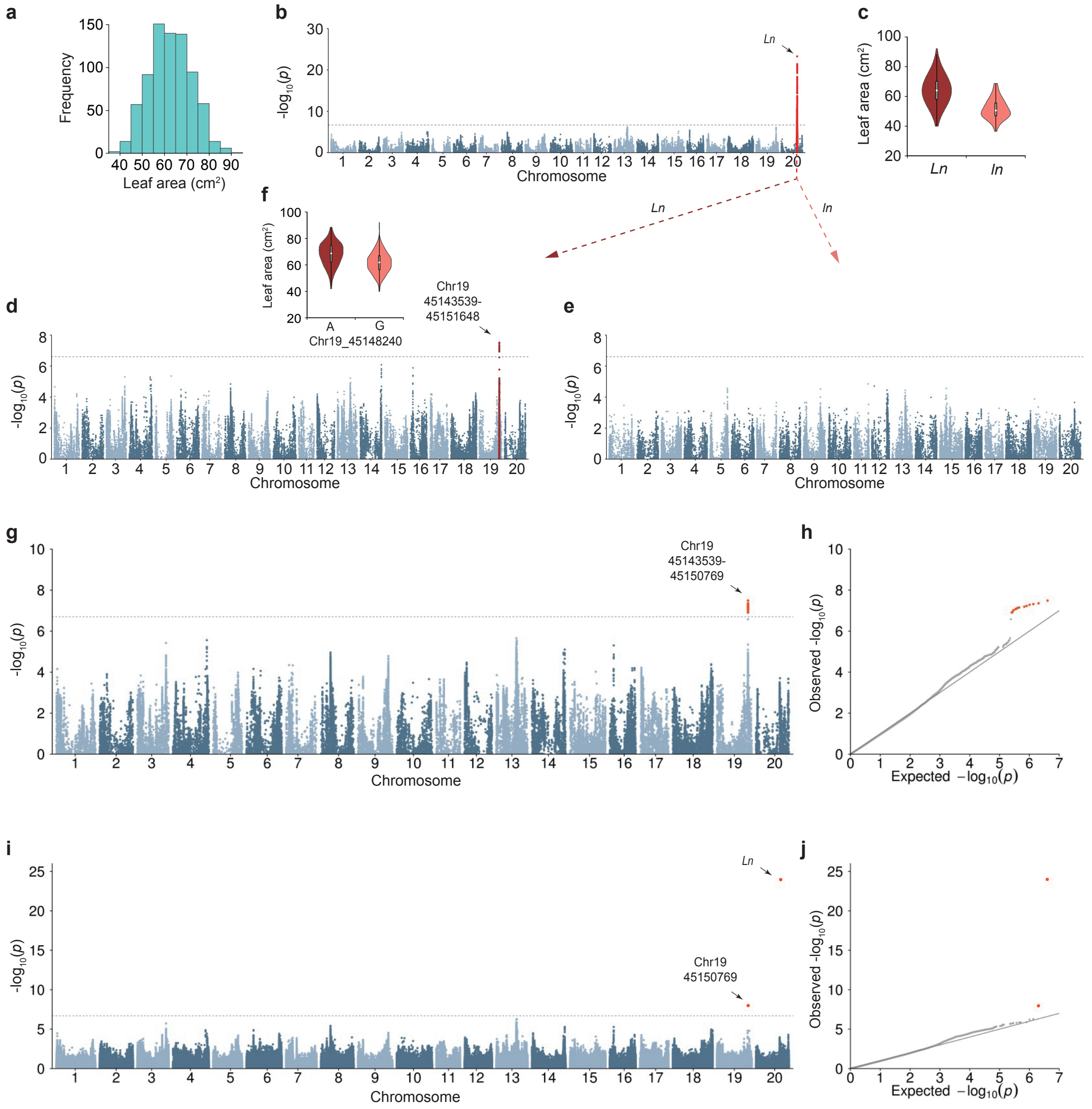

Supplementary Figure 88 Genome-wide association study (GWAS) of leaf area. (**a**) Leaf area distribution of different soybean accessions. (**b**) GWAS result from all accessions. Negative $\log_{10}$ *P*-values are plotted against the position on each of 20 chromosomes. (**c**) Leaf area variation between different *Ln* alleles. (**d**) GWAS of leaf area using the accessions only from the *Ln* subgroup. (**e**) GWAS of leaf area using the accessions only from the *ln* subgroup. (**f**) Leaf area variation between different genotypes at the association site from the *Ln* subgroup. (**g**) and (**h**): GWAS of leaf area assigning *Ln* as a fixed effect in the mix line model. (**g**) Manhattan plots for leaf area. Negative $\log_{10}$ *P*-values from a genome-wide scan are plotted against SNP positions of 20 chromosomes. (**h**) Quantile-quantile plot for leaf area. (**i**) and (**j**): GWAS of leaf area by multi-locus mixed-model approach. (**i**) Manhattan plots for leaf area. Negative $\log_{10}$ *P*-values from a genome-wide scan are plotted against SNP positions of 20 chromosomes. (**j**) Quantile-quantile plot for leaf area. Horizontal dashed lines indicate the genome-wide significance threshold (2×10^{-7}). Trait-associated SNPs above the significant threshold are colored in red.

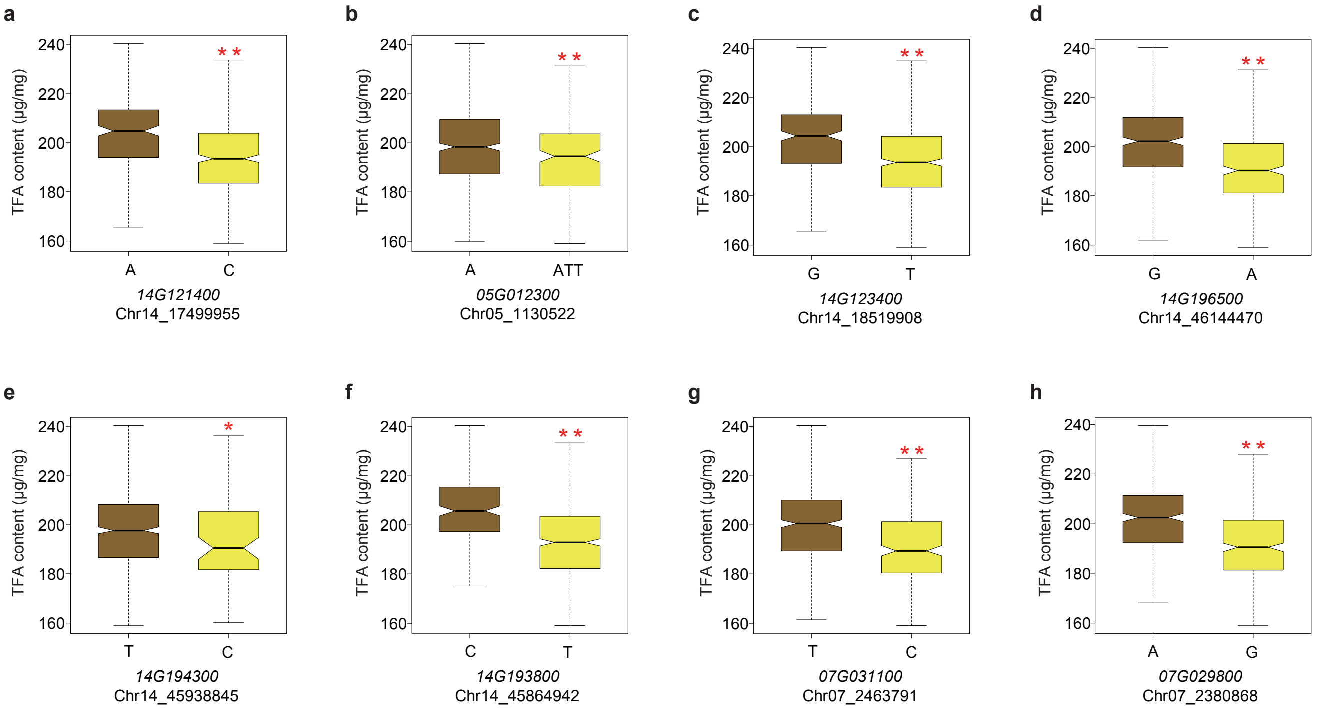

Supplementary Figure 89 Total fatty acid (TFA) content between different alleles of the candidate genes in fatty acid and Triacylglycerol (TAG) biosynthesis pathway. (**a**) *14G121400* (*n* = 461, 219). (**b**) *05G012300* (*n* = 202, 478). (**c**) *14G123400* (*n* = 456, 224). (**d**) *14G196500* (*n* = 308, 372). (**e**) *14G194300* (*n* = 69, 611). (**f**) *14G193800* (*n* = 473, 207). (**g**) *07G031100* (*n* = 248, 432). (**h**) *07G029800* (*n* = 321, 359). *P < 0.05; **P < 0.01 (one-sided Student's *t*-test).

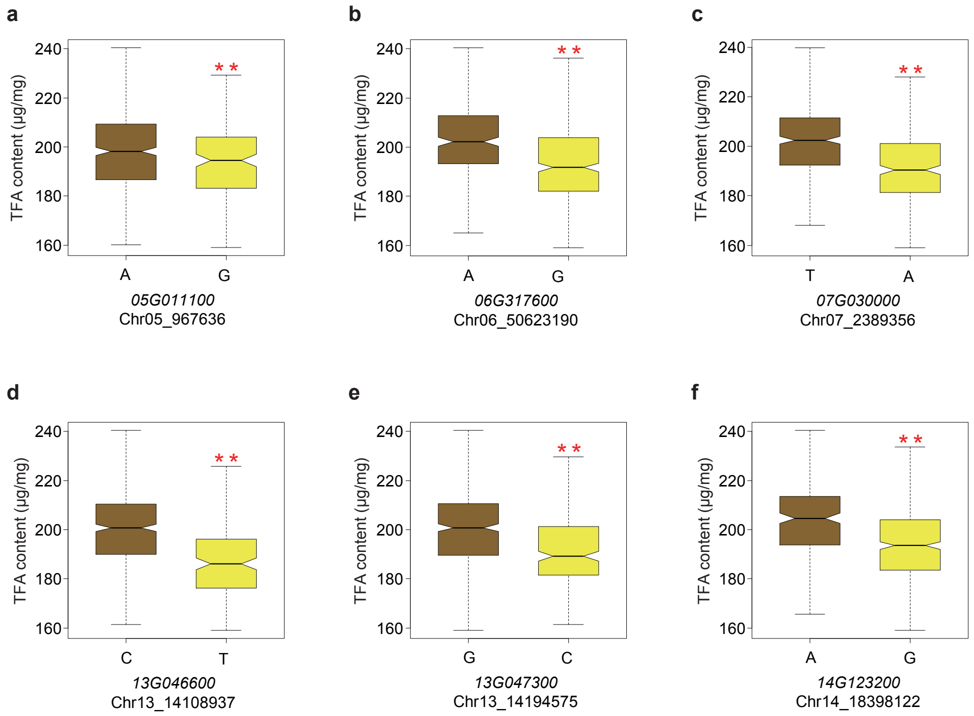

Supplementary Figure 90 Total fatty acid (TFA) content between different alleles of the candidate genes in lipid biosynthesis pathway. (**a**) *05G011100* (n = 168, 512). (**b**) *06G317600* (n = 379, 301). (**c**) *07G030000* (n = 321, 359). (**d**) *13G046600* (n = 178, 502). (**e**) *13G047300* (n = 245, 435). (**f**) *14G123200* (n = 462, 218). $^{**}P < 0.01$ (one-sided Student's t-test).

a

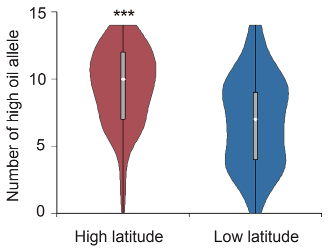

b

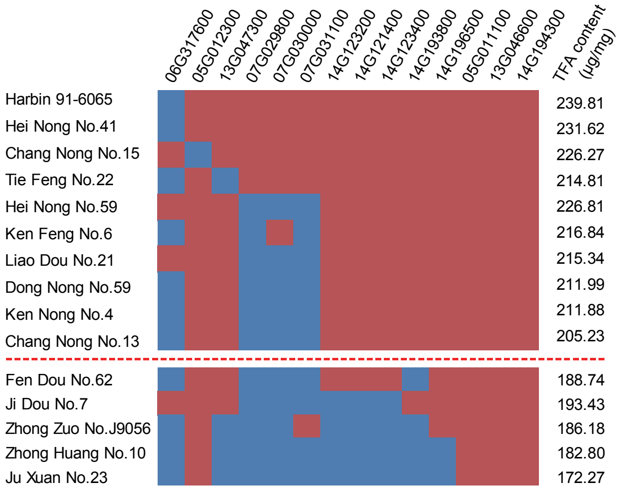

Supplementary Figure 91 Distribution of high oil allele number in different accessions. (**a**) High oil allele number in accessions from high latitude and low latitude. The difference of high oil allele number between high latitude (n = 219) and low latitude accessions (n = 461) is illustrated. ***P < 0.001 (one-sided Student's t-test). (**b**) High oil allele number in ten high-oil cultivars from Northeast China and five high-yield cultivars from Huanghuaihai Region. The red pane indicates high oil allele. The blue pane indicates low oil allele. The dotted line separates the high-oil and high-yield cultivars. TFA, total fatty acid.

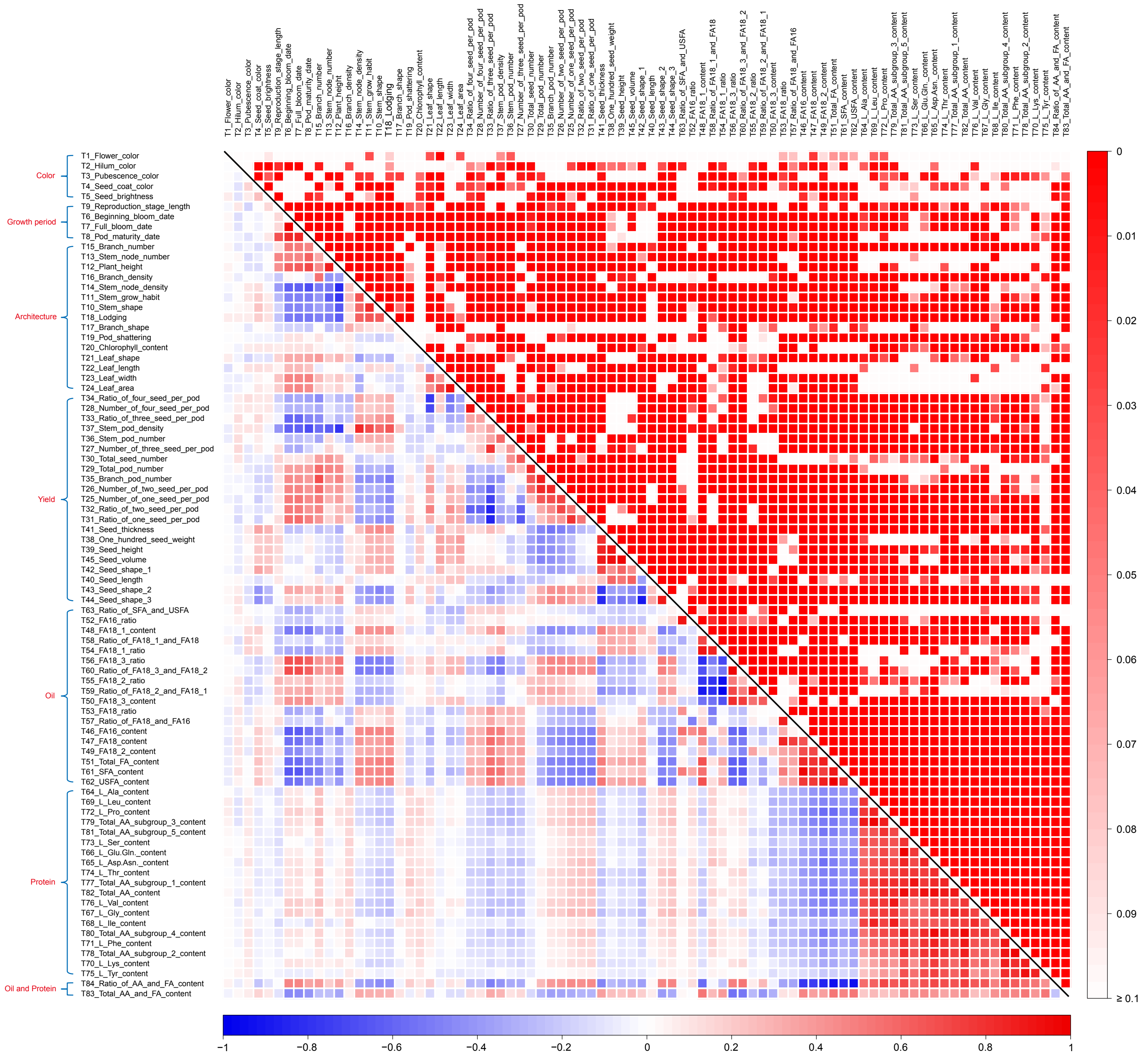

Supplementary Figure 92 Correlation across different traits. Upper triangular matrix indicates *P* value. Lower triangular matrix indicates correlation value.

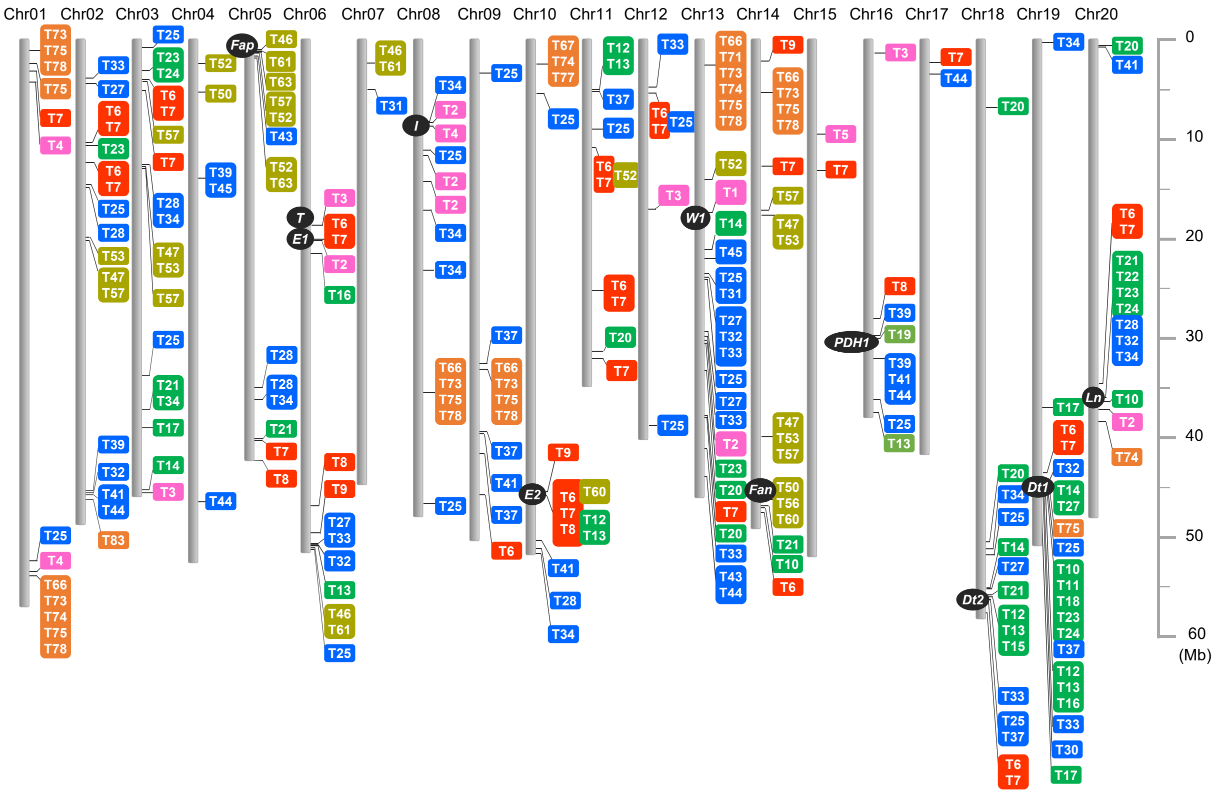

Supplementary Figure 93 Significantly associated loci (SAL) of the 57 traits across the soybean genome. The SAL are marked to the chromosomes based on the highest association position in the responsible GWAS signals. The trait names are abbreviated to fit the space limitation, and the abbreviated names of each trait match those in **Additional file 6: Table S5.** Traits related to color (T1-T5) are marked with magenta boxes, traits related to growth period (T6-T9) are marked with red boxes, traits related to architecture (T10-T24) are marked with green boxes, traits related to yield (T25-T45) are marked with blue boxes, traits related to oil (T46-T63) are marked with moss boxes, and traits related to protein (T64-T84) are marked with orange boxes. Some of the previously identified genes that are responsible for related agronomical traits are shown as black ovals in their physical positions on the chromosome.

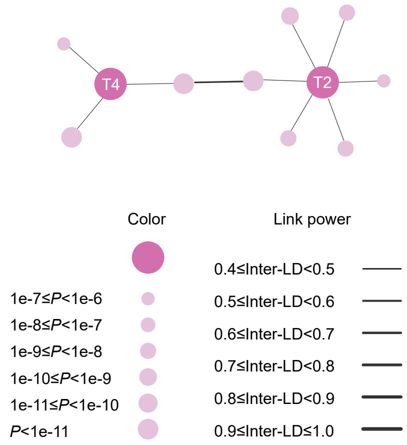

Supplementary Figure 94 Association network across color related traits. The nodes represent traits and their responsible significantly associated loci (SAL). The edge between SAL from different traits is linked by linkage disequilibrium (LD). Only the edge with average LD ≥ 0.4 is illuminated. The abbreviations of the traits match those in **Additional file 6: Table S5**.

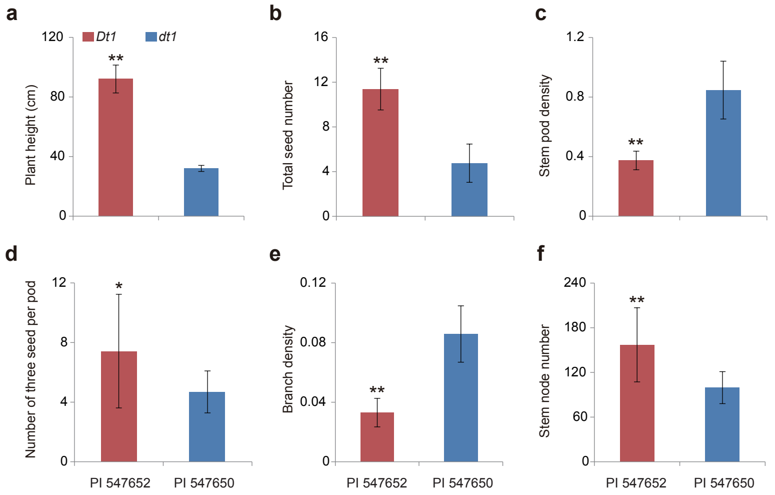

Supplementary Figure 95 Phenotyping differences between *Dt1 and dt1* near isogenic lines. (**a**) Plant height. (**b**) Total seed number. (**c**) Stem pod density. (**d**) Number of three seed per pod. (**e**) Branch density. (**f**) Stem node number. Phenotype data (mean ± s.d., n = 4) of *Dt1* (PI 547652, *Dt1E1e2e3t*) and *dt1* (PI 547650, *dt1E1e2e3t*) near isogenic lines are illustrated. *P < 0.05; **P < 0.01 (one-sided Student's t-test).

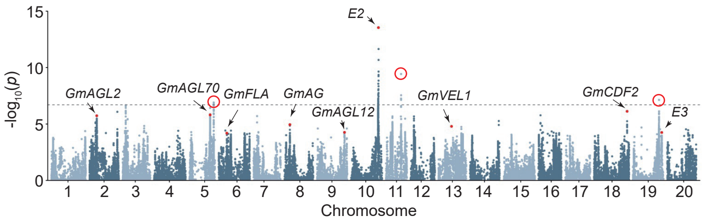

Supplementary Figure 96 Candidate genes of full bloom date identified by GWAS. Negative $\log_{10}$ *P*-values from a genome-wide scan are plotted against SNP positions of 20 chromosomes. The horizontal dash line indicates the significant threshold (2×10^{-7}). The candidate flowering time related genes in some association loci are listed (ref1). *GmAGL2* is homologous to *AGL2* in *Arabidopsis*. *GmAGL70* is homologous to *AGL70* in *Arabidopsis*. *GmFLA* is homologous to *FLA* in *Arabidopsis*. *GmAG* is homologous to *AG* in *Arabidopsis*. *GmAGL12* is homologous to *AGL12* in *Arabidopsis*. *GmVEL1* is homologous to *VEL1* in *Arabidopsis*. *GmCDF2* is homologous to *CDF2* in *Arabidopsis*. The association loci in red circles are newly identified in this study.

ref1: Chen, C. et al. PICARA, an analytical pipeline providing probabilistic inference about a priori candidates genes underlying genome-wide association QTL in plants. PLoS One. 2012;7**:**e46596.

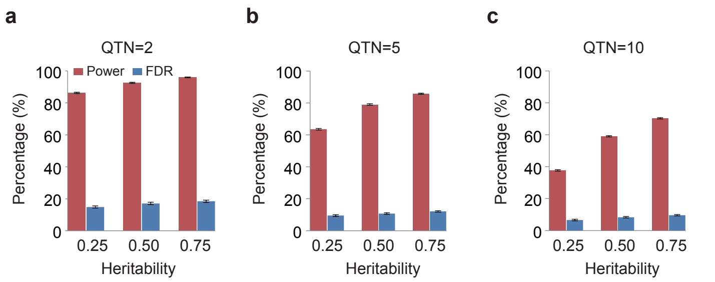

Supplementary Figure 97 Power and FDR in 2, 5, and 10-locus model simulations. Three sets of quantitative trait nucleotide (QTN) are used in the simulations: (**a**) QTN = 2 (left), (**b**) QTN = 5 (middle), (**c**) QTN = 10 (right). Power and false discovery rate (FDR) data (mean ± s.e., n = 1000) are illustrated at three heritability levels.

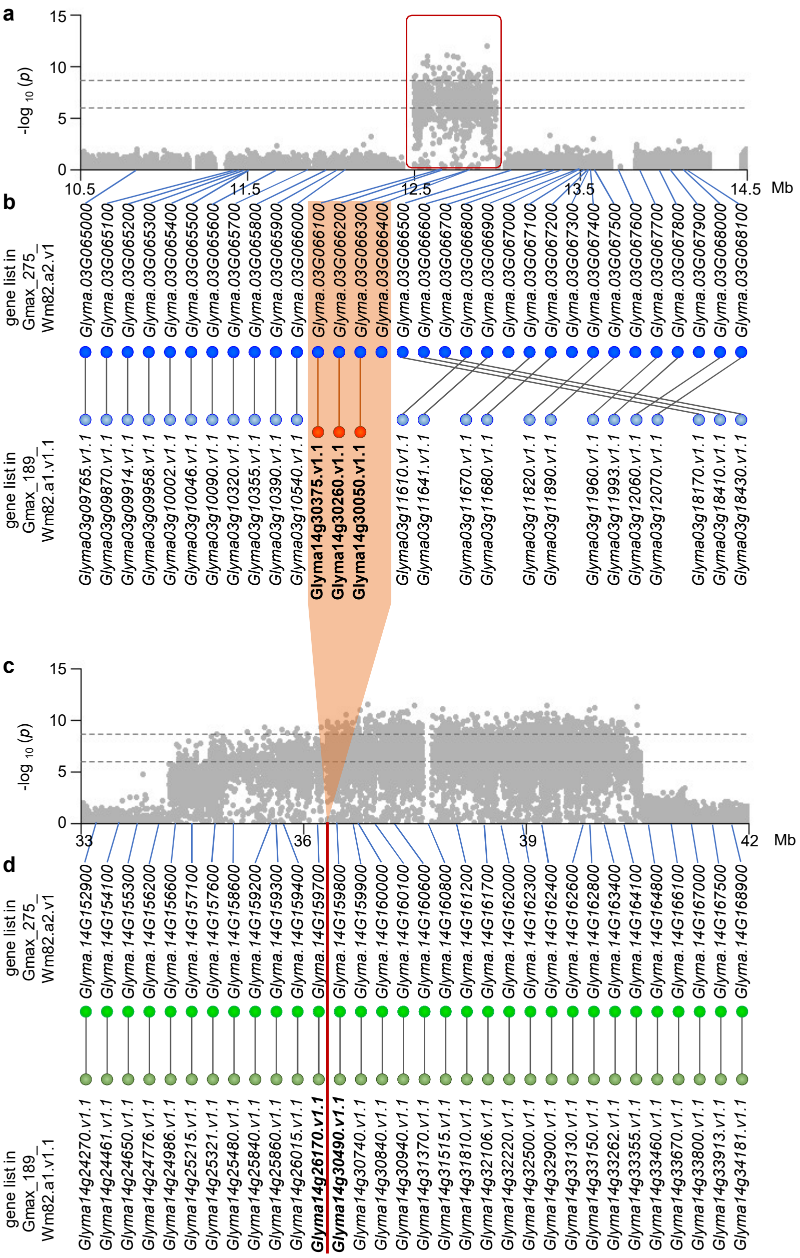

Supplementary Figure 98 An example of assemble problem of the soybean genome. (**a**) GWAS result of FA18 in Chromosome (Chr.) 3 from 10.5 to 14.5 MB (Wm82.a2.v1 v2.1). A significant association signal, which is covered by the red box, is detected. (**b**) Matched gene order between Gmax_275_Wm82.a2.v1 (current version) and Gmax_189_Wm82.a1.v1.1 (previous version). Three gene models of current version (*Glyma.03G066100*, *Glyma.03G066200*, *Glyma.03G066300*) match to genes from Chr.14 of previous version (*Glyma14g30375*.v1.1, *Glyma14g30260*.v1.1, *Glyma14g30050*.v1.1), respectively. The GWAS signal only covers the gene models matched to that from Chr.14. (**c**) GWAS result of FA18 in Chr.14 from 33 to 42 MB (Wm82.a2.v1 v2.1). A significant association signal is detected. (**d**) Matched gene order between Gmax_275_Wm82.a2.v1 (current version) and Gmax_189_Wm82.a1.v1.1 (previous version). The gene models between *Glyma14g26170*.v1.1 and *Glyma14g30490*.v1.1 of previous version, which includes *Glyma14g30375*.v1.1, *Glyma14g30260*.v1.1, *Glyma14g30050*.v1.1, are not orderly matched to the current gene models. The results indicated that the gene models (*Glyma.03G066100*, *Glyma.03G066200*, *Glyma.03G066300*, *Glyma.03G066400*) were wrongly assembled at current version genome. They should be at Chr.14.
